# A deeper look at long-term effects of COVID-19 on myocardial function in survivors with no prior heart diseases: a GRADE approach systematic review and meta-analysis

**DOI:** 10.3389/fcvm.2024.1458389

**Published:** 2024-11-19

**Authors:** Mahshid Dehghan, Seyedeh-Tarlan Mirzohreh, Raheleh Kaviani, Shiva Yousefi, Yasaman Pourmehran

**Affiliations:** ^1^ Faculty of Medicine, Tabriz University of Medical Sciences, Tabriz, Iran; ^2^ Shahid Rajaie Cardiovascular Medical and Research Center, Iran University of Medical Sciences, Tehran, Iran; ^3^ Department of Stem Cell and Developmental Biology, Cell Science Research Center, Royan Institute for Stem Cell Biology and Technology, ACECR, Tehran, Iran

**Keywords:** COVID-19, long-term effects, myocardial function, GRADE approach, systematic review, meta-analysis

## Abstract

**Objectives:**

The COVID-19 pandemic has challenged global health systems since December 2019, with the novel virus SARS-CoV-2 causing multi-systemic disease, including heart complications. While acute cardiac effects are well-known, long-term implications are understudied. This review hopes to fill a gap in the literature and provide valuable insights into the long-term cardiac consequences of the virus, which can inform future public health policies and clinical practices.

**Methods:**

This systematic review was prepared using PRISMA reporting guidelines. The databases searched were PubMed, Scopus, Web of Science, and Cochrane. Risk of Bias was assessed using ROBINS-I. The GRADE approach was employed to evaluate the level of certainty in the evidence for each outcome. A meta-analysis was conducted using the Comprehensive Meta-Analysis (CMA) software. In order to identify the underlying cause of high heterogeneity, a subgroup analysis was conducted. Sensitivity analysis was checked.

**Results:**

Sixty-six studies were included in this review. Thirty-two of them enrolled in meta-analysis and the rest in qualitative synthesis. Most outcomes showed a moderate certainty of evidence according to the GRADE framework. Post-COVID individuals with no prior heart diseases showed significant changes in left ventricular (LV) and right ventricular (RV) echocardiographic indices compared to controls. These significant findings were seen in both post-acute and long-COVID survivors regardless of the severity of initial infection.

**Conclusion:**

This review implies that individuals recovering from post-acute and long-term effects of COVID-19 may experience changes in myocardial function as a result of the novel coronavirus. These changes, along with cardiac symptoms, have been observed in patients without prior heart diseases or comorbidities.

**Systematic Review Registration:**

PROSPERO, identifier (CRD42024481337).

## 1 Introduction

Since the beginning of December 2019, the COVID-19 pandemic, caused by a novel virus known as SARS-CoV-2 that originated in Wuhan, China, has presented a major challenge to global health and healthcare systems. Although COVID-19 is predominantly associated with lung-related symptoms and distinct functional and morphological changes, it has become evident that the infection can also lead to a multi-systemic disease affecting various organs including heart (1–3).

There is growing evidence of COVID-19's harmful effects on the heart, including acute events like heart attacks and long-term consequences even after recovery. The precise mechanisms underlying the cardiac damage caused by COVID-19 remain incompletely understood. COVID-19 has been associated with various patterns of cardiovascular dysfunction, including myocarditis, ischemic heart disease (e.g., myocardial infarction), hypovolemia, RV dysfunction resulting from pulmonary embolism, and, in some cases, cardiovascular dysfunction due to superimposed bacterial or fungal sepsis (4). The pathological findings suggest that SARS-CoV-2 can trigger hyper myocardial inflammation by infecting cardiomyocytes, leading to myocyte necrosis. This, in turn, may contribute to an increased risk of acute myocardial infarction, heart failure, arrhythmia, cardiac arrest, and acute coronary syndrome. Furthermore, apart from the potential harm caused by the illness itself, certain medications used in the treatment of COVID-19 and drug interactions may also have specific side effects on the heart (5).

Despite the advancements in treatments for COVID-19, it is anticipated that long-term consequences of the disease, particularly those affecting the heart, will persist in survivors. Therefore, the investigation of myocardial dysfunction following recovery from COVID-19 plays a vital role in the development of post-discharge monitoring programs and the formulation of public health, economic, and social policies (6). Cardiac imaging studies can serve as valuable predictive tools and aid in the comprehension of the underlying mechanisms of cardiac involvement. Echocardiography has been recognized as an available, non-invasive, and informative diagnostic tool, to identify cardiac manifestation (7). Echocardiographic findings in individuals with COVID-19 may exhibit variability, ranging from specific regional wall motion abnormalities of the LV or RV to varying degrees of global cardiac dysfunction associated with myocarditis or a systemic dysregulated inflammatory response to viral infection. Echocardiography is therefore essential in distinguishing these patterns, guiding treatment strategies, and monitoring the clinical progression over time (8).

Existing research has extensively focused on the impact of acute COVID-19 on cardiac function and complications, with numerous reviews and studies providing valuable insights into this area (9). However, there is a lack of information in the research on the lasting impacts of COVID-19, known as long covid, on heart function. Previous reviews have tried to examine this connection, but they have been restricted in their coverage and have not carried out thorough meta-analyses. Furthermore, these reviews have not taken into consideration possible influencing factors like the severity of the initial COVID-19 infection, the time elapsed since the infection, and the presence of other medical conditions. Hence, it is essential to conduct more in-depth and thorough research that specifically looks into the lasting effects of COVID-19 on the heart, considering important factors. To tackle this issue, we carried out a systematic review and meta-analysis that focused on echocardiographic imaging to study the long-term impact of SARS-CoV-2 infection on heart function and the risk of future cardiac complications.

## 2 Materials and methods

This systematic review and meta-analysis follows the Preferred Reporting Items for Systematic Reviews and Meta-Analyses (PRISMA) guidelines (10). The PRISMA checklist is provided as supplement (Supplementary S1 document). The protocol for this work was registered in the International Prospective Register of Systematic Reviews (PROSPERO) (identifier: CRD42024481337). This review also followed the published protocol for evaluating risk factors and prognostic implications of imaging left ventricular diastolic dysfunction in individuals diagnosed with COVID-19, adhering to a systematic approach (8).

### 2.1 Eligibility criteria

To be considered for inclusion, published studies had to meet the following criteria: (1) studies employed valid research designs with clearly defined methodology, (2) studies assessed cardiac function using Echocardiography in COVID-19 patient after recovery, (3) studies identified COVID-19 infection according to the World Health Organization interim guidance, (4) studies reported at least one echocardiographic parameter measuring myocardial function and/or structure, (5) studies excluded cases with pre-existing cardiac disease including ischemic heart disease, valvular disease, arrhythmias-conduction disorders, heart failure, cardiomyopathies, pericarditis, pericardial effusion, pulmonary hypertension, pulmonary embolism, sarcoidosis, amyloidosis, active cancer, recent pregnancy, postpartum. The overall exclusion criteria were as follows: (1) studies involved cases during acute stage of COVID-19, (2) studies evaluated cardiac function using any other imaging technique other than echocardiography, (3) studies reported as abstracts, case reports, case series, reviews, or practice guidelines.

### 2.2 Information sources

A thorough search was conducted in the PubMed, Scopus, Web of Science, and Cochrane databases to locate relevant studies published until March 2024. Additionally, a manual search of the reference lists of the identified articles was carried out.

### 2.3 Search strategy

The search strategy of Scopus was conducted as follows: (TITLE-ABS-KEY (((“left ventric*” OR “right ventric*” OR “left cardiac*” OR “right heart” OR “right cardiac” OR “left heart” OR atri* OR myocardi* OR diastol* OR systol*) PRE/1 (dysfunction OR function OR remodeling OR impair* OR hypertroph* OR active* OR volume OR mass* OR dimension* OR diameter OR thickness OR index* OR “ejection time” OR “ejection fraction”)) OR echo OR echocardiograph*)) AND [TITLE-ABS-KEY (“covid-19” OR “sars cov 2”)]. The search strategy employed for PubMed, Web of Science, and the Cochrane Library was similar to that used for Scopus and its table is provided as supplement (Supplementary S2 document). Furthermore, three reviewers independently reviewed the reference lists of systematic reviews and selected studies to ensure that all pertinent articles were included in the analysis.

### 2.4 Study selection

Three reviewers independently assessed each title and abstract, and if the articles fulfilled the inclusion criteria, the full text was reviewed. The eligibility of the selected articles was then assessed by the same three reviewers through an evaluation of their full texts. Any discrepancies were resolved through discussion with a fourth reviewer. The study selection process was summarized using the PRISMA flow diagram.

### 2.5 Data extraction and data items

Following the extraction of data, the information was gathered through Microsoft Excel spreadsheets. The subsequent dataset comprises: studies' basic characteristics (study design, year of publication, country, and first author), participant characteristics (age, body mass index, number of cases and control groups), echocardiographic indices and major findings of each study. Potential confounding factors were carefully considered to ensure the robustness of the study findings. These factors included severity of COVID-19 infection, persistent post-COVID symptoms, duration from COVID acute phase to echocardiography examination in recovery phase, presence of comorbid disease. Data related to these factors were extracted from the studies to address their potential influence on the findings.

### 2.6 Risk of bias assessment

ROBINS-I was employed to evaluate the methodological quality and risk of bias for non-randomized control trials. This tool encompasses the assessment of seven potential sources of bias, including confounding bias, bias in participant selection, bias in intervention classification, bias due to deviations from intended interventions, bias resulting from missing data, bias in outcome measurement, and bias in the selection of reported results (11). Importantly, no studies were excluded based on the assessment of bias risk.

### 2.7 Outcome quality assessment

The certainty of overall evidence was assessed using the Grading of Recommendations Assessment, Development and Evaluation (GRADE) method (12). The assessment of evidence certainty for individual outcomes relied on five distinct criteria: (1) limitations of the study design; (2) consistency of results; (3) directness; (4) precision and (5) potential for publication bias. A decrement of one level in certainty was implemented for each unfulfilled criterion.

### 2.8 Synthesis methods

The mean differences (MD) pooled the data, with 95% confidence intervals (CIs). The *I*^2^ statistic was used to analyze the interstudy statistical heterogeneity. To calculate the pooled effect, either fixed-effects or random-effects model was used according to the heterogeneity, study design and sample size. *I*^2^ values of 25%, 50%, and 75% were considered to represent low, moderate, and high levels of heterogeneity, respectively. Subgroup meta-analyses were conducted to uncover the underlying heterogeneity. A univariate meta-regression model was used to explore the impact of age and BMI as potential moderators. A sensitivity analysis was carried out in cases where the decision-making values had arbitrary or unclear ranges. Publication bias was assessed by visually inspecting funnel plots of MD vs. standard error. When at least 10 studies were available for analysis, Begg's tests and Egger's tests were employed to evaluate the potential publication bias. If there was an obvious publication bias, a trim-and-fill analysis was used to determine the underlying origin of the publication bias. All analyses were conducted using Comprehensive Meta-Analysis Version 3. *P*-value < 0.05 was considered significant in all tests.

## 3 Results

### 3.1 Study selection

**Figure 1:**
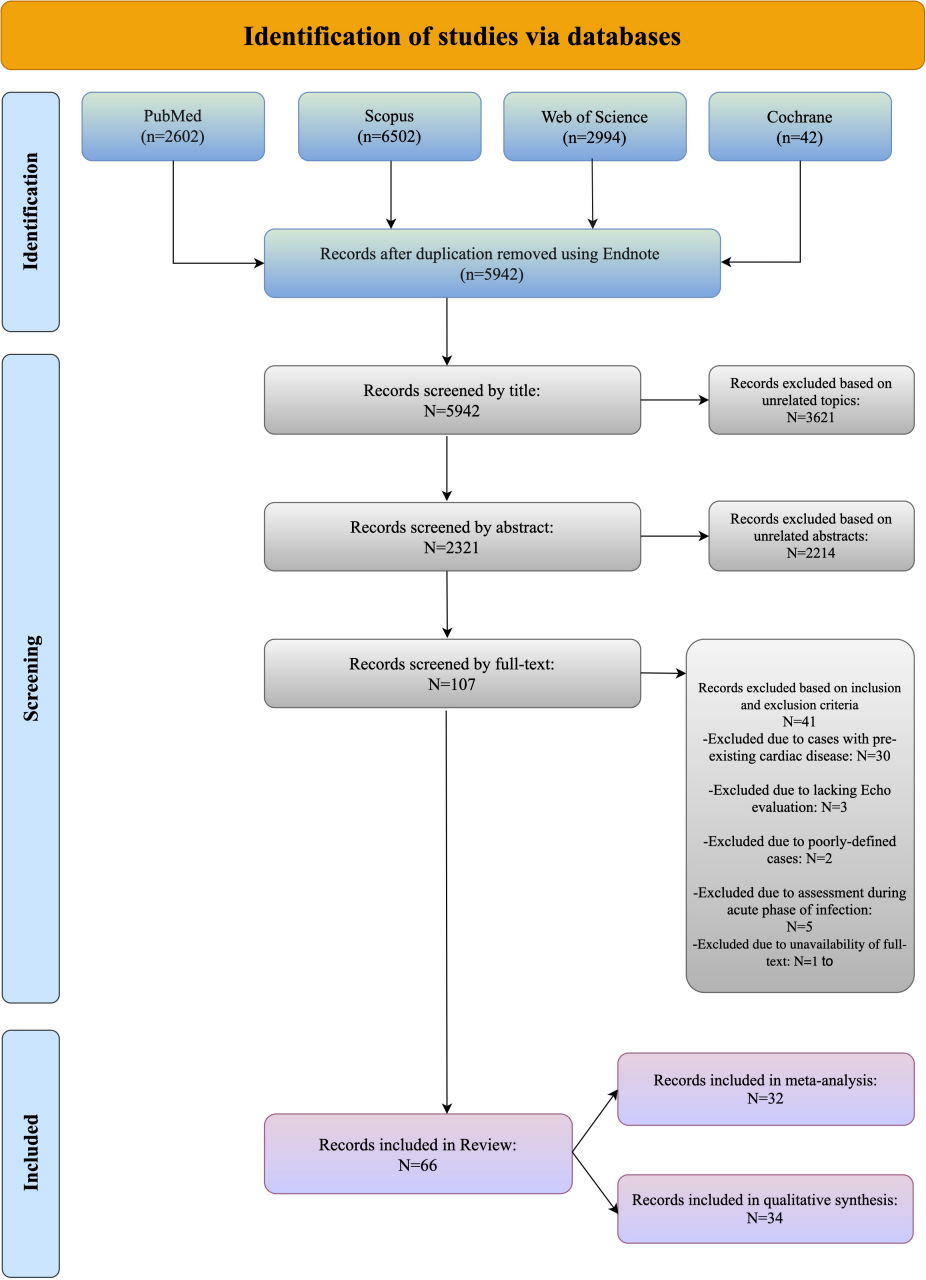
Identification of studies via databases.

The study flowchart is shown in Figure 1; our search strategy revealed 2,602 studies in PubMed, 6,502 in Scopus, 2,994 in WOS and 42 in Cochrane. After removing duplications, 5,942 studies underwent title assessment. Of these, 2,321 studies were eligible for abstract review. After surveying abstracts, 107 studies were perused for full text. Finally, 66 studies were qualified to be included in this systematic review and meta-analysis, and the rest did not meet the inclusion criteria; the reasons for their exclusions are provided in the supporting information section (Supplementary S3 document).

### 3.2 Characteristics of studies

Table 1 presents the key features of the sixty-six studies (13–76) included in this research. The search process resulted in the identification of 66 studies, out of which 41 were designed as cohort studies, 16 were cross-sectional studies, 8 were case-control studies, and one study (20) was a combined cross-sectional and longitudinal cohort. The majority of these studies utilized real-time PCR (rt-PCR) as the diagnostic method for COVID-19, while a few employed the IgG antibody titer for diagnosis (32, 37). Most of the studies focused on adult patients who had recovered from COVID-19, whereas 6 studies specifically examined athletes who had overcome the disease (13, 17, 19, 29, 52, 75). A total of 32 studies were conducted comparing post-COVID patients to a control group of individuals who tested negative for COVID-19. All 32 studies were included in a meta-analysis, with the exception of one study (39) where patients and controls were not matched, and two studies (42, 43) where matching status was unknown. Four studies (16, 27, 28, 40) categorized COVID-19 cases into groups based on the severity of the infection. To maintain consistency with the other included studies, we treated these studies as separate entities, each having a common healthy control group. Furthermore, two studies (30, 43) assessed COVID-19 cases based on the presence of dyspnea symptoms in patients. These studies were also divided into two distinct studies. Three studies (16, 24, 37) divided COVID-19 cases based on the time duration between diagnosis and echocardiography. Similarly, one study (17) divided COVID-19 cases into male and female athletes. Each of these studies was separated into two distinct studies as well.

**Table 1:** Characteristics of included studies.

First author, year, Country	Study design	Subgroup	Covid-19 patient category	Severity of Covid-19 infection	Covid-19 vaccination	Symptoms at study enrollment	Duration from acute COVID to Echo examination in recovery phase	No. Covid-19 cases/groups	Echocardiographic parameters	Comorbid disease	Main findings
Studies with post-COVID cases compared to non-COVID control group (meta-analysis)											
Duration from acute COVID to echo examination in recovery phase (<1 Month)											
Lakatos et al. (2021) Hungary (13)	Case-control	None	Athletes	Mild	Unknown	Asymptomatic (88.7%), loss of taste and/or smell (11.2%)	Mean 22 days (17–25)	107/107 matched	IVSD, PWD, LAVI, mitral E wave, mitral A wave, E/A, E/e’, RVD, TAPSE, LVEF, LVGLS	None (excluded from the study)	LV systolic and diastolic impairment in post-COVID athletes compared to control group. (↑E/A, ↓E/e’, ↓PWD, ↓IVSD, ↑LVEF)
Duration from acute COVID to echo examination in recovery phase (1–3 Months)											
Günay et al. (2021) Turkey (14)	Prospective cohort	None	Adults	Moderate and Severe	Unknown	None	1 months	51/32 matched	LVESD, LVEDD, LAD, LVEF, IVSD, PWD, mitral E wave, mitral A wave, RV-GLS, RVD, sPAP, TAPSE, RV-MPI	None (Excluded from the study)	RV impairment in post-COVID patients compared to control group. (↑RV-MPI, ↑RV end-diastolic, end-systolic area and ↑sPAP)
Gul et al. (2022) Turkey (15)	Cross-sectional	None	Adults	Mild and Moderate	Unknown	None	58.39 ± 39.1 days (10–180)	126/98 matched	LVEF, LAD, LVEDD	HTN: 18.3% smoking: 30.2%	LV systolic impairment in post-COVID athletes compared to control group. (↓LVEF, ↑LVEDD)
Tryfou et al. (2021) Greece (16)	Prospective cohort	Tryfou et al. (2021) ^(1)^	Hospitalized adults	Moderate and severe	Unknown	Not mentioned	At least 10 days (10–29)	67/37 matched	IVSD, PWD, LVEDD, LVESD, E/A, E/E’, LVEF, LVGLS, RVGLS	HTN, T2DM, Hyperlipidemia, smoking	LV and RV systolic impairment (↓LV-GLS, ↓RV-GLS)
		Tryfou et al. (2021) ^(2)^	Non- hospitalized adults	Mild			1.5 month	33/37 matched			LV and RV systolic impairment (↓LV-GLS, ↓RV-GLS)
Turpin et al. (2023) USA (17)	prospective, case-control	Turpin et al. (2023) ^(1)^	Female athletes	Asymptomatic and mild	Unknown	None	1.5–3 months (51 ± 43)	24/24 matched	IVSD, PWD, LVM, LVMI, LVEDD, LVEDV, LVEF, mitral E wave, mitral A wave, E/A, E/e’	None (excluded from the study)	No significant finding
		Turpin et al. (2023) ^(2)^	Male athletes				1–2 months (31 ± 32)	37/37 matched			
Kurtoğlu et al. (2022) Turkey (18)	Retrospective cohort	None	Adults	Mild	Unknown	None	At least 2 months (5 ± 2.8)	50/50 matched	LAD, LVEF, LVEDD, LVESD, IVSD, PWD, E/A, E/e’	None (Excluded from the study)	No significant finding
Schellenberg et al. (2023) Germany (19)	Prospective cohort	None	Athletes	Not mentioned	Unknown	Fever, cough, rhinorrhea, sore throat, resting dyspnea, exertional dyspnea, palpitation, chest pain, increased resting heart rate, dizziness, subjective perceived performance limitation	2 months	88/52 matched	LVM, LVEF, E/e', E/A	None (Excluded from the study)	LV systolic and diastolic impairment in post-COVID athletes compared to control group. (↑LVEF, ↓LVM, ↑E/e’, ↓E/A)
Honchar et al. (2023) Ukraine (20)	Combined cross-sectional and longitudinal cohort	None	Hospitalized adults	Moderate and severe	Unknown	Not mentioned	1.5–2 months	176/88 matched	LAD, IVSD, PWD, LVEDD, LVESD, LVMI, LVEF, LV-GLS, mitral e’ wave, mitral E wave, E/A, E/e’, RAD, RVD, TAPSE	HTN, T2DM, smoking	LV systolic and diastolic, RV diastolic impairment in post-COVID athletes compared to control group. (↑LAVI, ↑IVSD, ↑PWD, ↓LVEDD, ↓LVESD, ↑LVMI, ↓LV-GLS, ↓TAPSE)
Mahajan et al. (2021) India (21)	Prospective cohort	None	Adults	Mixed (mild, moderate, and sever)	Unknown	Palpitations, dyspnea, fatigue, cough, syncope, pedal oedema, fever	1–1.5 month	94/40 matched	LV-GLS	T2DM, HTN, Smoking	LV systolic impairment in post-COVID athletes compared to control group (↓LV-GLS) RV impairment in impairment in post-COVID athletes with reduced LV-GLS compared to in post-COVID athletes with Normal LV-GLS. (↓TAPSE, ↑RVD)
Turan et al. (2021) Turkey (22)	Prospective cross-sectional	None	Adults	Asymptomatic and mild	Unknown	None	1 month (11–89 days)	70/70 matched	LVGLS, LVEF, LVEDD, LVESD, LVEDV, LVESV, IVSD, PWD, LAD, E, A, E’, A′, E/E’, RAD, RVD, TAPSE	HTN, Smoking, Asthma, Alcohol addiction	LV systolic and diastolic impairment in post-COVID patients compared to control group. (↓LV-GLS, ↑A wave)
Ardahanli et al. (2022) Turkey (23)	Cross-sectional	None	Adults	Mild and moderate	Unknown	Exertional dyspnea, chest pain, palpitation	2 months	200/182 matched	LVEDD, PWD, IVSD, LV-MPI, E mitral, A mitral, E/A, TAPSE, RV diameter	None (excluded from the study)	RV and LV systolic impairment in post-COVID patients compared to control group. (↑LV-MPI, ↓TAPSE)
Duration from acute COVID to echo examination in recovery phase (≥3 Months)											
De et al. (2023) India (24)	Cross-sectional	De et al. (2023) (24)	Adults	Majority was mild	First dose	Asymptomatic: 16.5% palpitations: 3.6% exertional dyspnea: 60.4% Chest pain: 5.9%Weakness:11.9%	>3 months	232/100 matched	LVEF, LV-GLS, sPAP, E/e’	HTN: 59.1% T2DM: 34.1% Pre-existing Airway obstruction:5.6%	LV systolic and diastolic impairment in post-COVID patients compared to control group. (↓LVEF, ↓LV-GLS, ↓E/e’)
Baltodano-Arellano et al. (2021) Peru (25)	Cross-sectional	None	Adults	Mild	Unknown	None	3–6 months	33/31 matched	LVEF, LV-GLS	None (excluded from the study)	LV systolic impairment in post-COVID athletes compared to control group (↓LV-GLS)
Akkaya et al. (2021) Turkey (26)	Cross-sectional	None	Adults	Mild	Unknown	None	3 months	105/105 matched	LVEDD, LVESD, LVEF, E/e’, PWD, IVSD, LAD, RV-GLS, RV MPI, TAPSE, sPAP, RV diameter	None (excluded from the study)	RV systolic and diastolic impairment in post-COVID athletes compared to control group (↓RV-GLS, ↑ RV-MPI, ↓TAPSE, ↑sPAP, ↑RV diameter)
Akbulut et al. (2022) Turkey (27)	Prospective cohort	Akbulut et al. (2022) (1)	Adults	Moderate and Severe	Unknown	None	6 months	16/20 matched	LVEDD, LVESD, LVM, E mitral, A mitral, E/e’, PWD, IVSD, LV-GLS, RV-GLS, TAPSE, LVEF	HTN, T2DM, Smoking	No significant finding
		Akbulut et al. (2022) (2)		Mild				42/20 matched	LVEDD, LVESD, LVM, E mitral, A mitral, E/e’, PWD, IVSD, LV-GLS, RV-GLS, TAPSE, LVEF		
Baykiz et al. (2021) Turkey (28)	Prospective cohort	Baykiz et al. (2021) (1)	Adults	Mild	Unknown (29)	None	6 months	34/44 matched	LAD, LVEDD, LVEDV, LVEF, RAD, RAD, E/e’, E/A, TAPSE, sPAP, LAVI, LV-GLS	HTN (35%), T2DM (15%), Smoking (23.5%)	RV and LV diastolic impairment in post-COVID athletes compared to control group. (↑LAD, ↓LV-GLS, ↓TAPSE, ↑sPAP)
		Baykiz et al. (2021) (2)	Adults	Moderate		None		30/44 matched		HTN (23%), T2DM (20%), Smoking (27%)	
		Baykiz et al. (2021) (3)	Adults	Severe		None		11/44 matched		HTN (36%), T2DM (18%), Smoking (9%)	
Beaudry et al. (2022) Canada (30)	Cross-sectional	Beaudry et al. (2022) (1)	Adults	Not mentioned	Vaccinated patients excluded from the study	Dyspnea	At least 3 months (219 ± 82 days)	16/16 matched	E/e’, TAPSE	Smoking (14%) Pre-existing Airway obstruction (17%) Cardiovascular Comorbid except obesity and CAD (10%)	No significant finding
		Beaudry et al. (2022) (2)		Not mentioned		None		16/16 matched	E/e’, TAPSE	Smoking (14%) Pre-existing Airway obstruction (7%) Cardiovascular Comorbid except obesity and CAD (14%)	
Gherbesi et al. (2022) Italy (31)	Retrospective cohort	None	Young adults	Mild	Unknown	None	At least 3 months (15 ± 1.4 weeks)	40/40 Matched	LVEDD, LVESD, LVEF, IVSD, PWD, LAVI, LVMI, E/e’, E/A, LV-GLS, sPAP, TAPSE, RV-GLS, RVD	None (Excluded from the study)	LV systolic impairment in post-COVID athletes compared to control group (↓LV-GLS)
Gumanova et al. (2023) Russia (32)	Cross-sectional	None	Adults	Not mentioned	Unknown	None	At least 3 months	70/237 matched	LAD, LVEF, LVEDD, LVESD, LVESV, LVM, LVMI, LVEDV, PWD, IVSD, LAVI, sPAP, E/A, E/e’	None (excluded from the study)	RV and LV diastolic impairment in post-COVID athletes compared to control group. (↑LAD, ↑LVEDD, ↑LVESV, ↑LVEDV, ↑LVM, ↑LVMI ↑sPAP, ↑E/A, ↑E/e’)
Küçük et al. (2022) Turkey (33)	Cross-sectional	None	Adults	Moderate and Severe	Unknown	None	3–6 months	50/50 matched	LAD, LVEF, LVEDD, LVESD, LV-GLS, IVSD, PWD, TAPSE, sPAP, E/A, RVD, RAD	None (Excluded from the study)	LV systolic impairment in post-COVID athletes compared to control group (↓LV-GLS)
Lambadiari et al. (2021) Greece (34)	Case-control prospective	None	Adults	Mild (34.4%) Moderate (32.8%) Sever (32.8%)	Unknown	No symptoms (62.9%), Fatigue (15.71%), dyspnea (12.8%), Cough (4.3%), chest pain (4.3%)	4 months	70/70 Matched	LV-GLS, RV-GLS, TAPSE	None (excluded from the study)	LV systolic, RV systolic and diastolic impairment in post-COVID athletes compared to control group. (↓LV-GLS, ↓RV-GLS, ↓TAPSE)
Barros et al. (2023) Brazil (35)	Retrospective cohort	None	Adults	Severe	Unknown	None	11.9 ± 7.0 Months	35/26 Matched	LVEF, RV-GLS, TAPSE, RVD	HTN (62.3%) DM (19.7%) CKD (3.3%) Excluded disease: chronic lung diseases, PAH prior to COVID-19, previously known RV echo	RV systolic impairment in post-COVID athletes compared to control group (↓RV-GLS)
Tudoran et al. (2023) Romania (36)	Case-control	None	Adult women (18–55)	Majority was mild and few was moderate	Unknown	Dyspnea, persistent cough, unexplained and long-lasting fatigue, reduced effort capacity, tachycardia, chest pain, increased BP values, insomnia, vertigo, concentration difficulties, and memory impairments	3 months	54/40 matched	LVEF, LVMI, LAVI, E/A, E/e’	None (Excluded from the study)	LV systolic and diastolic impairment in post-COVID athletes compared to control group (↓LVEF, ↓LV-GLS, ↑LVMI, ↑LAVI, ↓E/A. ↑ E/e’)
Yang et al. (2022) China (37)	Case-control	Yang et al. (1)	Adults	Not mentioned	Unknown	Palpitation (10%), angina pectoris (10%), vertigo (15%)	3 months	40/40 Matched	LV-GLS, TAPSE	HTN	No significant finding
		Yang et al. (2)					6 months				
		Yang et al. (3)					6 months				
Rácz et al. (2022) Hungary (38)	Case-control	None	Adults	Mild	Unknown	Chronic fatigue, difficulty of carrying out previously undemanding physical activity, and palpitations	3 months	86/60 Matched	LAD, LAVI, LVEDD, LVESD, LVEDV, LVESV, ISVD, PWD, LVEF, LV-GLS, mitral E wave, mitral A wave, E/A, E/e’, RAD, RVD	HTN, mixed connective tissue disease	LV systolic impairment in post-COVID athletes compared to control group. (↓LVEF, ↓LV-GLS, ↑LVEDD)
Rajotiya et al. (2024) India (39)	Prospective case-control	None	Adults	Severe	Unknown	Not mentioned	21 months	23/20 Not matched	LVEF	Smoking, alcohol consumption	LV systolic impairment in post-COVID athletes compared to control group. (↓LVEF)
Ozer et al. (2021) Turkey (40)	Prospective case-control	Ozer et al. (2021) (1)	Hospitalized adults	Moderate and severe	Unknown	Not mentioned	4.5 months	36/41 Matched	LVEDV, LVESV, LVEDD, LVEF, LAVI, mitral E wave, E/e’, RVD, RAD, TAPSE, sPAP, RV-GLS	HTN, T2DM, Smoking	RV systolic and diastolic impairment in post-COVID athletes compared to control group. (↓RV-GLS, ↓TAPSE, ↑RVD, ↑sPAP)
		Ozer et al. (2021) (2)	Home-recovered adults	Mild			4 months	43/41 Matched			
Uziębło-Życzkowska et al. (2022) Poland (41)	Observational cohort	None	Adults	Mild	Unknown	Fever, cough, myalgia, anosmia/ageusia, chest pain, dyspnea	3.5–4 months	31/28 Matched	LVGLS, LVEF, TAPSE, E/e', E/A	HTN, T2DM, hypothyroidism, COPD, Smoking	No significant finding
Wood et al. (2022) Denmark (42)	Retrospective cohort	None	Adults	Mixed (mild, moderate, severe)	Unknown	Chest pain, dyspnea, palpitation	13–15 month	22/22 Unknown	LVESD, LVEDD, LVESV, LVEDV, LVEF, IVSD, PWD, LAD, LAVI, E/e’, E/A, LV-GLS, TAPSE	None (excluded from the study)	LV diastolic impairment in post-COVID athletes compared to control group. (↓E/A, ↑E/e’)
Cotella et al. (2022) South America (43)	Cross-sectional	Cotella et al. (2022) (1)	Adults	Mild	Unknown	None	At least 14 days but less than months	46/25 Unknown	LV-GLS	None (Excluded from the study)	LV systolic impairment in post-COVID athletes compared to control group. (↓LV-GLS)
		Cotella et al. (2022) (2)		Moderate-Severe				30/25 Unknown			
Taş et al. (2023) Turkey (44)	Prospective cohort	None	Adults	Mild	Unknown	Palpitation, chest pain, fatigue, dyspnea, joint pain, cough, headache, insomnia	6 months	51/95 Matched	LVEDD, LVESD, LVEF, LVEF, LAVI, E wave, A wave, E/A, E/e’, RVD, RV-MPI, TAPSE	None (excluded from the study)	No significant finding
Hamdy et al. (2023) Egypt (45)	Cross-sectional	None	Adults	Not mentioned	Unknown	Dyspnea	Mean 3 Months (3 ± 1.7)	60/30 matched	LVEF, LVEDD, LVESD, LVEDV, LVESV, IVSD, PWD, LAD, LAVI, LVEF, E/A, E/e’	None (excluded from the study)	LV diastolic impairment in post-COVID patients compared to control group. (↑E/e’, ↑LAD, ↑LAVI)
Studies with only post-COVID cases (not included in the meta-analysis)											
Duration from acute COVID to Echo examination in recovery phase (<1 Month)											
Rasmusen et al. (2022) Denmark (29)	Prospective cohort	None	Young athletes	Not mentioned	None	None	2 weeks	121cases/ comparison of patients based on symptoms and duration of acute phase of COVID-19	LVEDD, LVEF, LVGLS, E/e’, TAPSE	Asthma (14%), electrical cardiac disease (2%)	No significant finding.
Duration from acute COVID to echo examination in recovery phase (1–3 Months)											
ZeinElabdeen et al. (2023) Egypt (46)	Prospective cohort	None	Adults	Not mentioned	Unknown	Asymptomatic, exertional dyspnea, fatigue, exercise intolerance (NYHA class ≥2)	1–3 months	63 cases/comparison of symptomatic patients and patients without any residual symptoms	LVESD, LVEDD, LVEF, LAVI, E wave, E/A, IVRT, LVGLS	None (excluded from the study)	LA strain and LA stiffness are early affected in patients with unexplained persistent dyspnea and exercise intolerance post- COVID-19, attributing to the impaired left ventricular diastolic function
ZeinELAbdeen et al. (2023) Egypt (47)	Cross-sectional	None	Adults	Mild and Moderate	1 dose (100%) 2 dose (90.4%) 3 dose (17.02%)	Palpitations (36.17%), dyspnea (26.6%), cough (22.34%), fatigue (27.65%), fever (3.19%), chest pain (7.44%)	1 month	94 cases/Comparison of post-COVID-19 patients with Postural orthostatic tachycardia and normal heart rate	LVEF, LVESD, LVEDD, LAD, LAVI, E/e'	None (Excluded from the study)	No significant finding
Sarıçam et al. (2021) Turkey (48)	cross-sectional	None	Adults	Not mentioned	Unknown	Palpitation, fatigue	3–8 weeks	105 cases/Comparison of asymptomatic patients with symptomatic patients	LVEF	None (Excluded from the study)	No significant finding
Tabacof et al. (2023) USA (49)	Retrospective observational cohort	None	Adults	Not mentioned (Not severe)	Unknown	Breathlessness, quality of life changes, fatigue, physical activity changes, cognitive function changes, anxiety, depression	1 month	203 cases/COVID-91 patients	LAD, RAD, LVEF, LVMI	Not mentioned	No significant finding
Samiei et al. (2023) Iran (50)	Cross-sectional	None	Adults	Mild, moderate, and severe	Unknown	Not mentioned	1.5 months	100 cases/Comparison according to the severity of symptoms defined by clinical features and lung CT	LVEF, LVGLS, E/e', RVEF, TAPSE, LAVI, LA peak strain, RA peak strain	None (Excluded from the study)	↓LV-GLS in patients with severe covid-19. Trend in reducing EF from 61% in milder groups to 55% in the severe group
Özer et al. (2021) Turkey (51)	Prospective cohort	None	Adults	Moderate and severe	Unknown	Not mentioned	1 month	74 cases/comparison of patients according to their hs-TnI levels at hospitalization	LVESDV, LVEDV, IVSD, PWD, LAD, LVEF, LVGLS	HTN (43.3%), T2DM (10.8%), smoking (8.1%)	↓LV-GLS in 1/3 patients recovered from COVID-19 infection.
Sollazzo et al. (2022) Italy (52)	Retrospective cohort	None	Athletes	Mild (98.6%), moderate (1.4%)	None (53%), First dose (39.2%), second dose (7.8%)	Chest pain (1.9%), palpitations (0.9%), shortness of breath (2.8%)	1 month	217 cases/Comparison of parameters assessed during pre-participation evaluation and return to play	RAD, LVEF, LVEDD, LVESD, IVSD, PWD, E/A	None (excluded from the study)	↓E/A ratio which commonly change according to the athletes’ training level.
Tudoran et al. (2021) (1) Romania (53)	Retrospective cohort	None	Adults	Mild and moderate	Unknown	Persistent fatigue, shortness of breath, chest discomfort/pain, palpitations, reduced effort capacity	1–3 months	150 cases/comparison of patients with and without Significant Cardiac Abnormalities due to covid-19 infection	LVMI, LAVI, E/A, E/e', TAPSE, RVGLS, LVGLS, LVEF	None (excluded from the study)	LV systolic and diastolic dysfunction was present in a subset of patients. (↓RV-GLS, ↓LVEF, ↑LAVI, ↓E/A, ↑E/e’)
Tudoran et al. (2021) (2) Romania (54)	Retrospective cohort	None	Adults	Mild and moderate	Unknown	fatigue, dyspnea, and palpitations	1.5–2.5 months	125 cases/Comparison of patients with diastolic dysfunction and normal cardiac function	LAVI, LVMI, LVEF, E/A, E/e', LVGLS, TAPSE, RVGLS	None (excluded from the study)	LV systolic and diastolic function were within normal limits, although we identified in 7 individuals mild LVH and another 4 patients had borderline values of RV-GLS.
Tudoran et al. (2021) (3) Romania (55)	Retrospective cohort	None	Adults	Mild and moderate	Unknown	Not mentioned	2 months	91 cases/COVID-91 patients	RAD, RVD, TAPSE, RVGLS	None (excluded from the study)	RV dysfunction were seen even after the recovery from mild Covid-19 pulmonary infections. (↓RV-GLS, ↑sPAP)
Tudoran et al. (2021) (4) Romania (56)	Prospective cohort	None	Adults	Mild and moderate	Unknown	Dyspnea, fatigability, palpitations, chest pain/discomfort, and reduced exercise tolerance	1 month 3 months 6 months	116 cases/Comparison of patients with and without pulmonary hypertension following covid-19 infection	LAVI, LVEF, RAD, RVD, TAPSE, RVGLS	None (excluded from the study)	49 cases had ↑ RVD and two borderline values of ↓TAPSE and/or ↓RV-GLS.
Tudoran et al. (2022) (1) Romania (57)	Prospective cohort	None	Adults	Mild and moderate	Unknown	Long-lasting fatigue, reduced exercise capacity, dyspnea, chest pain/discomfort, palpitations, increased blood pressure values, dizziness, concentration issues, foggy brain, and sleep disturbances	1 month 3 months 6 months	203 cases/comparison of patients according to their BMI and Metabolic syndrome history	LVMI, LVGLS, LVEF, LAVI, E/A, E/e', TAPSE, RVGLS	Metabolic syndrome	Severe forms of diastolic dysfunction were diagnosed, suggesting irreversible cardiac damages, such as interstitial fibrosis.
Tudoran et al. (2022) (2) Romania (58)	Prospective cohort	None	Adults	Mild and moderate	Unknown	Long-lasting fatigue, dyspnea, chest pain/discomfort, palpitations, and reduced exercise capacity	1 month 3 months 6 months	383 cases/Comparison of patients according to their type and severity of the prevailing cardiac dysfunction	LVMI, LAVI, LVEF, LVGLS, TAPSE, RVGLS, E/A, E/e', sPAP	None (excluded from the study)	LV systolic and diastolic impairment (↓LVEF, ↓LV-GLS, ↓ E/A ↑sPAP)
Tudoran et al. (2023) Romania (59)	Prospective cohort	None	Adults	Mild and moderate	Unknown	Reduced physical exertion capacity, persisting fatigue, palpitations, elevated blood pressure levels, chest discomfort or even pain, dyspnea, dry cough, sleep distur- bances, foggy brain, and concentration issues	1 month 3 months 6 months	203 cases/comparison of patients with and without T2DM and MS	LVMI, LAVI, LVEF, LVGLS, TAPSE, RVGLS, E/A, E/e'	T2DM, MS	RV, LV systolic and LV diastolic impairment in patients with MS and/or T2DM compared to healthy controls. (↑LVMI, ↑LAVI, ↓LVEF, ↓LV-GLS, ↓TAPSE, ↓RV-GLS, ↓E/A, ↑E/e')
Bende et al. (2021) Romania (60)	Retrospective cohort	None	Adults	Not mentioned	Unknown	Fatigue, shortness of breath, chest discomfort, palpitations, reduced exercise capacity	2–3 months	97 cases/comparison of patients with and without pulmonary injury	LVMI, LAVI, E/A, E/e', TAPSE, LVEF	HTN (23.7%), T2DM (4.12%),	LV systolic and diastolic impairment (↓LVEF, ↑E/e’) Only 3.09% of patients had ↓LVEF, and 31.95% ↑E/e’
Erdem et al. (2022) Turkey (61)	Retrospective cohort	None	Adults	Mild, moderate, and severe	Unknown	Exertional dyspnea (52.7%), palpitations (48.3%), chest pain (31.8%), and dyspnea at rest (15.3%), back pain (59.3%)	2–3 months	91 cases/Comparison of patients according to their hospitalization status and pulmonary involvement (ICU, covid-19 ward, outpatient)	RVD, TAPSE, LVEF, LAD, LVESD, LVEDD	HTN (21.9%), T2DM (14.2%), smoking (14.2%)	RV impairment in severe post-COVID cases. (↑RVD, ↓TAPSE)
Kujur et al. (2021) India (62)	Cross-sectional	None	Adults	Mild, moderate, and severe	Unknown	Not mentioned	1–3 months	100 cases/comparison of patients according to the disease severity	LVEF	HTN (27%), T2DM (24%), obesity (27%), CKD (4%)	Myocardial dysfunction is common in covid-19 regardless of disease severity. (↓LVEF)
Vera-Pineda et al. (2023) Mexico (63)	Cross-sectional	None	Adults	Mild (63%), moderate (15%), and severe (22%)	Unknown	Not mentioned (dyspnea, cough, palpitations, or fatigue)	At least 1.5 months	100 cases/comparison of patients according to the severity of the covid-19 infection and	LAD, LAVI, LVMI, TAPSE, LVEF, LV-GLS, RV-GLS	T2DM (22%), HTN (13%), dyslipidemia (8%), smoking (23%)	RV and LV systolic impairment in 70% cases (↑LAD, ↑LVMI, ↓TAPSE, ↓LVEF, ↓ LV-GLS, ↓RV-GLS)
Osada et al. (2022) USA (64)	Prospective cohort	None	Adults	Mild	50% One/both doses of SARS-CoV-2 Vaccine during the study (Three Moderna and one Pfizer)	Chest pain, chills, diarrhea, dizziness or vertigo, dry cough, dry eyes, dry mouth, fatigue, fever, headache, lack of appetite, anosmia, muscle or body aches, nasal congestion or runny nose, nausea or vomiting, shortness of breath, difficulty breathing, dyspnea, sore joints, or sore throat	1–6 months	18 cases/follow up from 1 to 6 months after COVID-19 infection	IVSD, LVM, LVMI, LVEF, LAD, E/e'	None (excluded from the study)	No significant finding
Can et al. (2024) Turkey (65)	Retrospective cohort	None	Adults	Mild and moderate	Unknown	Not mentioned	1 month 7 months	70 cases/Comparison of changes between the 1st and 7th month's follow up	LVEF, LVEDD, LVESD, IVSD, LAD, IVRT, E/A, E/e'	None (excluded from the study)	(↓LAD, ↑IVRT)
Duration from acute COVID to echo examination in recovery phase (≥3 Months)											
Yaroslavskaya et al. (2023) Russia (66)	Prospective cohort	None	Adults	Not mentioned	Unknown	Not mentioned	3 months, 12 months	156 cases/Comparison of patients with normal LVGLS and reduced LVGLS	LVGLS, LVEDV, LVESV, LVM, LVEF, IVRT, E/A, E/e', TAPSE	Not mentioned	LV systolic impairment 27.6% of patients after 1-year post-infection (↓LV-GLS)
Luchian et al. (2021) Belgium (67)	Prospective cohort	None	Adults	Moderate and sever	Unknown	Dyspnea (34.8%), Other symptoms were not mentioned.	12 months	66 cases/Comparison of patients with and without persistent dyspnea at the one-year follow-up	LVEF, E/A, E/e’, TAPSE	Dyslipidemia (19.7%) T2DM (16.7%) Obesity (25.8%) Smoking (12.1%) Cancer (3%) Chronic autoimmune disease (6.1%)	LV systolic impairment after 1-year post-infection (↓LV-GLS)
Matejova et al. (2022) Czechia (68)	Prospective observational cohort	None	Adults	Mild (74.4%) Moderate (4.7%) Severe (20.7%)	Unknown	Breathing problems, palpitations, exercise intolerance, fatigue in >50% of cases	3 months, 12 months	106 cases/COVID-19 patients	LVEF, LAD, LVEDD	HTN (20.2%) Obesity (11.5%) Bronchial asthma (5.8%) Renal insufficiency (5.8%) Depression (2.9%) Thromboembolic disease (1.9%)	No Significant finding.
Wu et al. (2021) China (69)	Prospective cohort	None	Adults	Mild (59.3%) Severe (40.7%)	Unknown	No cardiopulmonary symptoms.	6 months	27 cases/Comparison of patients with and without cardiac injury due to covid-19 infection	LAD, RAD, RVD, IVSD, LVEF, TAPSE	HTN (14.8%) T2DM (18.5%)	No Significant finding.
Stavileci et al. (2022) Turkey (70)	Retrospective cohort	None	Adults	Mild	Unknown	Cough (7.25%) Fever (19.35%) Joint Pain (30.64%) Fatigue (31.45%) Chest pain (9.67%) Dyspnea (20.96%) Taste abnormalities (29.83%) Smell abnormalities (26.1%)	6 months	248 cases/Comparison of patients with fragmented QRS (fQRS) wave and non- fragmented QRS wave	LVEF, LVESD, LVEDD, LAD, PWD, IVSD	Smoking (25%)	LVEF was statistically significant lower in the fQRS+ group compared to the non-fQRS group. Presence of fQRS was related also with wider: LVEDD, LVESD, septum thickness, and LAD
Sharma et al. (2022) India (71)	Prospective observational cohort	None	Adults	Mild, moderate, and severe	Unknown	Dyspnea (57.14%), Chest pain (19.04%), Palpitations (3.17%), Fatigue (25.39%)	6 months	63 cases/Comparison of patients with mild symptoms and moderate/severe symptoms	LVEF, E/A, E/e', TAPSE	HTN (28.57%), T2DM (14.28%)	LV dysfunction in moderate/severe group patients as compared to mild cases. No RV dysfunction. (↓LVEF, ↓E/A, ↑E/e')
Ro ´denas-Alesina et al. (2022) Spain (72)	Prospective cohort	None	Adults	Not mentioned	Unknown	Fever (89%), dyspnea (71.8%), diarrhea (24%)	4.3 months	109 cases/Comparison of patients with elevated biomarker (hs-TnI, NT-pro-BNP, D-dimer) with controls	LVEDD, LVMI, LVEF, TAPSE, LAVI, LVGLS, E/e'	Tobacco use, HTN, Dyslipidemia, T2DM, COPD, cancer,	Minimal changes were observed in LV function.
Chamtouri et al. (2022) Tunisia (77)	Prospective cohort	None	Adults	Not mentioned	Unknown	Not mentioned	3 months	111 cases/Comparison of patients according to CT scan lesions	LVEF, LVEDD, LVESD, IVSD, TAPSE, LVGLS, RVGLS	T2DM (31.8%), Hyperlipidemia (4.6%). HTN (45.8%), COPD (3.6%), smoking (7.8%)	This study showed that patients with severe CT scan pulmonary lesions were more likely to develop sub-clinical myocardial damage at mid-term follow-up. (↓LV-GLS, ↓RV-GLS)
Chudzik et al. (2022) Poland (73)	Prospective cohort	None	Adults	Mild, moderate, and severe	Unknown	Weakness (73%), impaired exercise tolerance (65.88%), palpitations (54%), memory and concentration disturbances (53.75%), chest pain (44%), Headache (34.39%), Dyspnea (32.02%), Excessive sweating (29.25%), Hair loss (28.06%), Muscle pain (24.6%), Anosmia and ageusia (24.12%), Cough (23.23%), Raynaud syndrome (14.29%), Ascites (swelling) (11.51%), Skin lesions (10.67%), Conjunctivitis (8.3%), Varicose veins of lower extremities (6.35%), Neurological disturbances (5.88%), Syncope (3.57%), Arthralgia (1.55%)	3 months	488 cases/Comparison of recovered patients with long covid-19 and no long covid-19	LAD, RVD, TAPSE, LVM, LVESD, LVEDD, IVSD	None (Excluded from the study)	Not significant finding
Flores et al. (2023) Braga (77)	Prospective cohort	None	Adults	Mild, moderate, and severe	Unknown	Not mentioned	6 months	88 cases/Comparison of patients admitted and not admitted to ICUs	LAD, LVEF, TAPSE	Not mentioned	Not significant finding
Kattamuri et al. (2023) India (74)	Prospective cohort	None	Adults	Mild and severe	Unknown	Not mentioned	3–6 months 6–12 months	53 cases/Comparison of patients with mild and severe covid-19	LVEF, E/A, E/e'	HTN (30%), T2DM (34%), Thyroid disease (3.7%)	No significant finding.
Hamburger et al. (2023) USA (75)	Prospective cohort	None	Athletes	Mild	Unknown	None	21 months	82 cases/Comparison of athletes pre- and post-training	LVEF, LAVI, LVEDD, LVESD, PWD, IVSD, LVMI, TAPSE, E/A, E/e'	None (Excluded from the study)	↑LVESD, ↑LAVI, ↑LVEDD
D’Ávila et al. (2023) Brazil (76)	Retrospective cohort	None	Adults	Moderate and severe	Unknown	Fatigue (71.4%), muscle pain (21.4%), Peripheral muscle weakness (19.6%), Dyspnea (17.9%)	7.9 months	56 cases/comparison of patients according to covid-19 severity	LVEDV, LVESV, LVEF, LVMI, TAPSE, LV-GLS, E/e'	HTN (63%), T2DM (20%), obesity (57%)	Despite having a similar EF and GLS, patients with a history of the critical manifestation in the acute phase of the disease had subclinical LV dysfunction according to other parameters. (↑global wasted work, ↓global work efficiency)

LVEDD, left ventricular end-diastolic diameter; LVESD, left ventricular end-systolic diameter; LVEDV, left ventricular end-diastolic volume; LVESV, left ventricular end-systolic volume; LVEF, left ventricular ejection fraction; PWD, posterior wall diameter; IVSD, interventricular septum diameter; LVM, left ventricular mass; LVMI, left ventricular mass index; LV-GLS, left ventricular global longitudinal strain; LAD, left atrium diameter; LAVI, left atrium volume index; LV-MPI, left ventricular myocardial performance index; E/A, the ratio of peak velocity blood flow from left ventricular relaxation in early diastole (the E wave) to peak velocity flow in late diastole caused by atrial contraction (the A wave); E/e’, ratio of E wave to early diastolic mitral annular velocity (e’); RVD, right ventricular diameter; RAD, right atrium diameter; RV-GLS, right ventricular global longitudinal strain; TAPSE, tricuspid annular plane systolic excursion; sPAP, systolic pulmonary artery pressure; RV-MPI, right ventricular myocardial performance index; MS, metabolic syndrome; T2DM, type 2 diabetes mellitus.

The severity of COVID-19 infection was not addressed in 12 studies (19, 29, 30, 32, 37, 45, 46, 48, 60, 66, 72, 77). Regarding the COVID-19 vaccination, only six studies (24, 29, 30, 46, 52, 64) provided information on the vaccination status of patients. It is noteworthy that data collection in most of the studies was conducted before the availability of any vaccines. Thirteen studies lacked information on post-COVID symptoms at the time of study enrollment (16, 20, 39, 40, 50, 51, 55, 62, 65, 66, 74, 77, 78). In most studies, the time interval between the acute phase of COVID-19 and echocardiography during the recovery phase was over 1 month, except for 3 studies (13, 16, 29) that were conducted within at least 10 days. Thirty studies reported the exclusion of patients with comorbid disease. On the other hand, three studies did not provide any information regarding the comorbid diseases (49, 66, 78). The primary focus of the studies pertained to the evaluation of LV function, with a secondary emphasis on RV function. A subset of studies also conducted concurrent assessments of both LV and RV function. Majority of the studies found significant changes in echocardiographic parameters, indicating subclinical alterations in the function of the LV and/or RV in post-COVID patients. However, 17 studies (17, 18, 27, 29, 30, 37, 41, 44, 46, 47, 49, 64, 68, 69, 73, 74, 78) did not report any significant findings.

### 3.3 Studies' risk of bias

**Figure 2:**
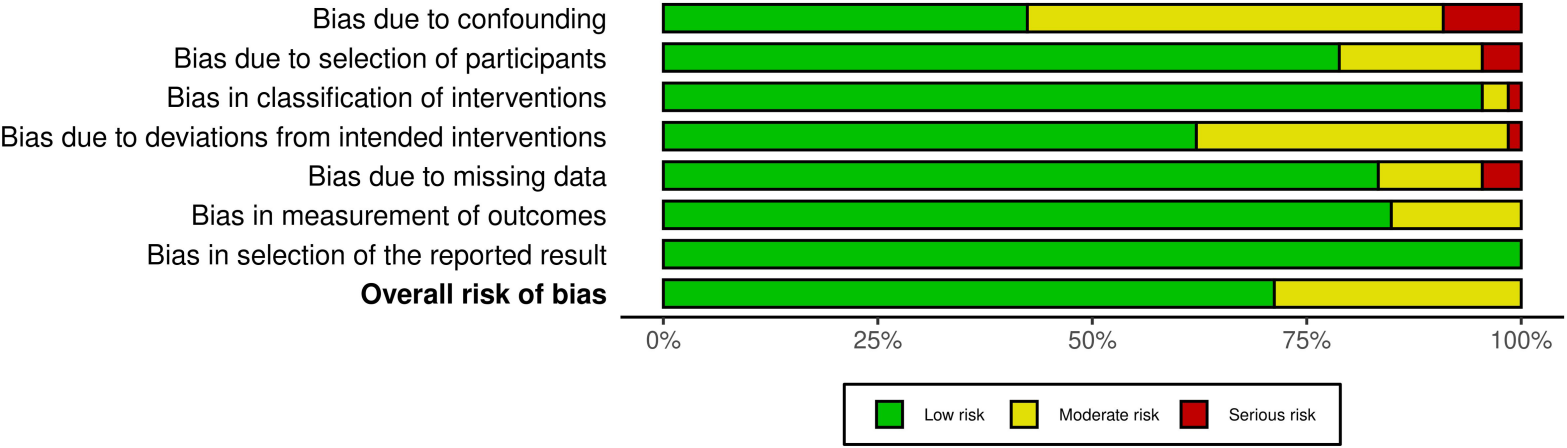
Overall risk of bias.

**Figure 3:**
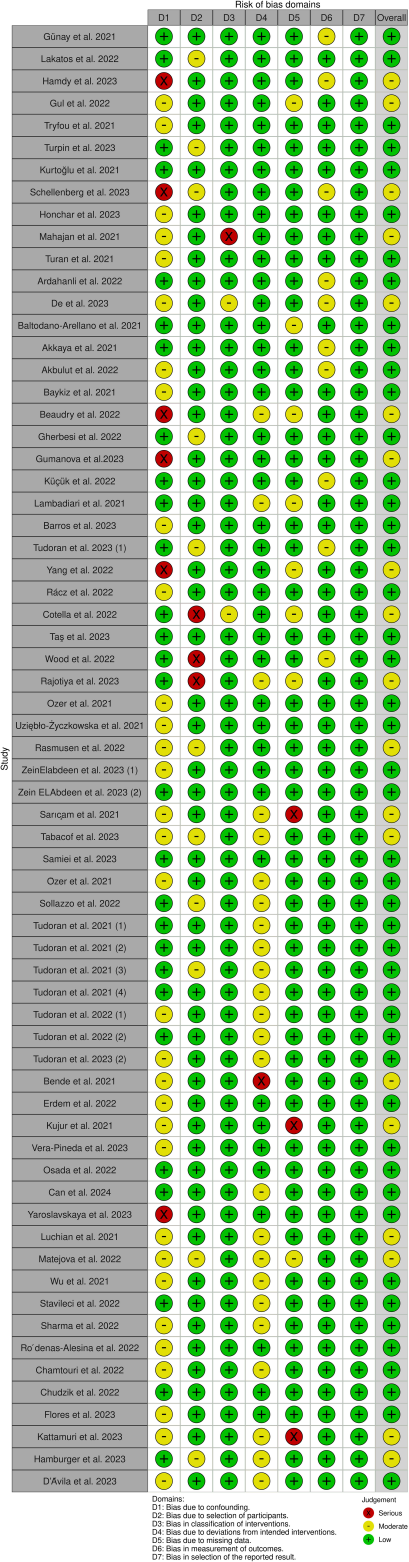
Risk-of-bias assessment (traffic light plot).

Figure 2 depicts a summary of the RoB-1 assessment. The overall risk of bias was found to be low to moderate. A low percentage (<15%) of serious risk of bias was identified in various domains, including confounding, selection of participants, classification of interventions, deviations from intended interventions, and missing data bias. Moderate risk of bias (25%–50%) was noted in confounding and deviations from intended interventions. There was no significant bias detected in the selection of reported results. Among 32 studies enrolled in the meta-analysis, five were found to have a serious risk of confounding bias (19, 30, 32, 37, 45). These studies did not provide information on the severity of COVID-19 infection in the patients. Additionally, 12 studies were rated as having a moderate risk of bias due to the presence of comorbid diseases that could impact heart function (15, 16, 20–22, 24, 27, 28, 35, 38, 40, 41). Three studies found a serious risk of bias in participant selection due to an unmatched case-control group (39, 42, 43), while five studies indicated a moderate risk due to the inclusion of specific populations such as athletes, women, and young adults that may not accurately represent the general population (13, 17, 19, 31, 36). Concerning bias due to classification of interventions, one study (21) found a serious risk of bias in comparing echo findings between two groups with reduced and normal-LVGLS, while two (24, 43) were deemed to have moderate risk due to incorrectly classifying post-COVID patients and comparing echo measures between them instead of with controls. Three studies demonstrated a moderate risk of bias due to deviations from intended interventions (30, 34, 39). Their main focus was on evaluating cardiopulmonary function rather than cardiac alone. Seven studies were found to have a moderate risk of bias due to missing data, and they reported small amounts of echocardiographic indices (15, 25, 30, 34, 37, 39, 43). Regarding bias in outcome measurement, 10 studies (14, 19, 22, 24, 26, 27, 33, 36, 42, 45) found a moderate risk of bias in reporting certain echo indices that deviated from the ranges reported in other studies (detailed in Table 2). Thirty-four studies only had post-COVID cases. Regarding the confounding factors, one study had a serious risk of bias as it did not provide information about the comorbid diseases of the patients (66). Twenty studies had moderate risks due to the presence of comorbid diseases (29, 51, 57, 59, 61–63, 67–69, 71, 72, 74, 76, 77) and lack of information on the severity of COVID-19 infection (29, 46, 48, 49, 72, 77). Considering the risk of bias in participant selection, 6 studies had moderate risks for reasons of inclusion of specific populations such as athletes (29, 52, 75) and having no classification and comparison among patients (49, 55, 68). Regarding bias due to deviations from intended interventions, one study had a serious risk of bias as its main focus was on hepatic abnormalities rather than cardiac alone (60). Twenty-one studies revealed moderate risk due to laboratory and biomarker evaluations, electrocardiogram evaluations, various surveys and lifestyle changes, return to play evaluation of athletes, chest computer tomography, post-COVID-19 functional status scale, cardio-ankle vascular index, ankle-brachial index, myocardial work analysis, walk test, pulmonary function tests, and cardiopulmonary exercise tests (48, 49, 51–59, 65, 67–71, 74–77). Bias due to missing data was serious in 3 studies as they reported small amounts of echocardiographic indices (48, 62, 74). Figure 3 represents the traffic light plot of risk of bias assessment for each included study.

**Table 2:** GRADE approach.

Outcome quality assessment										
Outcome	No. studies/methodology	Subgroups	Risk of bias	Limitation	Inconsistency	Indirectness	Imprecision	Other consideration	Mean difference (95% CI)	Certainty
LVESD										
Overall	15 6 prospective cohort 4 retrospective cohort 5 cross-sectional		Low	Low	Moderate^a^	Low	Moderate^b^	Reporting bias^c^	0.325 [−0.119, 0.352]	ꚚꚚꚚO Moderate
Grouped by duration		1–3 months	Low	Moderate^d^	High^e^	Low	Moderate^f^	None	−0.397 [−1.353, 0.560]	ꚚꚚꚚO Moderate
		≥3 months	Low	Low	Low	Low	Low	Reporting bias^c^	0.928 [0.566, 1.289]	ꚚꚚꚚꚚ High
Grouped by COVID-19 severity		Mild	Low	Low	Low	Low	Moderate^g^	None	0.908 [0.488, 1.32]	ꚚꚚꚚꚚ High ꚚꚚꚚO Moderate
		Moderate-Severe	Low	Moderate^4^	High^e^	Low	Moderate^f^	Reporting bias^c^	−0.272 [−1.42, 0.877]	
Grouped by comorbid disease		Present	Low	Low	Low	Low	Moderate^g^	None	−1.292 [−2.089, −0.495]	High ꚚꚚꚚO
		Absent	Low	Low	Low	Low	Low	Reporting bias^c^	0.905 [0.567, 1.24]	High ꚚꚚꚚO
LVESV										
Overall	6 2 cross-sectional 3 prospective cohort 1 case control	–	Low	Low	Low	Low	Moderate^f^	None	0.608 [−1.24, 2.45]	High ꚚꚚꚚO
Grouped by duration		≥3 months	Low	Moderate^d^	Moderate^a^	Low	Moderate^f^	None	1.69 [−1.95, 5.33]	ꚚꚚꚚO Moderate
Grouped by COVID-19 severity		Mild	High^h^	High^h^	Moderate^a^	Low	Moderate^f^	None	2.23 [−1.43, 5.88]	ꚚꚚOO Low
Grouped by comorbid disease		Present	High^h^	High^h^	Low	Low	Moderate^f^	None	5.55 [1.15, 9.96]	ꚚꚚOO Low
		Absent	Low	Moderate^d^	Low	Low	Moderate^f^	None	−0.451 [−2.48, 1.58]	ꚚꚚꚚO Moderate
LVEDD										
Overall	26 10 prospective cohort 3 retrospective cohort 1 Observational cohort 9 Cross-sectional 4 case control		Low	Low	Moderate^a^	Low	Moderate^b^	Reporting bias^c^	0.440 [−0.092, 0.155]	ꚚꚚꚚO Moderate
Grouped by duration		<1 month	High^h^	Moderate^d^	Low	Low	Moderate^f^	None	−0.232 [−1.29, 0.835]	ꚚꚚOO Low
		1–3 months	Low	Low	High^e^	Low	Moderate^b^	None	0.542 [−0.524, 1.608]	ꚚꚚꚚO Moderate
		≥3 months	Low	Low	Moderate^a^	Low	Moderate^b^	Reporting bias^c^	0.516 [−0.315, 1.346]	ꚚꚚꚚO Moderate
Grouped by COVID-19 severity		Mild	Low	Low	Moderate^a^	Low	Moderate^b^	Reporting bias^c^	0.580 [−0.199, 1.35]	ꚚꚚꚚO Moderate
		Moderate-Severe	Low	Low	High^e^	Low	Moderate^f^	None	0.620 [−0.421, 1.662]	ꚚꚚꚚO Moderate
Grouped by comorbid disease		Present	Low	Low	High^e^	Low	Moderate^c^	Reporting bias^c^	0.605 [−0.324, 1.533]	ꚚꚚꚚO Moderate
		Absent	Low	Low	Moderate^a^	Low	Moderate^c^	Reporting bias^c^	0.325 [−0.484, 1.133]	ꚚꚚꚚO Moderate
LVEDV										
Overall	11 4 Prospective Cohort 1 Retrospective Cohort 2 Cross-Sectional 4 Case-Control		High^i^	Low	Moderate^a^	Low	Moderate^e^	None	4.79 [−0.341, 9.93]	ꚚꚚꚚO Moderate
Grouped by duration		1–3 months	Low	Moderate^d^	Low	Low	Moderate^g^	None	6.87 [0.605, 13.13]	ꚚꚚꚚO Moderate
		≥3 months	Low^j^	Low	Moderate^a^	Low	Moderate^f^	Reporting bias^c^	3.88 [−2.35, 10.11]	ꚚꚚꚚO Moderate
Grouped by COVID-19 severity		Mild	Low	Moderate^d^	Low	Low	Moderate^g^	None	8.39 [3.57, 13.20]	ꚚꚚꚚO Moderate
		Moderate-Severe	Low	Moderate^d^	Low	Low	Moderate^g^	Reporting bias^d^	10.09 [2.29, 17.89]	ꚚꚚꚚO Moderate
Grouped by COVID-19 severity		Present	Low	Moderate^d^	Moderate^a^	Low	Moderate^g^	Reporting bias^k^	10.35 [4.93, 15.76]	ꚚꚚꚚO Moderate
		Absent	Low^j^	Low	Low	Low	Moderate^f^	None	0.602 [−4.87, 6.07]	ꚚꚚꚚO Moderate
IVSD										
Overall	19 6 Prospective Cohort 3 Retrospective Cohort 7 Cross-Sectional 4 Case-Control		Low^l^	Low	High^e^	Low	Moderate^b^	None	−0.203 [−0.526, 0.119]	ꚚꚚꚚO Moderate
Grouped by duration		<1 month	High^h^	High^h^	High^e^	Low	Moderate^f^	None	−0.108 [−1.16, 0.944]	ꚚOOO Very Low
		1–3 months	Low	Low	High^e^	Low	Moderate^b^	None	−0.108 [−1.16, 0.944]	ꚚꚚꚚO Moderate
		≥3 months	Low^l^	Low	Low	Low	Moderate^f^	Reporting bias^d^	−0.256 [−0.796, 0.284]	ꚚꚚOO Low
Grouped by COVID-19 severity		Mild	Low	Low	High^e^	Low	Moderate^b^	None	−0.411 [−0.830, 0.007]	ꚚꚚꚚO Moderate
		Moderate-Severe	High^m^	Moderate^d^	High^e^	Low	Moderate^f^	Reporting bias^d^	0.250 [−0.399, 0.899]	ꚚꚚOO Low
Grouped by comorbid disease		Present	High^m^	Moderate^d^	High^e^	Low	Moderate^f^	None	0.098 [−0.520, 0.715]	ꚚꚚꚚO Moderate
		Absent	High^m^	Low	High^e^	Low	Moderate^f^	None	−0.315 [−0.691, 0.061]	ꚚꚚOO Low
PWD										
Overall	19 6 Prospective Cohort 3 Retrospective Cohort 7 Cross-Sectional 3 Case-Control		Low	Low	High^e^	Low	Moderate^b^	None	0.086 [−0.139, 0.311]	ꚚꚚꚚO Moderate
Grouped by duration		<1 month	High^h^	High^h^	High^e^	Low	Moderate^f^	None	−0.127 [−0.762, 0.497]	ꚚꚚOO Low
		1–3 months	Low	Low	Low	Low	Moderate^f^	None	0.273 [−0.118, 0.663]	ꚚꚚꚚO Moderate
		≥3 months	Low	Low	High^f^	Low	Moderate^f^	None	−0.102 [−0.211, 0.007]	ꚚꚚꚚO Moderate
Grouped by COVID-19 severity		Mild	Low	Low	Moderate^a^	Low	Moderate^b^	None	−0.149 [−0.405, 0.106]	ꚚꚚꚚO Moderate
		Moderate-Severe	Low	Moderate^d^	Moderate^a^	Low	Moderate^g^	None	0.614 [0.259, 0.969]	ꚚꚚꚚO Moderate
Grouped by comorbid disease		Present	Low	Low	High^e^	Low	Moderate^f^	None	0.311 [−0.072, 0.695]	ꚚꚚꚚO Moderate
		Absent	Low	Low	Moderate^a^	Low	Moderate^b^	None	−0.006 [−0.248, 0.237]	ꚚꚚꚚO Moderate
LVM										
Overall	7 4 Prospective Cohort 2 Cross-Sectional 1 Case-Control		Low^n^	Low	Moderate^a^	Low	Moderate^f^	None	−7.630 [−21.7, 6.50]	ꚚꚚꚚO Moderate
Grouped by duration		1–3 months	Low^n^	Moderate^d^	High^e^	Low	Moderate^f^	None	−3.59 [−19.7, 12.53]	ꚚꚚꚚO Moderate
		≥3 months	Low^n^	Moderate^d^	Low	Low	Moderate^g^	None	−19.37 [−29.8, −8.92]	ꚚꚚꚚO Moderate
Grouped by COVID-19 severity		Mild	Low^n^	Moderate^d^	Low	Low	Moderate^g^	None	−13.71 [−25.30, −2.11]	ꚚꚚꚚO Moderate
		Moderate-Severe	Low	Moderate^d^	Low	Low	Moderate^g^	None	9.018 [0.458, 17.57]	ꚚꚚꚚO Moderate
Grouped by comorbid disease		Present	Low	Moderate^d^	Low	Low	Moderate^f^	None	7.54 [−0.720, 15.81]	ꚚꚚꚚO Moderate
		Absent	Low^n^	Moderate^d^	Low	Low	Moderate^g^	None	−18.28 [−26.72, −9.85]	ꚚꚚꚚO Moderate
LVMI										
Overall	7 1 Retrospective Cohort 1 Prospective Cohort 2 Cross-Sectional 2 Case-Control 1 Observational Cohort		Low^o^	Low	High^e^	Low	Moderate^b^	None	−1.65 [−6.62, 3.31]	ꚚꚚꚚO Moderate
Grouped by duration		1–3 months	Low^o^	Moderate^d^	Low	Low	Moderate^f^	None	−0.251 [−1.95, 1.45]	ꚚꚚꚚO Moderate ꚚꚚꚚO Moderate
		≥3 months	Low	Moderate^d^	High^e^	Low	Moderate^f^	None	−1.023 [−9.48, 7.44]	
Grouped by COVID-19 Severity		Mild	Low^o^	Moderate^d^	High^e^	Low	Moderate^f^	None	2.408 [−1.11, 5.93]	ꚚꚚꚚO Moderate
Grouped by comorbid disease		Absent	Low^o^	Low	High^e^	Low	Moderate^f^	None	−2.29 [−9.69, 5.11]	ꚚꚚꚚO Moderate
LVEF										
Overall	32 11 Prospective Cohort 4 Retrospective cohort 10 Cross-Sectional 7 Case-Control 1 Observational Cohort		High^p^	Low	Moderate^a^	Low	Low	Reporting bias^c^	−0.829 [−1.397, −0.262]	ꚚꚚOO Low
Grouped by duration		<1 month	High^h^	High^h^	High^e^	Low	Moderate^f^	None	0.667 [−1.42, 2.76]	ꚚꚚOO Low
		1–3 months	Low^q^	Low	High^e^	Low	Moderate^b^	None	−0.615 [−1.75, 0.527]	ꚚꚚꚚO Moderate
		≥3 months	Low^q^	Low	Moderate^a^	Low	Low	Reporting bias^c^	−1.16 [ −1.94, −0.375]	ꚚꚚꚚꚚ High
Grouped by COVID-19 severity		Mild	High^p^	Low	Moderate^a^	Low	Low	None	−0.886 [−1.64, −0.128]	ꚚꚚꚚO Moderate
		Moderate-Severe	Low	Low	Moderate^a^	Low	Moderate^f^	Reporting bias^c^	−0.900 [−1.96, 0.169]	ꚚꚚꚚO Moderate
Grouped by comorbid disease		Present	Low	Low	Moderate^a^	Low	Low	Reporting bias^c^	−0.852 [−1.66, −0.038]	ꚚꚚꚚꚚ High
		Absent	High^p^	Low	High^e^	Low	Low	None	−0.833 [−1.64, −0.005]	ꚚꚚOO Low
LV-GLS										
Overall	26 11 Prospective Cohort 2 Retrospective cohort 6 Cross-Sectional 6 Case-Control 1 Observational Cohort		Low^r^	Low	High^e^	Low	Low	None	1.21 [0.681, 1.75]	ꚚꚚꚚꚚ High
Grouped by duration		<1 month	Low	Moderate^d^	High^e^	Low	Moderate^f^	None	0.615 [−0.776, 2.00]	ꚚꚚꚚO Moderate
		1–3 months	Low	Low	Moderate^a^	Low	Low	None	1.22 [0.181, 2.27]	ꚚꚚꚚꚚ High
		≥3 months	Low^r^	Low	High^e^	Low	Low	None	1.37 [0.637, 2.11]	ꚚꚚꚚꚚ High
Grouped by COVID-19 severity		Mild	Low^r^	Low	High^e^	Low	Low	None	0.815 [0.047, 1.58]	ꚚꚚꚚꚚ High
		Moderate-Severe	Low^r^	Low	High^e^	Low	Moderate^g^	None	1.84 [0.751, 2.94]	ꚚꚚꚚꚚ High
Grouped by comorbid disease		Present	Low^r^	Low	High^e^	Low	Low	None	0.919 [0.07, 1.76]	ꚚꚚꚚꚚ High
		Absent	Low	Low	High^e^	Low	Low	None	1.43 [0.703, 2.16]	ꚚꚚꚚꚚ High
E/A ratio										
Overall	21 7 Prospective Cohort 3 Retrospective cohort 6 Cross-Sectional 4 Case-Control 1 Observational Cohort		High^s^	Low	High^e^	Low	Moderate^b^	None	−0.058 [−0.118, 0.002]	ꚚꚚOO Low
Grouped by duration		1–3 months	Low	Low	Low	Low	Low	None	−0.084 [−0.129, −0.039]	ꚚꚚꚚꚚ High
		≥3 months	High^s^	Low	High^e^	Low	Moderate^b^	None	−0.043 [−0.122, 0.035]	ꚚꚚOO Low
Grouped by COVID-19 severity		Mild	Low	Low	Moderate^a^	Low	Low	None	−0.042 [−0.076, −0.007]	ꚚꚚꚚꚚ High
		Moderate-Severe	Low	Moderate^d^	High^e^	Low	Moderate^g^	None	−0.134 [−0.258, −0.010]	ꚚꚚꚚꚚ High
Grouped by comorbid disease		Present	Low	Low	Moderate^a^	Low	Moderate^g^	None	−0.137 [−0.424, −0.032]	ꚚꚚOO Low
		Absent	Low^t^	Low	High^e^	Low	Moderate^b^	None	−0.021 [−0.093, 0.050]	ꚚꚚꚚꚚ High
E/e’ ratio										
Overall	27 9 Prospective Cohort 2 Retrospective cohort 9 Cross-Sectional 6 Case-Control 1 Observational Cohort		Low^u^	Low	High^e^	Low	Moderate^b^	None	0.116 [−0.275, 0.507]	ꚚꚚꚚO Moderate
Grouped by duration		<1 month	High^h^	High^h^	High^e^	Low	Moderate^f^	None	−0.412 [−1.79, 0.968]	ꚚꚚOO Low
		1–3 months	Low	Low	Moderate^a^	Low	Moderate^b^	None	−0.010 [−0.753, 0.732]	ꚚꚚꚚꚚ High
		≥3 months	Low^u^	Low	Moderate^a^	Low	Moderate^b^	Reporting bias^c^	0.315 [−0.213, 0.843]	ꚚꚚꚚO Moderate
Grouped by COVID-19 severity		Mild	Low	Low	High^e^	Low	Moderate^b^	None	0.105 [−0.410, 0.620]	ꚚꚚꚚO Moderate
		Moderate-Severe	Low	Low	Low	Low	Moderate^f^	Reporting bias^c^	0.380 [−0.009, 0.768]	ꚚꚚꚚO Moderate
Grouped by comorbid disease		Present	Low	Low	Low	Low	Moderate^b^	Reporting bias^c^	0.164 [−0.052, 0.381]	ꚚꚚꚚꚚ High
		Absent	Low^u^	Low	High^e^	Low	Moderate^b^	None	0.129 [−0.470, 0.729]	ꚚꚚꚚꚚ High
E wave										
Overall	13 4 Prospective Cohort 4 Cross-Sectional 5 Case-Control		Low	Low	Moderate^a^	Low	Moderate^b^	Reporting bias^c^	−0.013 [−0.029, 0.002]	ꚚꚚꚚO Moderate
Grouped by duration		1–3 months	Low	Low	Moderate^a^	Low	Moderate^b^	None	−0.021 [−0.052, 0.009]	ꚚꚚꚚꚚ High
		≥3 months	Low	Moderate^d^	Low	Low	Moderate^f^	Reporting bias^c^	0.009 [−0.025, 0.043]	ꚚꚚꚚO Moderate
Grouped by COVID-19 severity		Mild	Low	Low	Low	Low	Moderate^b^	None	0.001 [−0.017, 0.020]	ꚚꚚꚚꚚ High
		Moderate-Severe	Low	Moderate^d^	Low	Low	Moderate^g^	Reporting bias^c^	−0.052 [−0.082, −0.022]	ꚚꚚꚚO Moderate
Grouped by comorbid disease		Present	Low	Low	Moderate^a^	Low	Moderate^f^	Reporting bias^c^	−0.013 [−0.051, 0.026]	ꚚꚚꚚO Moderate
		Absent	Low	Low	Low	Low	Moderate^b^	None	−0.008 [−0.027, 0.011]	ꚚꚚꚚꚚ High
A wave										
Overall	10 4 Prospective Cohort 3 Cross-Sectional 3 Case-control		Low	Low	Moderate^a^	Low	Moderate^b^	Reporting bias^c^	0.026 [ −0.018, 0.070]	ꚚꚚꚚO Moderate
Grouped by duration		1–3 months	Low	Low	Low	Low	Moderate^g^	None	0.039 [0.017, 0.060]	ꚚꚚꚚꚚ High
		≥3 months	Low	Moderate^d^	Low	Low	Moderate^f^	None	0.034 [−0.034, 0.102]	ꚚꚚꚚO Moderate
Grouped by COVID-19 severity		Mild	Low	Low	High^e^	Low	Moderate^b^	None	−0.005 [−0.061, 0.050]	ꚚꚚꚚꚚ High
		Moderate-Severe	Low	Moderate^d^	Low	Low	Moderate^f^	None	0.086 [0.022, 0.150]	ꚚꚚꚚO Moderate
Grouped by comorbid disease		Present	Low	Moderate^d^	Low	Low	Moderate^f^	None	0.035 [−0.036, 0.105]	ꚚꚚꚚO Moderate
		Absent	Low	Low	High^e^	Low	Moderate^b^	None	0.014 [−0.051, 0.080]	ꚚꚚꚚꚚ High
LAD										
Overall	12 4 Prospective Cohort 1 Retrospective cohort 3 Cross-Sectional 4 Case-Control		Low	Low	High^e^	Low	Low	Reporting bias^c^	1.603 [0.696, 2.511]	ꚚꚚꚚꚚ High
Grouped by duration		1–3 months	Low	Moderate^d^	Moderate^a^	Low	Moderate^f^	None	1.127 [−0.571, 2.826]	ꚚꚚꚚO Moderate
		≥3 months	Low	Low	High^e^	Low	Moderate^g^	Reporting bias^c^	1.863 [0.694, 3.032]	ꚚꚚꚚꚚ High
Grouped by COVID-19 severity		Mild	Low	Moderate^d^	Moderate^a^	Low	Moderate^f^	Reporting bias^c^	0.937 [−0.183, 2.057]	ꚚꚚꚚO Moderate
		Moderate-Severe	Low	Moderate^d^	Moderate^a^	Low	Moderate^g^	Reporting bias^c^	2.305 [1.058, 3.74]	ꚚꚚꚚO Moderate
Grouped by comorbid disease		Present	Low	Moderate^d^	High^e^	Low	Moderate^g^	None	2.287 [0.910, 3.664]	ꚚꚚꚚO Moderate
		Absent	Low	Moderate^d^	High^e^	Low	Moderate^g^	Reporting bias^c^	1.064 [−0.167, 2.295]	ꚚꚚꚚO Moderate
LAVI										
Overall	15 4 Prospective Cohort 2 Retrospective cohort 4 Cross-Sectional 4 Case-Control 1 Observational Cohort		Low^c^	Low	High^e^	Low	Moderate^b^	Reporting bias^c^	0.895 [−0.509, 2.29]	ꚚꚚꚚO Moderate
Grouped by duration		1–3 months	High^h^	High^h^	Low	Low	Moderate^g^	None	1.95 [0.728, 3.17]	ꚚꚚOO Low
		≥3 months	Low^t^	Low	High^e^	Low	Moderate^f^	Reporting bias^c^	0.941 [−0.712, 2.59]	ꚚꚚꚚO Moderate
Grouped by COVID-19 severity		Mild	Low	Low	Moderate^1^	Low	Moderate^g^	None	0.922 [0.139, 1.845]	ꚚꚚꚚꚚ High
		Moderate-Severe	Low	Moderate^d^	Low	Low	Moderate^g^	Reporting bias^c^	1.475 [0.374, 2.575]	ꚚꚚꚚO Moderate
Grouped by comorbid disease		Present	Low	Low	Low	Low	Moderate^g^	Reporting bias^c^	1.135 [0.290, 1.980]	ꚚꚚꚚꚚ High
		Absent	Low^t^	Low	High^e^	Low	Moderate^f^	None	1.078 [−1.032, 3.187]	ꚚꚚꚚꚚ High
RV-GLS										
Overall	16 5 Prospective Cohort 2 Retrospective cohort 1 Cross-Sectional 6 Case-Control 1 Observational Cohort		Low	Low	High^e^	Low	Low	None	2.179 [1.099, 3.260]	ꚚꚚꚚꚚHigh
Grouped by duration		1–3 months	Low	Moderate^d^	High^e^	Low	Moderate^f^	None	1.547 [−0.335, 3.430]	ꚚꚚꚚO Moderate
		≥3 months	Low	Low	High^e^	Low	Low	None	1.842 [0.853, 2.831]	ꚚꚚꚚꚚ High
Grouped by COVID-19 severity		Mild	Low	Low	High^e^	Low	Moderate^f^	None	1.27 [−0.283, 2.73]	ꚚꚚꚚO Moderate
		Moderate-Severe	Low	Moderate^d^	High^e^	Low	Moderate^g^	None	4.306 [2.398, 6.214]	ꚚꚚꚚO Moderate
Grouped by comorbid disease		Present	Low	Low	High^e^	Low	Moderate^g^	None	2.228 [0.377, 4.079]	ꚚꚚꚚO Moderate
		Absent	Low	Low	High^e^	Low	Low	None	2.152 [0.807, 3.498]	ꚚꚚꚚꚚ High
RV-MPI										
Overall	6 1 Prospective Cohort 2 Cross-Sectional 3 Case-Control		Low	Low	High^e^	Low	Moderate^g^	None	0.060 [0.030, 0.089]	ꚚꚚꚚO Moderate
Grouped by duration		≥3 months	Low	Moderate^d^	High^e^	Low	Moderate^g^	None	0.035 [0.008, 0.062]	ꚚꚚOO Low
Grouped by COVID-19 severity		Mild	High^h^	High^h^	High^e^	Low	Moderate^g^	None	0.063 [0.021, 0.106]	ꚚꚚOO Low
Grouped by comorbid disease		Absent	Low^v^	Low	High^e^	Low	Moderate^g^	None	0.060 [0.030, 0.089]	ꚚꚚꚚO Moderate
RV diameter										
Overall	15 4 Prospective Cohort 2 Retrospective cohort 5 Cross-Sectional 4 Case-Control		High^w^	Low	High^e^	Low	Moderate^b^	Reporting bias^c^	0.306 [−0.566, 1.178]	ꚚꚚOO Low
Grouped by duration		1–3 months	High^w^	Moderate^e^	High^e^	Low	Moderate^g^	None	−1.820 [−3.406, −0.234]	ꚚꚚOO Low
		≥3 months	Low	Low	Low	Low	Low	Reporting bias^c^	0.900 [0.510, 1.290]	ꚚꚚꚚꚚ High
Grouped by COVID-19 severity		Mild	Low	Low	Low	Low	Low	Reporting bias^c^	0.865 [0.412, 1.317]	ꚚꚚꚚꚚ High
		Moderate-Severe	Low^v^	Low	High^e^	Low	Moderate^f^	None	−0.268 [−1.506, 0.970]	ꚚꚚꚚO Moderate
Grouped by comorbid disease		Present	Low	Low	Moderate^a^	Low	Moderate^f^	Reporting bias^c^	0.694 [0.242, 1.146]	ꚚꚚꚚO Moderate
		Absent	High^w^	Low	High^e^	Low	Moderate^b^	None	−0.227 [−1.467, 1.012]	ꚚꚚOO Low
TAPSE										
Overall	26 8 Prospective Cohort 3 Retrospective cohort 7 Cross-Sectional 8 Case-Control 1 Observational Cohort		Low	Low	High^e^	Low	Low	None	−1.01 [−1.621, −0.402]	ꚚꚚꚚꚚ High
Grouped by duration		1–3 months	Low	Low	High^e^	Low	Moderate^b^	None	−0.983 [−2.225, 0.285]	ꚚꚚꚚO Moderate
		≥3 months	Low	Low	High^e^	Low	Low	None	−1.160 [−1.885, −0.466]	ꚚꚚꚚꚚ High
Grouped by COVID-19 severity		Mild	Low	Low	Moderate^a^	Low	Moderate^f^	None	−0.283 [−1.050, 0.483]	ꚚꚚꚚꚚ High
		Moderate-Severe	Low	Low	Moderate^a^	Low	Moderate^g^	Reporting bias^c^	−1.234 [−2.197, −0.270]	ꚚꚚꚚO Moderate
Grouped by comorbid disease		Present	Low	Low	High^e^	Low	Moderate^b^	Reporting bias^c^	−0.510 [−1.409, 0.390]	ꚚꚚꚚO Moderate
		Absent	Low	Low	High^e^	Low	Low	None	−1.440 [−2.274, −0.608]	ꚚꚚꚚꚚ High
sPAP										
Overall	12 4 Prospective Cohort 1 Retrospective cohort 2 Cross-Sectional 5 Case-Control		Low	Low	High^e^	Low	Low	Reporting bias^c^	4.37 [2.378, 6.380]	ꚚꚚꚚꚚ High
Grouped by duration		≥3 months	Low	Low	High^e^	Low	Moderate^g^	Reporting bias^c^	5.172 [2.668, 7.676]	ꚚꚚꚚꚚ High
Grouped by COVID-19 severity		Mild	Low	Low	High^e^	Low	Low	Reporting bias^c^	3.749 [0.817, 6.682]	ꚚꚚꚚꚚO Moderate
		Moderate-Severe	Low^x^	Low	High^e^	Low	Moderate^g^	Reporting bias^c^	6.686 [3.109, 9.662]	ꚚꚚꚚꚚO Moderate
Grouped by comorbid disease		Present	Low^y^	Low	High^e^	Low	Low	Reporting bias^c^	6.777 [4.463, 9.091]	ꚚꚚꚚꚚ High
		Absent	Low	Low	High^e^	Low	Moderate^f^	Reporting bias^c^	2.039 [−0.181, 4.258]	ꚚꚚꚚꚚO Moderate

LVEDD, left ventricular end-diastolic diameter; LVESD, left ventricular end-systolic diameter; LVEDV, left ventricular end-diastolic volume; LVESV, left ventricular end-systolic volume; LVEF, eft ventricular ejection fraction; PWD, posterior wall diameter; IVSD, interventricular septum diameter; LVM, left ventricular mass; LVMI, left ventricular mass index; LV-GLS, left ventricular global longitudinal strain; LAD, left atrium diameter; LAVI, left atrium volume index; LV-MPI, left ventricular myocardial performance index; E/A, the ratio of peak velocity blood flow from left ventricular relaxation in early diastole (the E wave) to peak velocity flow in late diastole caused by atrial contraction (the A wave); E/e’, ratio of E wave to early diastolic mitral annular velocity (e’); RVD, right ventricular diameter; RAD, right atrium diameter; RV-GLS, right ventricular global longitudinal strain; TAPSE, tricuspid annular plane systolic excursion; sPAP, systolic pulmonary artery pressure; RV-MPI, right ventricular myocardial performance index; CI, confidence interval.

^a^ The level heterogeneity is moderate.

^b^ Confidence interval of the summary estimate included 0.

^c^ Majority of the studies are from Turkey.

^d^ The number of studies is less than 6.

^e^ The level heterogeneity is high.

^f^ The overall sample size was less than 500 and confidence interval of the summary estimate included 0.

^g^ The overall sample size was less than 500.

^h^ The number of studies is 2.

^i^ One of the include studies was at high risk of bias for this outcome and sensitivity analysis by excluding this study changed the statistical significance of the summary estimate to significant result (42).

^j^ One of the include studies was at high risk of bias for this outcome and sensitivity analysis by excluding this study did not change the direction or statistical significance of the summary estimate (42).

^k^ All of the studies are from Turkey.

^l^ Even though two of the included studies were at high risk of bias for this outcome, a sensitivity analysis by excluding this study did not change the magnitude, direction, or statistical significance of the summary estimate (23, 27).

^m^ Two of the include studies was at high risk of bias for this outcome and sensitivity analysis by excluding this study changed the statistical significance of the summary estimate to significant result (23, 27).

^n^ Two of the included studies evaluated athletes as their cases (17, 19) and excluding them, did not change the statistical significance of summary estimate.

^o^ One of the included studies evaluated athletes as their cases (17) and excluding them, did not change the statistical significance of summary estimate.

^p^ Three studies were at high risk of bias for LVEF and excluding them changed the significancy of the summary estimate to an insignificant result (17, 27, 36).

^q^ Three studies were at high risk of bias for LVEF and excluding them did not change the significancy of the summary estimate (17, 27, 36).

^r^ One of the include studies was at high risk of bias for this outcome and sensitivity analysis by excluding this study did not change the direction or statistical significance of the summary estimate (27).

^s^ One of the include studies was at high risk of bias for this outcome and sensitivity analysis by excluding this study changed the insignificancy of the summary estimate to a significant result (45).

^t^ One of the include studies was at high risk of bias for this outcome and sensitivity analysis by excluding this study did not change the insignificancy of the summary estimate (45).

^u^ Even though two of the included studies were at high risk of bias for this outcome, a sensitivity analysis by excluding this study did not change the magnitude, direction, or statistical significance of the summary estimate (42, 45).

^v^ One of the include studies was at high risk of bias for this outcome and sensitivity analysis by excluding this study did not change the significancy of the results (14).

^w^ One of the include studies was at high risk of bias for this outcome and sensitivity analysis by excluding this study changed the significancy of the results (14).

^x^ One of the include studies was at high risk of bias for this outcome and sensitivity analysis by excluding this study did not change the significancy of the results (33).

^y^ One of the include studies was at high risk of bias for this outcome and sensitivity analysis by excluding this study did not change the significancy of the result (24).

### 3.4 Outcome quality assessment

The certainty of evidence for outcomes, as assessed by GRADE framework, is delineated in Table 2. The meta-analysis indicates a moderate level of certainty in the majority of outcomes, primarily attributable to the inherent susceptibility to bias in observational studies. Outcomes with low certainty are typically caused by a small number of studies, significant heterogeneity, and the existence of potential biases.

### 3.5 Result of synthesis

#### 3.5.1 Overall outcomes

Among the echocardiographic measures of LV systolic function, LV-GLS and LVEF were found to be significantly different between the two groups being compared. The analysis of 26 studies showed a notable decrease in LV-GLS (less negative) in post-COVID patients (*n* = 1,810) compared to controls (*n* = 1,254), with a mean difference of 1.21 [95%CI (0.681, 1.75), *p* = 0.000, *I*^2^ = 91%]. Post-COVID patients (*n* = 2,173) exhibited a lower LVEF compared to controls (*n* = 1,770), with a MD of −0.829 [95%CI (−1.397, −0.262), *p* = 0.004, *I*^2^ = 73%]. Additionally, the meta-analysis of 12 studies revealed that LAD was significantly increased in post-COVID patients (*n* = 833) comparing to controls (*n* = 892) with a MD of 1.603 [95%CI (0.696, 2.511), *p* = 0.001, *I*^2^ = 80.7%]. However, LAVI was not significantly different comparing two groups with a MD of 0.895 [95% CI (−0.509, 2.29), *p* = 0.211, *I*^2^ = 82.7%]. In terms of RV evaluation, post-COVID patients showed significantly lower RV-GLS (less negative) and higher RV-MPI values compared to controls, with mean differences of 2.179 [95%CI (1.099, 3.260), *p* = 0.000, *I*^2^ = 85.4%] and 0.060 [95% CI (0.030, 0.089), *p* = 0.009, *I*^2^ = 99%], respectively. No significant differences were found in the diastolic and geometric indices of the left ventricle between the two groups being compared (Table 3). Forest plots are provided in supporting information (Supplementary S4 document).

**Table 3:** Result of synthesis.

	No. studies	No. cases	No. controls	Effect model	MD (CI: 95%)	*P* value	Heterogeneity	
							I2	*P* value
LV geometric indices								
LVEDD								
Overall	26	1,597	1,612	Random	0.440 [−0.092, 0.155]	0.148	65%	0.000
Grouped by duration from acute COVID to echo examination in recovery phase								
<1 months	2	174	144	Fixed	−0.232 [−1.29, 0.835]	0.670	0%	0.558
1–3 months	8	733	558	Random	0.542 [−0.524, 1.608]	0.319	80%	0.000
≥3 months	16	690	910	Random	0.516 [−0.315, 1.346]	0.223	58.6%	0.002
Grouped by severity of COVID-19 infection^a^								
Mild	15	1,079	1,205	Random	0.580 [−0.199, 1.35]	0.145	51.4%	0.011
Moderate-Sever	8	437	356	Random	0.620 [−0.421, 1.662]	0.243	78.8%	0.000
Mixed	2	81	91	Fixed	0.128 [−1.537, 1.792]	0.881	0%	0.955
Grouped by presence of comorbid diseases								
Present	12	645	542	Random	0.605 [−0.324, 1.533]	0.202	75.3%	0.000
Absent	14	952	1,070	Random	0.325 [−0.484, 1.133]	0.431	54%	0.008
LVEDV								
Overall	11	453	624	Random	4.79 [−0.341, 9.93]	0.067	56.9%	0.010
Grouped by duration from acute COVID to echo examination in recovery phase								
1–3 months	3	147	121	Fixed	6.87 [0.605, 13.13]	**0.032**	0%	0.536
≥3 months	8	306	503	Random	3.88 [−2.35, 10.11]	0.223	66%	0.004
Grouped by severity of COVID-19 infection^a^								
Mild	5	224	206	Fixed	8.39 [3.57, 13.20]	**0.001**	0%	0.753
Moderate-Sever	3	77	129	Fixed	10.09 [2.29, 17.89]	**0.011**	0%	0.728
Mixed	2	81	51	Random	−3.40 [−12.02, 5.22]	0.440	71.2%	0.062
Grouped by presence of comorbid diseases								
Present	5	154	214	Random	10.35 [4.93, 15.76]	**0.000**	62.6%	0.020
Absent	6	299	410	Fixed	0.602 [−4.87, 6.07]	0.829	0%	0.945
LVESD								
Overall	15	918	952	Random	0.325 [−0.119, 0.352]	0.346	73.7%	0.000
Grouped by duration from acute COVID to echo examination in recovery phase								
<1 months	1	67	37	–		–	–	–
1–3 months	4	346	217	Random	−0.397 [−1.353, 0.560]	0.417	80%	0.000
≥3 months	10	505	698	Fixed	0.928 [0.566, 1.289]	**0.000**	0%	0.541
Grouped by Severity of COVID-19 infection^a^								
Mild	7	407	437	Fixed	0.908 [0.488, 1.32]	**0.000**	32.1%	0.183
Moderate-Sever	5	360	227	Random	−0.272 [−1.42, 0.877]	0.642	84.5%	0.000
Mixed	2	81	51	Random	0.776 [−0.701, 2.254]	0.303	0%	0.772
Grouped by presence of comorbid diseases								
Present	5	334	202	Random	−1.292 [−2.089, −0.495]	**0.001**	54%	0.069
Absent	10	584	750	Fixed	0.905 [0.567, 1.24]	**0.000**	0%	0.570
LVESV								
Overall	6	317	431	Fixed	0.608 [−1.24, 2.45]	0.519	41.8%	0.127
Grouped by duration from acute COVID to echo examination in recovery phase								
1–3 months	1	86	60	–	0.500 [−2.77, 3.77]	–	–	–
≥3 months	5	231	371	Random	1.69 [−1.95, 5.33]	0.363	53%	0.0072
Grouped by Severity of COVID-19 infection^a^								
Mild	2	129	101	Random	2.23 [−1.43, 5.88]	0.230	57.7%	0.124
Moderate-Sever	1	36	41	–	6.20 [−2.17, 14.57]	–	–	–
Mixed	2	82	52	Fixed	1.40 [−3.08, 5.90]	0.539	0%	0.525
Grouped by presence of comorbid diseases								
Present	2	79	82	Fixed	5.55 [1.15, 9.96]	**0.013**	0%	0.859
Absent	4	238	349	Fixed	−0.451 [−2.48, 1.58]	0.664	0%	0.445
IVSD								
Overall	19	1,305	1,247	Random	−0.203 [−0.526, 0.119]	0.217	95.6%	0.000
Grouped by duration from acute COVID to echo examination in recovery phase								
<1 months	2	174	144	Random	−0.108 [−1.16, 0.944]	0.840	92.8%	0.000
1–3 months	8	677	530	Random	−0.256 [−0.796, 0.284]	0.352	98.2%	0.000
≥3 months	9	454	573	Fixed	−0.132 [−0.258, −0.007]	**0.039**	0%	0.456
Grouped by severity of COVID-19 infection^a^								
Mild	11	794	732	Random	−0.411 [−0.830, 0.007]	0.054	97.1%	0.000
Moderate-Sever	5	360	227	Random	0.250 [−0.399, 0.899]	0.451	80.4%	0.000
Mixed	2	81	51	Fixed	−0.128 [−0.427, 0.172]	0.403	0%	0.633
Grouped by presence of comorbid diseases								
Present	6	404	272	Random	0.098 [−0.520, 0.715]	0.756	88.4%	0.000
Absent	13	901	975	Random	−0.315 [−0.691, 0.061]	0.101	96.5%	0.000
PWD								
Overall	19	1,305	1,247		0.086 [−0.139, 0.311]	0.455	79.8%	0.000
Grouped by duration from Acute COVID to echo examination in recovery phase								
<1 months	2	174	144	Random	−0.127 [−0.762, 0.497]	0.690	96.3%	0.000
1–3 months	8	677	530	Random	0.273 [−0.118, 0.663]	0.171	0%	0.497
≥3 months	9	454	573	Fixed	−0.102 [−0.211, 0.007]	0.068	77.5%	0.000
Grouped by severity of COVID-19 infection^a^								
Mild	11	794	732	Random	−0.149 [−0.405, 0.106]	0.251	64.8%	0.002
Moderate-Sever	5	360	227	Random	0.614 [0.259, 0.969]	0.001	52.7%	0.076
Mixed	2	81	51	Fixed	−0.159 [−0.552, 0.233]	0.426	0%	0.496
Grouped by presence of comorbid diseases								
Present	6	404	272	Random	0.311 [−0.072, 0.695]	0.112	85.2%	0.000
Absent	13	901	975	Random	−0.006 [−0.248, 0.237]	0.962	63.7%	0.001
LVM								
Overall	7	453	478	Random	−7.630 [−21.7, 6.50]	0.290	74.8%	0.001
Grouped by duration from acute COVID to echo examination in recovery phase								
1–3 months	4	325	201	Random	−3.59 [−19.7, 12.53]	0.663	74.1%	0.009
≥3 months	3	128	277	Fixed	−19.37 [−29.8, −8.92]	**0.000**	0%	0.423
Grouped by Severity of COVID-19 infection^a^								
Mild	4	191	139	Fixed	−13.71 [−25.30, −2.11]	**0.020**	0%	0.426
Moderate-Sever	2	192	102	Fixed	9.018 [0.458, 17.57]	**0.039**	0%	0.845
Grouped by presence of comorbid diseases								
Present	3	234	128	Fixed	7.54 [−0.720, 15.81]	0.074	0%	0.420
Absent	4	219	350	Fixed	−18.28 [−26.72, −9.85]	**0.000**	19%	0.295
LVMI								
Overall	7	432	494	Random	−1.65 [−6.62, 3.31]	0.513	81.3%	0.000
Grouped by duration from acute COVID to echo examination in recovery phase								
1–3 months	3	237	149	Fixed	−0.251 [−1.95, 1.45]	0.772	0%	0.408
≥3 months	4	195	345	Random	−1.023 [−9.48, 7.44]	0.813	89.9%	0.000
Grouped by severity of COVID-19 infection^a^								
Mild	5	186	169	Random	2.408 [−1.11, 5.93]	0.181	73.1%	0.005
Moderate-Sever	1	176	88	–	0.00 [−1.74, 1.74]	–	–	–
Grouped by presence of comorbid diseases								
Present	1	176	88	–	0.00 [−1.74, 1.74]	–	–	–
Absent	6	256	406	Random	−2.29 [−9.69, 5.11]	0.544	83.6%	0.000
LV systolic function								
LVEF								
Overall	32	2,173	1,770	Random	−0.829 [−1.397, −0.262]	**0.004**	73.8%	0.000
Grouped by duration from acute COVID to echo examination in recovery phase^b^								
<1 months	2	174	144	Random	0.667 [−1.42, 2.76]	0.533	90.3%	0.001
1–3 months	9	691	498	Random	−0.615 [−1.75, 0.527]	0.291	83.5%	0.000
≥3 months	20	836	1,172	Random	−1.16 [ −1.94, −0.375]	**0.004**	60.1%	0.000
Grouped by severity of COVID-19 infection^a^								
Mild	20	1,586	1,109	Random	−0.886 [−1.64, −0.128]	**0.022**	78%	0.000
Moderate-Sever	10	495	402	Random	−0.900 [−1.96, 0.169]	0.098	69%	0.001
Mixed	1	22	22	–	−1.58 [−4.78, 1.62]	–	–	–
Grouped by presence of comorbid diseases								
Present	16	1,245	663	Random	−0.852 [−1.66, −0.038]	**0.040**	56.5%	0.002
Absent	16	928	1,107	Random	−0.833 [−1.64, −0.005]	**0.049**	79.6%	0.000
LV-GLS								
Overall	26	1,810	1,254	Random	1.21 [0.681, 1.75]	**0.000**	91%	0.000
Grouped by duration from acute COVID to echo examination in recovery phase								
<1 months	4	250	194	Random	0.615 [−0.776, 2.00]	0.386	95.9%	0.000
1–3 months	7	827	427	Random	1.22 [0.181, 2.27]	**0.021**	57.5%	0.028
≥3 months	15	733	633	Random	1.37 [0.637, 2.11]	**0.000**	92.9%	0.000
Grouped by severity of COVID-19 infection^a^								
Mild	13	1,084	714	Random	0.815 [0.047, 1.58]	**0.038**	88.1%	0.000
Moderate-Sever	7	380	308	Random	1.84 [0.751, 2.94]	**0.001**	90.1%	0.000
Mixed	3	226	112	Random	1.07 [−0.629, 2.77]	0.217	74.5%	0.020
Grouped by presence of comorbid diseases								
Present	12	884	552	Random	0.919 [0.07, 1.76]	**0.033**	88.8%	0.000
Absent	14	926	702	Random	1.43 [0.703, 2.16]	**0.000**	93.7%	0.000
LV diastolic function								
E/A ratio								
Overall	21	1,321	1,258		−0.058 [−0.118, 0.002]	0.057	79.3%	0.000
Grouped by duration from acute COVID to echo examination in recovery phase^b^								
<1 months	1	107	17	–	0.190 [−0.017, 0.397]	–	–	–
1–3 months	8	711	517	Fixed	−0.084 [−0.129, −0.039]	**0.000**	32%	0.167
≥3 months	12	503	724	Random	−0.043 [−0.122, 0.035]	0.280	85.6%	0.000
Grouped by severity of COVID-19 infection^a^								
Mild	13	835	706	Fixed	−0.042 [−0.076, −0.007]	**0.017**	45%	0.037
Moderate-Sever	5	334	263	Random	−0.134 [−0.258, −0.010]	**0.034**	82%	0.000
Mixed	2	82	52	Random	0.133 [−0.085, 0.351]	0.233	97%	0.000
Grouped by presence of comorbid diseases								
Present	7	382	322	Random	−0.137 [−0.424, −0.032]	**0.010**	57.9%	0.027
Absent	14	939	936	Random	−0.021 [−0.093, 0.050]	0.563	82%	0.000
E/e’ ration								
Overall	27	1,799	1,493		0.116 [−0.275, 0.507]	0.561	85.2%	0.000
Grouped by duration from acute COVID to echo examination in recovery phase^b^								
<1 months	2	174	144	Random	−0.412 [−1.79, 0.968]	0.558	80.7%	0.000
1–3 months	7	496	553	Random	−0.010 [−0.753, 0.732]	0.978	58.1	0.000
≥3 months	17	657	696	Random	0.315 [−0.213, 0.843]	0.243	87%	0.000
Grouped by Severity of COVID-19 infection^a^								
Mild	16	1,279	898	Random	0.105 [−0.410, 0.620]	0.689	82.6%	0.000
Moderate-Sever	6	336	274	Fixed	0.380 [−0.009, 0.768]	0.055	0%	0.808
Mixed	2	82	52	Random	0.379 [−1.15, 1.91]	0.629	98.5	0.000
Grouped by presence of comorbid diseases								
Present	16	1,179	706	Fixed	0.164 [−0.052, 0.381]	0.137	0%	0.521
Absent	11	620	787	Random	0.129 [−0.470, 0.729]	0.672	93.5%	0.000
Mitral E wave								
Overall	13	939	817	Fixed	−0.013 [−0.029, 0.002]	0.099	44%	0.040
Grouped by duration from acute COVID to echo examination in recovery phase								
<1 months	1	107	107		−0.010 [−0.059, 0.039]	**–**	**–**	
1–3 months	7	644	493	Random	−0.021 [−0.052, 0.009]	0.161	55.9%	0.043
≥3 months	5	188	217	Fixed	0.009 [−0.025, 0.043]	0.599	31.2%	0.213
Grouped by severity of COVID-19 infection								
Mild	9	660	636	Fixed	0.001 [−0.017, 0.020]	0.884	23.4%	0.234
Moderate-Sever	4	279	181	Fixed	−0.052 [−0.082, −0.022]	**0.001**	0%	0.437
Grouped by presence of comorbid diseases								
Present	6	383	280	Random	−0.013 [−0.051, 0.026]	0.527	62.1%	0.022
Absent	7	556	537	fixed	−0.008 [−0.027, 0.011]	0.426	21.1%	0.268
Mitral A wave								
Overall	10	684	647	Random	0.026 [ −0.018, 0.070]	0.245	72.8%	0.000
Grouped by duration from acute COVID to echo examination in recovery phase								
<1 months	1	107	107	–	−0.70 [−0.106, −0.034]	–	–	–
1–3 months	6	468	405	Fixed	0.039 [0.017, 0.060]	**0.000**	30.6%	0.206
≥3 months	3	109	135	Fixed	0.034 [−0.034, 0.102]	0.332	0%	0.912
Grouped by severity of COVID-19 infection								
Mild	7	566	500	Random	0.015 [−0.031, 0.062]	0.521	77.2%	0.000
Moderate-Sever	3	118	147	Fixed	0.086 [0.022, 0.150]	**0.008**	0%	0.575
Grouped by presence of comorbid diseases								
Present	3	128	110	Fixed	0.035 [−0.036, 0.105]	0.336	0%	0.913
Absent	7	556	537	Random	0.014 [−0.051, 0.080]	0.673	75%	0.001
Left atrium								
LAD								
Overall	12	833	892	Random	1.603 [0.696, 2.511]	**0.001**	80.7%	0.000
Grouped by duration from acute COVID to echo examination in recovery phase								
1–3 months	4	423	288	Random	1.127 [−0.571, 2.826]	0.193	50.8%	0.107
≥3 months	8	410	604	Random	1.863 [0.694, 3.032]	**0.002**	86.2%	0.000
Grouped by severity of COVID-19 infection^a^								
Mild	5	385	367	Random	0.937 [−0.183, 2.057]	0.101	63%	0.029
Moderate-Sever	5	318	258	Random	2.305 [1.058, 3.74]	**0.000**	63.7%	0.026
Mixed	1	60	30	Random	3.40 [1.850, 4.95]	–	–	–
Grouped by presence of comorbid diseases								
Present	6	447	388	Random	2.287 [0.910, 3.664]	**0.001**	77.4%	0.000
Absent	6	386	507	Random	1.064 [−0.167, 2.295]	0.090	83%	0.000
LAVI								
Overall	15	821	983	Random	0.895 [−0.509, 2.29]	0.211	82.7%	0.000
Grouped by duration from acute COVID to echo examination in recovery phase								
<1 months	1	107	107	–	−1.50 [−3.54, 0.543]	–	–	–
1–3 months	2	236	174	Fixed	1.95 [0.728, 3.17]	**0.002**	8.4%	0.296
≥3 months	12	478	702	Random	0.941 [−0.712, 2.59]	0.256	84%	0.000
Grouped by severity of COVID-19 infection^a^								
Mild	8	420	481	Fixed	0.922 [0.139, 1.845]	**0.023**	46%	0.073
Moderate-Sever	4	253	217	Fixed	1.475 [0.374, 2.575]	**0.009**	35.1%	0.201
Mixed	2	78	48	Random	3.06 [−0.174, 6.30]	0.064	93.8%	0.000
Grouped by presence of comorbid diseases								
Present	8	421	416	Fixed	1.135 [0.290, 1.980]	**0.008**	0%	0.462
Absent	7	400	567	Random	1.078 [−1.032, 3.187]	0.317	91.4%	0.000
Right heart function								
RV-GLS								
Overall	16	775	677	Random	2.179 [1.099, 3.260]	**0.000**	85.4%	0.000
Grouped by duration from acute COVID to echo examination in recovery phase								
<1 months	1	67	37	–	7.860 [6.33, 9.38]	–	–	–
1–3 months	3	170	129	Random	1.547 [−0.335, 3.430]	0.107	77.8%	0.011
≥3 months	12	538	511	Random	1.842 [0.853, 2.831]	**0.000**	74.3%	0.000
Grouped by severity of COVID-19 infection^a^								
Mild	7	380	331	Random	1.27 [−0.283, 2.73]	0.111	79.2%	0.000
Moderate-Sever	5	205	156	Random	4.306 [2.398, 6.214]	**0.000**	83.7%	0.000
Mixed	1	70	70	–	3.520 [2.61, 4.42]	–	–	–
Grouped by presence of comorbid diseases								
Present	7	268	224	Random	2.228 [0.377, 4.079]	**0.018**	90%	0.000
Absent	9	507	453	Random	2.152 [0.807, 3.498]	**0.002**	79%	0.000
RV-MPI								
Overall	6	327	352	Random	0.060 [0.030, 0.089]	**0.009**	99%	0.000
Grouped by duration from acute COVID to echo examination in recovery phase								
1–3 months	1	51	32	–	0.190 [0.166, 0.214]	–	–	–
≥3 months	5	276	320	Random	0.035 [0.008, 0.062]	**0.012**	98.9%	0.000
Grouped by severity of COVID-19 infection^a^								
Mild	2	156	200	Random	0.063 [0.021, 0.106]	**0.004**	93.8%	0.000
Moderate-Sever	1	51	32	–	0.190 [0.166, 0.214]	–	–	–
Grouped by presence of comorbid diseases								
Present	0	–	–	–	–	–	–	–
Absent	6	327	352	Random	0.060 [0.030, 0.089]	**0.009**	99%	0.000
RVD								
Overall	15	1,055	999	Random	0.306 [−0.566, 1.178]	0.492	85.5%	0.000
Grouped by duration from acute COVID to echo examination in recovery phase								
<1 months	1	107	107	–	0.600 [−0.539, 1.739]	–	–	–
1–3 months	3	283	180	Random	−1.820 [−3.406, −0.234]	**0.025**	93.4%	0.000
≥3 months	11	665	712	Fixed	0.900 [0.510, 1.290]	**0.000**	0%	0.703
Grouped by severity of COVID-19 infection								
Mild	8	666	674	Fixed	0.865 [0.412, 1.317]	**0.000**	0%	0.998
Moderate-Sever	7	389	325	Random	−0.268 [−1.506, 0.970]	0.672	92.6%	0.000
Grouped by presence of comorbid diseases								
Present	7	365	328	Fixed	0.694 [0.242, 1.146]	**0.003**	48%	0.001
Absent	8	690	671	Random	−0.227 [−1.467, 1.012]	0.719	90.8%	0.000
RAD								
Overall	8	450	422	Fixed	0.212 [−0.266, 0.689]	0.385	45.6%	0.075
Grouped by duration from acute COVID to echo examination in recovery phase								
1–3 months	2	246	158	Random	−0.329 [−1.710, 1.015]	0.640	70.5%	0.065
≥3 months	6	204	264	Fixed	0.499 [−0.096, 0.995]	0.107	21.4%	0.272
Grouped by severity of COVID-19 infection								
Mild	3	147	155	Fixed	0.695 [−0.237, 1.62]	0.144	0%	0.572
Moderate-Sever	5	309	267	Random	0.173 [−0.647, 0.994]	0.679	61.3%	0.035
Grouped by presence of comorbid diseases								
Present	7	400	372	Fixed	0.419 [−0.112, 0.951]	0.122	38.9%	0.132
Absent	1	50	50	–	−0.660 [−1.749, 0.429]	–	–	–
TAPSE								
Overall	26	1,458	1,381	Random	−1.01 [−1.621, −0.402]	**0.001**	82%	0.000
Grouped by duration from acute COVID to echo examination in recovery phase								
<1 months	1	107	107	–	1.100 [0.014, 2.186]	–	–	–
1–3 months	5	583	432	Random	−0.983 [−2.225, 0.285]	0.121	76.7%	0.002
≥3 months	20	768	842	Random	−1.160 [−1.885, −0.466]	**0.001**	80.3%	0.000
Grouped by severity of COVID-19 infection^a^								
Mild	11	809	792	Random	−0.283 [−1.050, 0.483]	0.469	70.3%	0.000
Moderate-Sever	8	405	345	Random	−1.234 [−2.197, −0.270]	**0.012**	57%	0.022
Mixed	2	92	92	Random	−3.564 [−5.727, −1.400]	**0.001**	67.6%	0.079
Grouped by presence of comorbid diseases								
Present	13	556	498	Random	−0.510 [−1.409, 0.390]	0.267	67.6%	0.000
Absent	13	902	883	Random	−1.440 [−2.274, −0.608]	**0.001**	87%	0.000
sPAP								
Overall	12	1,049	885	Random	4.37 [2.378, 6.380]	**0.000**	94.3%	0.000
Grouped by duration from acute COVID to echo examination in recovery phase^a^								
<1 months	1	107	107	–	0.300 [−0.979, 1.579]	–	–	–
1–3 months	1	51	32	–	5.70 [−2.010, 13.41]	–	–	–
≥3 months	9	419	646	Random	5.172 [2.668, 7.676]	**0.000**	95.2%	0.000
Grouped by severity of COVID-19 infection^a^								
Mild	6	801	437	Random	3.749 [0.817, 6.682]	**0.012**	92.3%	0.000
Moderate-Sever	5	178	211	Random	6.686 [3.109, 9.662]	**0.000**	95.2%	0.000
Grouped by presence of comorbid diseases								
Present	6	626	314	Random	6.777 [4.463, 9.091]	**0.000**	91.2%	0.000
Absent	6	423	571	Random	2.039 [−0.181, 4.258]	0.072	91%	0.000

LVEDD, left ventricular end-diastolic diameter; LVESD, left ventricular end-systolic diameter; LVEDV, left ventricular end-diastolic volume; LVESV, left ventricular end-systolic volume; LVEF, left ventricular ejection fraction; PWD, posterior wall diameter; IVSD, interventricular septum diameter; LVM, left ventricular mass; LVMI, left ventricular mass index; LV-GLS, eft ventricular global longitudinal strain; LAD, left atrium diameter; LAVI, left atrium volume index; LV-MPI, left ventricular myocardial performance index; E/A, the ratio of peak velocity blood flow from left ventricular relaxation in early diastole (the E wave) to peak velocity flow in late diastole caused by atrial contraction (the A wave); E/e’, ratio of E wave to early diastolic mitral annular velocity (e’); RVD, right ventricular diameter; RAD, right atrium diameter; RV-GLS, right ventricular global longitudinal strain; TAPSE, tricuspid annular plane systolic excursion; sPAP, systolic pulmonary artery pressure; RV-MPI, right ventricular myocardial performance index; CI, confidence interval.

Bold values show significant results (*p* < 0.05).

^a^ The studies conducted by Gumanova et al. (32), Beaudry et al. (30) and Yang et al. (37) did not report severity of COVID-19 infection.

^b^ One study did not report the timeframe from after recovering from COVID-19 to the echocardiography examination (24).

#### 3.5.2 Subgroup analysis

##### 3.5.2.1 Grouped by duration from acute COVID to echo examination in recovery phase

The subgroup meta-analysis found that among LV geometric indices, LVESD was notably higher in post-COVID patients (*n* = 505) compared to controls (*n* = 698) for a duration of ≥3 months, showing a MD of 0.928 [95% CI (0.566, 1.289), *p* = 0.000, *I*^2^ = 0%]. Moreover, post-COVID patients exhibited a significant decrease in IVSD and LVM compared to controls for a duration of ≥3 months, with a MD of −0.132 [95% CI (−0.258, −0.007), *p* = 0.039, *I*^2^ = 0%] and −19.37 [95%CI (−29.8, −8.92), *p* = 0.000, *I*^2^ = 0%], respectively.

In terms of systolic function lasting ≥3 months, LVEF was found to be significantly lower in post-COVID patients (*n* = 836) compared to controls (*n* = 1,172), with a MD of −1.16 [95% CI (−1.94, −0.375), *p* = 0.004, *I*^2^ = 60.1%]. Furthermore, post-COVID patients exhibited a significantly decreased LV-GLS (less negative) compared to controls for durations of both 1–3 months and ≥3 months, with MDs of 1.22 [95% CI (0.181, 2.27), *p* = 0.021, *I*^2^ = 57.5%] and 1.37 [95% CI (0.637, 2.11), *p* = 0.000, *I*^2^ = 92.9%], respectively.

There were significant differences in the E/A ratio and mitral A wave among diastolic function indices. Within a period of ≥3 months, post-COVID patients exhibited a significant reduction in the E/A ratio and an increase in the mitral A wave compared to the control group. The MDs were −0.084 [95%CI (−0.129, −0.039), *p* = 0.000, *I*^2^ = 32%] for the E/A ratio and 0.039 [95% CI (0.017, 0.060), *p* = 0.000, *I*^2^ = 30.6%] for the mitral A wave. Additionally, post-COVID patients (*n* = 410) exhibited a significant elevation in LAD in comparison to the control subjects (*n* = 604), over a duration of ≥3 months, with a MD of 1.863 [95% CI (0.694, 3.032), *p* = 0.002, *I*^2^ = 86.2%]. However, a meta-analysis of 2 studies showed a significant increase in LAVI in post-COVID patients (*n* = 236) compared to controls (*n* = 174) within a timeframe of 1–3 months, with a MD of 1.95 [95% CI (0.728, 3.17), *p* = 0.002, *I*^2^ = 8.4%].

In subgroup meta-analysis of RV function, RV-MPI, RVD and sPAP were significantly higher in post-COVID patients compared to control group for a duration of ≥3 months, with MDs of 0.035 [95% CI (0.008, 0.062), *p* = 0.012, *I*^2^ = 98.9], 0.900 [95% CI (0.510, 1.290), *p* = 0.000, *I*^2^ = 0%] and 5.172 [95%CI (2.668, 7.676), *p* = 0.000, *I*^2^ = 95.2%], respectively. Moreover, a significant decrease in TAPSE and RV-GLS (less negative) were observed in post-COVID patients compared to controls with a MD of −1.160 [95% CI (−1.885, −0.466), *p* = 0.001, *I*^2^ = 80.3%] and 1.842 [95%CI (0.853, 2.831), *p* = 0.000, *I*^2^ = 74.3%], respectively. Detailed information is provided in Table 3.

##### 3.5.2.2 Grouped by severity of COVID-19 infection

###### 3.5.2.2.1 Mild COVID-19 infection

In terms of mild COVID-19 infection and LV geometric indices, significant increase was observed in LVEDV and LVESD in post-COVID patients compared to controls with MDs of 8.39 [95% CI (3.57, 13.20), *p* = 0.001, *I*^2^ = 0%], and 0.908 [95% CI (0.488, 1.32), *p* = 0.000, *I*^2^ = 32.1%], respectively. LVM was significantly lower in post-COVID patients (*n* = 191) compared to controls (*n* = 139), with a MD of −13.71 [95 CI% (−25.30, −2.11), *p* = 0.020, *I*^2^ = 0%]. Moreover, significant changes in systolic function were observed in mild infection cases. Post-COVID patients reveled to have a decrease in LVEF and LV-GLS (less negative) compared to control groups, with MDs of −0.886 [95% CI (−1.64, −0.128), *p* = 0.022, *I*^2^ = 78%] and 0.815 [95% CI (0.047, 1.58), 0.038, *I*^2^ = 88.1%], respectively. Among LV diastolic indices, E/A ratio was significantly lower and LAVI was significantly increased in mild infection compared to controls, with MDs of −0.042 [95% CI (−0.076, −0.007), *p* = 0.017, *I*^2^ = 45%] and 0.922 [95% CI (0.139, 1.845), *p* = 0.023, *I*^2^ = 46%], respectively. RVD and sPAP were significantly higher in post-COVID patients compared to controls with MDs of 0.865 [95% CI (0.412, 1.317), *p* = 0.000, *I*^2^ = 0%] and 3.749 [95% CI (0.817, 6.682), *p* = 0.012, *I*^2^ = 0.012, *I*^2^ = 92.3%], respectively. Detailed information is provided in Table 3.

###### 3.5.2.2.2 Moderate and/or severe COVID-19 infection

Post-COVID patients exhibited higher values of LVEDV, PWD, and LVM compared to the control group. The MDs for LVEDV, PWD, and LVM were 10.09 [95% CI (2.29, 17.89), *p* = 0.011, *I*^2^ = 0%], 0.614 [95% CI (0.259, 0.969), *p* = 0.001, *I*^2^ = 52.7%], and 9.018 [95% CI (0.458, 17.57), *p* = 0.039, *I*^2^ = 0%], respectively. Concerning systolic function, there was no significant difference in LVEF between the two groups, as indicated by a MD of −0.900 [95% CI (−1.96, 0.169), *p* = 0.098, *I*^2^ = 69%]. Conversely, LV-GLS exhibited significantly lower (less negative) values in post-COVID patients in comparison to the control group, with a MD of 1.84 [95% CI (0.751, 2.94), *I*^2^ = 90.1%]. Among LV diastolic indices, E/A ratio and mitral E wave values were significantly decreased and mitral A wave was significantly increased in post-COVID patients compared to controls. The MDs for E/A, E wave and A wave were −0.134 [95% CI (−0.258, −0.010), *p* = 0.034, *I*^2^ = 82%], −0.052 [95% CI (−0.082, −0.022), *p* = 0.001, *I*^2^ = 0%] and 0.086 [95%CI (0.022, 0.150), *p* = 0.008, *I*^2^ = 0%], respectively.

Additionally, significantly higher values were found in both LAD and LAVI in post-COVID patients compared to controls. The MD for LAD was 2.305 [95% CI (1.058, 3.74), *p* = 0.000, *I*^2^ = 63.7%], and for LAVI it was 1.475 [95% CI (0.374, 2.575), *p* = 0.009, *I*^2^ = 32.1%].

Regarding RV indices, post-COVID patients showed significantly increased value in sPAP with MDs of 4.306 [95% CI (2.398, 6.214), *p* = 0.000, *I*^2^ = 83.7%]. Moreover, TAPSE and RV-GLS values were significantly lower in post-COVID patients compared to controls with MDs of −1.234 [95% CI (−2.197, −0.270), *p* = 0.012, *I*^2^ = 57%] and 6.686 [95% CI (3.109, 9.662), *p* = 0.000, *I*^2^ = 95.2%], respectively. Detailed information is provided in Table 3.

##### 3.5.2.3 Grouped by presence of comorbid diseases

Post-COVID patients with comorbidities showed higher values of LVEDV and LVESV compared to comorbid-matched control group with MDs of 10.35 [95% CI (4.93, 15.76), *p* = 0.000, *I*^2^ = 62.6%] and 5.55 [95% CI (1.15, 9.96), *p* = 0.013, *I*^2^ = 0%], respectively. There was a significant decrease in LVESD in post-COVID patients with comorbidities and an increase in cases without comorbidities compared to their comorbid-matched controls with MDs of −1.292 95% CI [−2.089, −0.495], *p* = 0.001, *I*^2^ = 43.2%) and 0.905 [95% CI (0.567, 1.24), *p* = 0.000, *I*^2^ = 0%], respectively. LVEF exhibited a significant decrease in post-COVID patients with comorbidities and those without comorbidities when compared to their comorbid-matched controls. The MDs were −0.852 [95% CI (−1.66, −0.038), *p* = 0.040, *I*^2^ = 56.5%] and −0.833 [95%CI (−1.64, −0.005), *p* = 0.049, *I*^2^ = 79.6%], respectively. Furthermore, LV-GLS was significantly decreased (less negative) in both groups of post-COVID patients, with MDs of 0.919 [95% CI (0.07, 1.76), *p* = 0.033, *I*^2^ = 88.8%] and 1.43 [95% CI (0.703, 2.16), *p* = 0.000, *I*^2^ = 93.7%] compared to their respective controls. E/A ratio was significantly lower in post-COVID patients with comorbidities compare to its comorbid-matched controls, with a MD of −0.137 [95% CI (−0.424, −0.032), *p* = 0.010, *I*^2^ = 57.9%]. Significantly higher values of both LAD and LAVI were observed in post-COVID patients with comorbidities compared to their matched controls. The MDs were 2.287 [95% CI (0.910, 3.664), *p* = 0.001, *I*^2^ = 77.4%] and 1.135 [95% CI (0.290, 1.980), *p* = 0.008, *I*^2^ = 0%], respectively.

Regarding RV function, RV-GLS was notably decreased (less negative) in both post-COVID patients with and without comorbidities compared to their controls, with MDs of 2.228 [95% CI (0.377, 4.079), *p* = 0.018, *I*2 = 90%] and 2.152 [95% CI (0.807, 3.498), *p* = 0.002, *I*^2^ = 79%]. Additionally, post-COVID patients without comorbidities presented higher values of RV-MPI with a MD of 0.060 [95% CI (0.030, 0.089), *p* = 0.009, *I*^2^ = 99%], compared to matched-controls. In post-COVID patients without comorbidities, TAPSE values were significantly lower, whereas no significant difference was found in cases with comorbidities when compared to their matched controls. The MDs were −1.440 [95%CI (−2.296, −0.585), *p* = 0.001, *I*^2^ = 87.4%] and −0.337 [95% CI (−1.213, 0.540), *p* = 0.452, *I*^2^ = 76%], respectively. Moreover, sPAP presented higher values in post-COVID patients with comorbidities and no significant result in cases without comorbidities compared to their matched controls with MDs of 6.777 [95% CI (4.463, 9.091), *p* = 0.000, *I*^2^ = 91.2%] and 2.039 [95%CI (−0.181, 4.258), *p* = 0.072, *I*^2^ = 91%], respectively. Detailed information is provided in Table 3. Forest plots are provided in supporting information (Supplementary S4 document).

Table 4 represent the summary of quantitative synthesis.

**Table 4:** Summary of quantitative synthesis.

Chamber function	Overall	Based on recovery phase	Based on severity of prior Covid-19 infection	Based on status of cardiovascular risk factors	Certainty of evidence
LV systolic function	Subclinical Impairment (↓LVEF, ↓LV-GLS)	• Long-Covid (≥3 months): ↓LVEF, ↓LV-GLS, ↓LVM, ↑LVESD, ↑LAD • Post-acute Covid (1–3 months): ↓LV-GLS	• Mild Infection: ↓LVEF, ↓LV-GLS, ↓LVM, ↑LVEDV • Moderate- Severe Infection: ↓LV-GLS, ↑LVM^a^	• Cardiovascular risk factors present: ↓LVEF, ↓LV-GLS, ↓LVESD, ↑LVESV • Cardiovascular risk factors absent: ↓LVEF, ↓LV-GLS, ↓LVM, ↑LVESD	Moderate ꚚꚚꚚO
LV diastolic function	Subclinical Impairment (↑LAD, ↓E/A)	• Long-Covid (≥3 months): ↑LAD, ↑IVSD, ↓E/A^a^ • Post-acute Covid (1–3 months): ↑LVEDV, ↓E/A, ↑A wave, ↑LAVI	• Mild Infection: ↑LVESD, ↓E/A • Moderate- Severe Infection: ↑LVEDV, ↑PWD, ↓E/A, ↓E wave, ↑A wave, ↑LAD	• Cardiovascular risk factors present: ↑LVEDV, ↓E/A, ↑LAD, ↑LAVI • Cardiovascular risk factors absent: None	Moderate ꚚꚚꚚO
RV systolic function	Subclinical Impairment (↓RV-GLS, ↑RV-MPI, ↓TAPSE)	• Long-Covid (≥3 months): ↓RV-GLS, ↑RV-MPI, ↑RVD, ↓TAPSE • Post-acute Covid (1–3 months)	• Mild infection: ↑RV-MPI, ↑RVD • Moderate- severe infection: ↓RV-GLS, ↓TAPSE	• Cardiovascular risk factors present: ↓RV-GLS, ↑RVD • Cardiovascular risk factors absent: ↓RV-GLS, ↑RV-MPI, ↓TAPSE	Moderate ꚚꚚꚚO
RV diastolic function	Subclinical Impairment (↑sPAP)	• Long-Covid (≥3 months): ↑sPAP • Post-acute Covid (1–3 months): ↓RVD	• Mild infection: ↑sPAP • Moderate- severe Infection: ↑sPAP	• Cardiovascular risk factors present: ↑sPAP • Cardiovascular risk factors absent: None	Moderate ꚚꚚꚚO

^a^ Significant results were obtained during sensitivity analysis.

### 3.6 Sensitivity analysis

#### 3.6.1 LVEDV

The study by Wood et al. (42), showed a high risk of bias for LVEDV in overall result of synthesis. Excluding this study revealed a significant difference between two groups of comparison with a MD of 4.732 [95% CI (1.367, 8.096), *p* = 0.006, *I*^2^ = 46.3%]. However, no significant difference was observed between two groups when grouped by duration ≥3 months and absence of comorbidities with MDs of 5.727 [95% CI (−0.209, 11.66), *p* = 0.059, *I*^2^ = 59.7%] and 1.964 [95% CI (−3.076, 7.00), 0.445, *I*^2^ = 57%], respectively.

#### 3.6.2 IVSD

The studies by Ardahanli et al. (23) and Akbulut et al. (27) were found to have a high risk of bias for IVSD for in overall result of synthesis and duration of ≥3 months. Excluding these studies did not change the direction, or statistical significance of the summary estimate with MDs 0.011 [95% CI (−0.147, 0.170), *p* = 0.891, *I*^2^ = 77%] and 0.135 [95%CI (−0.124, 0.394), *p* = 0.307, *I*^2^ = 88.5%], respectively. However, excluding these studies revealed significant difference between two groups of comparison in moderate-severe COVID-19 infection, presence and absence of comorbid disease with MDs of 0.539 [95%CI (0.281, 0.798), *p* = 0.000, *I*^2^ = 77%] and 0.320 [95%CI (0.019, 0.620), *p* = 0.037, *I*^2^ = 88.5%], −0.083 [95%CI (−0.143, −0.023), *p* = 0.007, *I*^2^ = 36%], respectively.

#### 3.6.3 LVM

Two studies (17, 19) were at high risk of bias for LVM due to involving athletes as their cases. A sensitivity analysis by excluding them did not change the direction, or statistical significance of the summary estimate with effect size of −5.78 [95%CI (−27.2, 15.3), *p* = 0.597, *I*^2^ = 83%].

#### 3.6.4 LVMI

The study by Turpin et al. (17) was deemed to have a high risk of bias for LVMI due to the inclusion of athletes as study participants. However, excluding this study did not change the direction, or statistical significance of the summary estimate with an effect size of −0.722 [95%CI (−6.575, 5.123), *p* = 0.809, *I*^2^ = 86.9%]. Furthermore, subgroup analyses focusing on mild COVID-19 infection and the absence of comorbid diseases also showed no change in the significance of the results when excluding this study. The effect sizes for mild COVID-19 infection and absence of comorbid diseases were 2.07 [95%CI (−7.21, 11.36), *p* = 0.622, *I*^2^ = 80.2%] and −1.06 [95%CI (−10.82, 8.70), *p* = 0.831, *I* ^2^ = 89%], respectively.

#### 3.6.5 LVEF

Three studies, conducted by Turpin et al. (17), Tudoran et al. (36) and Akbulut et al. (27), were deemed to have a high risk of bias in relation to LVEF. In a sensitivity analysis focusing on overall, mild COVID-19 and cases without comorbidities, the exclusion of these studies resulted in a change in the significance of the summary estimate. The effect size was found to be −0.499 [95% CI (−0.935, 0.037), *p* = 0.070, *I*^2^ = 63%] for overall cases, −0.229 [95% CI (−0.842, 0.383), *p* = 0.463, *I*^2^ = 62%] for mild cases, and −0.036 [95% CI (−0.686, 0.613), *p* = 0.913, *I*^2^ = 56.5%] for cases with absent comorbidities. However, excluding these studies did not change the direction, or statistical significance of the summary estimate for meta-analysis of duration ≥3 months with a MD of −0.693 [95% CI (−1.298, −0.087), *p* = 0.025, *I*^2^ = 47%].

#### 3.6.6 LV-GLS

Akkabulut et al. (27) was found to have a high risk of bias in the meta-analysis of LV-GLS for both overall results and durations of ≥3 months. Conducting a sensitivity analysis by excluding this study did not affect the significance of the results, with effect sizes of 1.43 [95%CI (0.900, 1.961), *p* = 0.000, *I*^2^ = 91%] and 1.78 [95%CI (1.049, 2.516), *p* = 0.000, *I*^2^ = 92%], respectively. Furthermore, excluding this study did not alter the significant findings in the subgroup analysis of severity of COVID-19 infection. The effect sizes remained significant at 1.021 [95%CI (0.265, 1.776), *p* = 0.008, *I*^2^ = 87%] for mild infection and 2.289 [95%CI (1.201, 2.314), *p* = 0.000, *I*^2^ = 89%] for moderate-severe infection.

#### 3.6.7 E/A ratio

Hamdy et al. (45) showed a high risk of bias in relation to this specific outcome. A sensitivity analysis was conducted by removing this study, changed the significancy of summary estimate for overall outcome and a duration of ≥3 months. The effect sizes were −0.079 [95%CI (−0.127, −0.032), *p* = 0.001, *I*^2^ = 64.6%] and −0.079 [95%CI (−0.141, −0.018), *p* = 0.011, *I*^2^ = 71.8%], respectively. However, excluding this study did not change the direction, or statistical significance of the summary estimate for the absence of comorbid diseases, with an effect size of −0.053 [95%CI (−0.109, 0.002), *p* = 0.061, *I*^2^ = 63.2%].

#### 3.6.8 E/e' ratio

Hamdy et al. (45) and Wood et al. (42) were found to have a high risk of bias regarding this outcome. Excluding these studies did not change the direction, or statistical significance of the summary estimate for the overall outcome and duration of ≥3 months. The effect sizes remained at 0.092 [95%CI (−0.229, 0.412), *p* = 0.575, *I*^2^ = 76.3%] and 0.333 [95%CI (−0.094, 0.759), *p* = 0.126, *I*^2^ = 72.4%] for each respective outcome.

#### 3.6.9 LAVI

Hamdy et al. (45) was found to have a high risk of bias for the outcome. Excluding this study did not change the direction, or statistical significance of the summary estimate for the overall outcome and duration of ≥3 months, with effect sizes of 0.578 [95%CI (−0.361, 1.517), *p* = 0.227, *I*^2^ = 51.4%] and 0.674 [95%CI (−0.077, 1.424), *p* = 0.079, *I*^2^ = 42.2%], respectively. The exclusion of this study also did not alter the lack of significance for the absence of comorbid disease, with an effect size of 0.214 [95%CI (−1.261, 1.688), *p* = 0.776, *I*^2^ = 73.4%].

#### 3.6.10 RV-MPI

The study by Günay et al. (14) had a high risk of bias for this particular outcome. A sensitivity analysis was conducted by removing this study did not change the significancy of summary estimate for the overall outcome, showing an effect size of 0.035 [95% CI (0.008, 0.062), *p* = 0.012, *I*^2^ = 98.9%].

#### 3.6.11 RVD

The study by Günay et al. (14) was found to have a high risk of bias for this particular outcome. Excluding this study changed the statistical significancy of summery estimates for the overall outcome, duration of 1–3 months, and the absence of comorbid disease, with effect sizes of 0.654 [95%CI (0.321, 0.987), *p* = 0.000, *I*^2^ = 17%], −0.277 [95%CI (−1.046, 0.493), *p* = 0.481, *I*^2^ = 21.3%] and 0.607 [95%CI (0.115, 1.099), *p* = 0.016, *I*^2^ = 0%], respectively. However, the sensitivity analysis for moderate-severe COVID-19 infection did not alter the direction or statistical significance of the summary estimate of the results. The effect sizes for these outcomes and 0.444 [95%CI (−0.099, 0.987), *p* = 0.109, *I*^2^ = 61.8%], respectively.

#### 3.6.12 sPAP

Küçük et al. (33) had a high risk of bias for moderate to severe COVID-19 infection. A sensitivity analysis that excluded this study showed that the result remained significant with an effect size of 8.016 [95%CI (6.800, 9.232), *p* = 0.000, *I*^2^ = 26.5%]. De et al. (24) was also at high risk of bias for the presence of comorbid disease. However, excluding this study in a sensitivity analysis did not change the direction or statistical significance of the summary estimate, with an effect size of 8.097 [95%CI (7.08, 9.113), *p* = 0.000, *I*^2^ = 0%].

Forest plots of sensitivity analysis are provided in supporting information (Supplementary S5 document).

### 3.7 Meta-regression

The results of the univariate meta-regression showed a significant positive correlation between MDs of RV-GLS and age. The effect size was 0.150 [95% CI (0.027, 0.272), *p* = 0.016, *R*^2^ = 0.32]. Moreover, MDs of TAPSE was negatively correlated with post-COVID patients' age with an effect size of −0.077 [95%CI (−0.152, −0.003), *p* = 0.04, *R*^2^ = 0.09]. There were no other significant correlations observed between echocardiographic variables and age or BMI. Detailed information is presented in Table 5. Scatter plots are provided in supporting information (Supplementary S6 document).

**Table 5:** Meta-regression results between echocardiographic indices and baseline characteristics of patients.

Moderator	No. studies	Coefficient	SE	Z value	*P* value	95% CI	*R* ^2^
E/A ratio							
Age	21	−0.004	0.003	−1.28	0.200	[−0.010, 0.002]	0.00
BMI	16	−0.004	0.017	0.23	0.815	[−0.038, 0.030]	0.00
LV-GLS							
Age	26	0.017	0.032	0.55	0.584	[−0.045, 0.080]	0.02
BMI	12	−0.062	0.162	−0.38	0.700	[−0.382, 0.256]	0.00
LAVI							
Age	15	−0.004	0.079	−0.05	0.960	[−0.158, 0.150]	0.00
BMI	13	−0.030	0.418	−0.07	0.941	[−0.850, 0.789]	0.00
LAD							
Age	13	−0.019	0.093	−0.21	0.833	[−0.202, 0.163]	0.00
BMI	11	0.465	0.301	1.54	0.122	[−0.125, 1.055]	0.00
RV-GLS							
Age	16	0.150	0.062	2.41	0.016	[0.027, 0.272]	0.32
BMI	6	−0.048	0.294	0.17	0.868	[−0.625, 0.528]	0.00
TAPSE							
Age	26	−0.077	0.041	−2.05	0.04	[−0.152, −0.003]	0.09
BMI	16	−0.133	0.129	−1.03	0.304	[−0.388, 0.121]	0.00
sPAP							
Age	12	0.162	0.105	1.55	0.122	[−0.043, 0.368]	0.00
BMI	11	0.730	0.502	1.45	0.145	[−0.253, 1.714]	0.00

### 3.8 Publication bias

A clear publication bias was observed when examining LVEF, LAVI, LAD and sPAP. After applying Duval and Tweedie's trim and fill method, it was determined that 9 studies needed to be added on the right side of the scatter plot for LVEF analysis. Following this adjustment, the effect size was calculated to be −0.120, with a 95%CI of (−0.711, 0.471). In the case of LAVI analysis, 5 studies needed to be imputed on the right side of the scatter plot, resulting in a summary effect size of 1.92, with a 95%CI of (0.689, 3.168). For LAD, 4 studies needed to be added on the left side of the scatter plot. The adjusted effect size was calculated 0.800 with a 95%CI of (−0.115, 1.716). Lastly, for sPAP correction analysis, 5 studies required imputation on the left side of the scatter plot, leading to a summary effect size of 1.29, with a 95%CI of (0.882, 1.717). Funnel plots and findings of Egger's and Begg's tests for all indices are provided in supporting information (Supplementary S7 document).

## 4 Discussion

In the present systematic review and meta-analysis, we performed a pooled analysis of 66 studies to evaluate the effect of SARS-CoV-2 infection on cardiac function in post-COVID-19 survivors without a prior history of cardiac issues or abnormalities. Following strict inclusion and exclusion criteria, we identified 32 studies that met the eligibility criteria for meta-analysis. This meta-analysis revealed significant myocardial alterations in individuals who have recovered from COVID-19 when compared to control groups. Furthermore, differences were observed in the function of the right and left ventricles in post-COVID patients compared to controls, especially in subgroup analyses based on the time since the onset of acute COVID-19 and echocardiogram evaluation during recovery, the severity of the initial infection, and the presence of comorbidities.

### 4.1 Definition

**“**Long COVID” or “post-COVID syndrome” is the term used to describe the ongoing presence of symptoms after a SARS-CoV-2 infection, lasting for weeks or months, regardless of whether the virus is still present in the body. These symptoms can persist or come back intermittently and may consist of either lingering symptoms from the initial COVID infection or new symptoms (79). The National Institute for Health and Care Excellence (NICE), the Scottish Intercollegiate Guidelines Network, and the Royal College of General Practitioners have collaborated to develop guidelines for individuals who have recuperated from COVID-19 but are still facing symptoms. They have coined the terms “post-acute COVID-19” for symptoms persisting 4–12 weeks after the initial infection and “long-COVID” for symptoms lasting beyond 12 weeks (80).

### 4.2 Echocardiographic evaluation of long COVID

The current systematic review and meta-analysis found that chronic COVID-19 patients exhibit impaired cardiac function in both the right and left sides of the heart. Unlike in previous reviews and meta-analyses, these patients did not have any history of cardiac disease and/or comorbidities that could affect their cardiac function.

#### 4.2.1 Left ventricular function

LV systolic dysfunction has been observed as a consequence of acute COVID-19 infection. Multiple studies have shown significant reductions in LVEF after 3 months of recovery from COVID-19, across a spectrum of symptoms and severity levels (24, 36, 39, 40). Additionally, there have been reports of reduced LVEF in chronic COVID-19 survivors, although these studies lacked a control group (70, 71, 75, 81).

LV-GLS provides valuable insight into LV function and is considered a more precise measure compared to LVEF (82). Long COVID patients, with and without a control group, were found to have reduced (less negative) LV-GLS (24, 25, 28, 31, 33, 34, 38, 66, 72, 77). However, there were reports of studies with no significant findings of LVEF and LV-GLS in long-COVID cases (26, 27, 37, 41, 74, 76, 78). The present meta-analysis revealed that individuals with long-COVID had significantly lower LV-GLS and LVEF compared to the control group. Unlike LVEF, decreased LV-GLS was also observed in COVID-19 patients with both mild and moderate-severe infections. Furthermore, reduced LVEF and LV-GLS were observed in COVID-19 patients with and without comorbidities compared to their matched groups.

Several studies have reported LV diastolic dysfunction in addition to LV systolic dysfunction. Long-COVID patients were found to have lower E/A and E/e' ratios compared to the control group (24, 28, 32, 36, 38, 40). In a study conducted by Sharma and colleagues (71), it was found that individuals with moderate to severe cases of COVID-19 had a greater likelihood of experiencing left ventricular diastolic dysfunction compared to those with mild cases when assessed through echocardiography six months post-infection (71). However, the present meta-analysis did not find any significant differences in E/e', E/A, mitral A wave, and mitral E wave between long-COVID patients and the control group.

Diastolic dysfunction is characterized by an irregular filling pattern in the left ventricle, often resulting in significant elevations in end-diastolic pressure during the filling of the ventricle (83). Left atrium enlargement is a key indicator of the structural remodeling process that occurs in reaction to chronically elevated LV end-diastolic pressure, typically resulting from diastolic dysfunction (83). In the current meta-analysis, it was found that LAD was significantly higher in long-COVID patients compared to the control group. However, there were no significant differences observed in LAVI between the two groups. In subgroup analysis, LAD and LAVI were increased in patients with history of moderate-severe COVID-19 infection and comorbid disease compared to their matched controls.

Additionally, abnormal LV shape can be a sign of both systolic and diastolic dysfunction. Several studies have shown that patients with long-lasting COVID-19 symptoms have significant alterations in LV geometric measurements (27, 32, 36, 38, 73, 75). In the current meta-analysis, it was observed that long-COVID patients exhibited lower LVM and IVSD compared to the control group, which could potentially suggest systolic dysfunction. Nevertheless, it is crucial to understand that lower LVM and IVSD levels may not necessarily signal systolic dysfunction. Instead, a decrease in LVM and IVSD may simply suggest a reduction in the size and thickness of the LV muscle. This decline could be attributed to factors like weight loss or reduced physical activity, which were not specifically examined in the present study (84).

Although long-COVID patients showed a decrease in LVM, further analysis by subgroup indicated an increase in LVM for those with moderate to severe COVID-19 infection and a decrease in LVM for those with mild infection. The pathophysiology of LV remodeling in the context of a SARS-CoV-2 infection is not fully understood, but it is likely related to the systemic inflammatory response triggered by the virus. It is suggested that COVID-19 can lead to a cytokine storm, where the immune system releases large amounts of pro-inflammatory cytokines in response to the infection. This excessive inflammation can damage the heart muscle and lead to LV hypertrophy over time (85). Moreover, these late pathological findings may be linked to the severity of the initial COVID-19 illness, the duration since the acute phase, and the presence of lingering symptoms (86).

#### 4.2.2 Right ventricular function

Research suggests that individuals may be at increased risk for right ventricular dysfunction after experiencing a severe case of COVID-19. This vulnerability is thought to be caused by the damage to the lungs and the rise in pulmonary vascular resistance resulting from the virus (87, 88). Several studies have demonstrated evidence of impaired RV function in individuals who have recovered from acute COVID-19, ranging from mild to severe infection, despite having no pre-existing cardiac conditions, for a duration exceeding 3 months comparing to control group (26, 28, 32, 34, 35, 40). However, studies conducted without a control group found that RV function was preserved in long-COVID cases (66, 69, 71, 73, 75). In the study conducted by Chamtouri et al., patients with severe pulmonary lesions detected on CT scans had a higher probability of experiencing subclinical myocardial injury during the mid-term monitoring period (77). In the current meta-analysis, long-COVID patients showed significantly impaired RV echocardiographic indices including increased RV diameter, sPAP, RV-MPI and reduced RV-GLS (less negative) and TAPSE compared to the control group. Pulmonary remodeling can lead to increased sPAP and RV-MPI. This can have a negative impact on the function of the right ventricle, leading to reductions in TAPSE and RV-GLS (89). In the subgroup analysis grouped by COVID-19 severity of infection, reduced RV-GLS and TAPSE were only observed in moderate-severe post-COVID patients compared to the control group. Earlier research indicates that even mild cases of COVID-19 may result in lasting cardiovascular complications (90). There is a possible relationship between the severity of inflammation during acute infection and long-term RV-GLS measurements (26). In addition, previous studies have demonstrated that assessing RV-GLS can be beneficial in predicting outcomes for patients with ARDS (91). It is believed that inflammation contributes to an increased workload and damage to the RV, ultimately leading to RV failure, which can be evaluated through RV-GLS (92).

In patients with COVID-19, it is crucial to recognize subclinical RV dysfunction, as reduced RV strain has been associated with increased mortality (93). This type of dysfunction appears to be common during post-recovery monitoring, even in individuals without preexisting cardiovascular or respiratory conditions or signs of heart failure, indicating potential unrecognized heart damage and compromised circulation following COVID-19 (94, 95).

### 4.3 Echocardiographic evaluation of post-acute COVID-19

#### 4.3.1 Left ventricular function

Among parameters indicating the LV systolic function, subgroup analysis showed impaired LV-GLS in post-COVID patients. Impaired LV-GLS was present in both mild infection and moderate-severe infections of COVID-19. Regarding the comorbidities, both patients with and without comorbid diseases had significant impairment in LV-GLS. These findings underscore the impact of COVID-19 on LVGLS, irrespective of disease severity or the presence of comorbidities. Samiei et al. (50) compared COVID-19 patients according to the severity of their infection. Reported LVGLS were in the normal range in all COVID-19 groups in their study (mild: −22.2 ± 2; moderate: −20.6 ± 2; severe: −19.3 ± 1). However, their investigation revealed that in the early recovery phase (1.5 months post-infection), LV-GLS was significantly lower in individuals who had suffered from a severe form of COVID-19 compared to others who experienced a milder clinical course. Özer et al. (51, 96) reported LVGLS below the normal range in the presence of COVID-19-caused myocardial injury (37) (−17.7 ± 2.6). They reported that myocardial LV-GLS values were impaired in one out of every three patients 1-month post-COVID-19 recovery. Tudoran et al. (58) conducted an assessment of the cardiac morphology and function in patients 1–3 months post-COVID-19 infection, analyzing and comparing the results based on the presence of cardiac abnormalities. They reported lower LV-GLS in 8.66% of patients with cardiac abnormalities. Since impaired LV-GLS shows subclinical myocardial deformation and is suggestive of LV-impaired systolic performance, post-acute COVID-19 patients are prone to the LV systolic dysfunction.

Moreover, the current meta-analysis on LV diastolic parameters found that the mitral A wave was significantly elevated and the E/A ratio was notably reduced when compared to the control group. This difference was both present in mild and moderate-severe COVID-19 infections. Furthermore, the difference was significant among patients with comorbid diseases. Sollazzo et al. (52) evaluated the cardiac function of athletes after mild or moderate COVID-19 infection. According to their findings, significant difference was observed in the E/A ratio, one month after the COVID-19 infection. Tudoran et al. (58) also reported an E/A ratio over 2, thus a type III diastolic dysfunction; and an E/A ratio of under 0.8, thus a type I diastolic dysfunction in a subset of their patients. According to these results, it can be concluded that COVID-19 causes impaired relaxation and consequently LV diastolic dysfunction in the early recovery phase.

Additionally, a notable disparity in LAVI was seen between post-acute COVID-19 patients and the control groups. The difference was seen in both mild infection and moderate-severe infection and the presence of comorbid diseases. Tudoran et al. (58) also reported increased LAVI in patients with cardiac abnormality 1–3 months after COVID-19 infection. COVID-19 infection might cause left atrial remodeling in the initial diastolic dysfunction phase by increased participation of left atrial active contraction to surpass the relaxation difficulty and thus, leading to A wave increase as well.

#### 4.3.2 Right ventricular function

In the present meta-analysis, only RV diameter showed a significant decrease in COVID-19 patients compared to the control groups. Erdem et al. (61) compared COVID-19 patients according to their hospitalization status and pulmonary involvement. Unlike the results of our meta-analysis, their findings showed that 2–3 months after recovering from COVID-19, RVD is increased in patients without a history of risk factors. Furthermore, the increases correlate with the severity of COVID-19 and the extent of pulmonary involvement. Tudoran et al. (55) compared patients with and without pulmonary hypertension following COVID-19 infection. As expected, RV diameter was significantly higher in patients with pulmonary hypertension. This controversy in the results might be due to the low number of studies included in our meta-analysis (5 studies). Although the occurrence of diastolic dysfunction prior to the systolic dysfunction might contribute to the decreased LV diameter.

### 4.4 A review of previous systematic reviews

Our search identified three systematic reviews (97–99) and two meta-analyses (100, 101) that investigated cardiac function in COVID-19 survivors.

In the meta-analysis conducted by Rahmati et al., a total of 21 studies were included (100). The inclusion criteria were studies that examined individuals who had recovered from COVID-19, in comparison to a control group, and presented findings of cardiac indices measured through Cardiac Magnetic Resonance (CMR), or echocardiography. They reported significant decrease in LVEDV, LVSV and LVEF in COVID-19 survivors compared to controls [(standardized mean difference (SMD) = −0.39, 95% CI = −0.56 to −0.22, *p* = 0.00001), (MD = −4.33, 95% CI = −5.72 to −2.94, *p* = 0.0000), and (SMD = −0.18, 95% CI = −0.34 to −0.01, *p* = 0.04), respectively]. No significant results were observed in LVEF across different post-COVID-19 follow-up periods in subgroup analysis. However, a decrease in LVEF was only evident where patients had a prior history of ICU admission grouped by the severity of COVID-19. LVM was significantly increased in COVID-19 cases in comparison to controls [(SMD = 0.23, 95%CI = 0.05–0.40, *p* = 0.01)]. Subgroup analysis showed that LVM started to increase significantly 3 months after recovery from COVID-19 infection. A meta-analysis of 5 studies showed a reduction of LV-GLS in recovered COVID-19 patients compared to controls (MD = −1.52, 95% CI = −1.64 to −0.97, *p* = 0.00001). Subgroup analysis revealed a decrease in LV-GLS would exist 2 months to 1 year after recovery. In terms of right heart indices, there was a significant reduction in RVEF, RVEDV, RVESV, RVSV and TAPSE of COVID-19 survivors compared to controls [(SMD = −0.29, 95% CI = −0.50 to −0.09, *p* = 0.005), (SMD = −0.42, 95% CI = −0.55 to −0.29, *p* = 0.00001), (SMD = −0.16, 95% CI = −0.29 to −0.03, *p* = 0.02), (MD = −0.50, 95% CI = −0.75 to −0.205, *p* = 0.0001) and (SMD = −0.91, 95% CI = −1.30 to −0.51, *p* = 0.00001), respectively] (100). Subgroup analysis revealed that TAPSE was reduced between 2 months and 1year post-COVID-19 recovery, while RVEF was reduced between 2 and 6 months after recovery. Subgroup analysis based on the severity of the acute COVID-19 phase and subsequent chronic outcome demonstrated a decrease in RVEF and RVESV only in patients who had been admitted to the ICU.

In the meta-analysis by Herold et al., they reviewed 32 CMR studies involving patients with COVID-19 that utilized myocardial longitudinal magnetization relaxation time constant (T1), transverse magnetization relaxation time constant (T2) mapping, extracellular volume, and late gadolinium enhancement (101). The authors suggested that T1 and T2 provided dynamic measures of cardiac involvement in COVID-19 survivors, indicating the improvement of cardiomyocyte injury and myocardial inflammation during recovery. In contrast, late gadolinium enhancement and, to a lesser extent, extracellular volume are seen as more static markers influenced by preexisting risk factors linked to adverse changes in myocardial tissue (101).

Ramadan et al. performed a comprehensive review to evaluate heart complications following recovery from COVID-19 (99). Of the studies analyzed, 12 employed CMR while 9 utilized echocardiography for cardiac function assessment. The median time for CMR evaluation was 63 days. The results indicated higher T1 intensity in 19% of cases, late gadolinium enhancement in 12% of cases, increased T2 intensity in 7% of cases, reduced GLS in 5% of cases, and decreased LVEF in 1.5%. In the echocardiography evaluation, the median time was 41 days. Reported outcomes included reduced LVEF, global hypokinesis, LV hypertrophy, diastolic dysfunction, pulmonary hypertension, and reduced GLS. Moreover, in the 3 to 6-month follow-up period, results showed a 30% decrease in LV-GLS, late gadolinium enhancement in 10% of cases on CMR, and diastolic dysfunction in 40% of cases on echocardiography (99).

Elhiny et al. conducted a review focusing on post-COVID-19 complications, including cardiovascular complications, in adults (97). Out of the studies analyzed, only three studies provided information on cardiac function assessment via imaging techniques. Two of these studies highlighted abnormal findings in CMR imaging. One study reported myocardial edema in 54% of COVID-19 survivors and positive late gadolinium enhancement in 31% of patients. Another study documented elevated myocardial native T1 and T2 values, myocardial, late gadolinium enhancement, pericardial enhancement, and reduced LVEF (less than 50%) in a subset of patients. Additionally, one study utilized trans-thoracic echocardiography to assess cardiac function, revealing a high prevalence of diastolic dysfunction, pulmonary hypertension, and pericardial effusion. Overall, this systematic review primarily focused on post-COVID-19 symptoms and complications across various organs, and the evaluation of cardiac function was found to be limited in scope.

Hassani et al. conducted a systematic review summarizing the CMR findings of COVID-19 adult survivors as reported in all available case series and cohort studies (98). Median follow-up time to MRI was at least 2 weeks after hospital discharge or diagnosis. The authors pooled data from 12 cohorts and 10 case series. Regarding the cardiac function, in 4 out of 8 cohort studies, RVEF was significantly lower than that in the control group. Mean/median LVEF fell in the normal range in all studies. However, six studies also reported the number of cases with LVEF <50%. One study found that RV and LV strains significantly decreased in COVID-19 with late gadolinium enhancement compared to those without late gadolinium enhancement and normal controls.

In contrast to earlier systematic reviews, we implemented measures to eliminate the possible interference of pre-existing heart conditions on the cardiac function of individuals who have recovered from COVID-19. Instead of combining echocardiography and CMR in the present meta-analysis like previous assessments, we focused solely on echocardiography to reduce variability and improve the accuracy of our results. Additionally, we conducted subgroup analysis to provide a more thorough understanding of the data. Moreover, we have incorporated a greater quantity of recent studies to validate the strength of our conclusions.

### 4.5 Limitations

It is important to acknowledge certain limitations when comparing the provided reports. To eliminate the heterogeneity of studies and the reported echocardiographic measurements, a subgroup meta-analysis was conducted. However, it is important to note that due to intrinsic limitations of the studies included, some degree of heterogeneity was inevitable. The parameters evaluated in echocardiography exhibited a wide range of variability, making it impossible to assess all the reported parameters. Additionally, there are several factors that may impact the function of the LV and RV, such as the vaccination status of patients and the treatment approach for COVID-19 infection during the acute phase. Unfortunately, the absence of data on these two variables in the research it was not feasible to compare the data.

### 4.6 Implications for research, practice and policy

Further research should focus on identifying risk factors for developing cardiac complications following COVID-19, as well as potential preventive measures and investigate potential treatments and interventions to prevent or manage cardiac dysfunction in COVID-19 survivors. Healthcare providers should be aware of the increased risk of cardiac dysfunction in individuals who have recovered from COVID-19, even if they did not experience severe symptoms during their initial illness. Regular cardiac monitoring and follow-up assessments should be considered for COVID-19 survivors, especially those with pre-existing cardiovascular conditions or other risk factors for heart disease. Policy makers should consider implementing guidelines for cardiac screening and follow-up care for COVID-19 survivors, to ensure early detection and appropriate management of cardiac dysfunction.

## 5 Conclusion

This systematic review discusses emerging research on the possible development of cardiac dysfunction following the resolution of acute COVID-19 infection. Findings revealed subclinical changes in both left and right ventricular systolic and diastolic function among post-acute and long COVID patients without a prior history of heart disease, including individuals lacking cardiovascular risk factors such as diabetes and hypertension, irrespective of the severity of their initial illness. While these changes remained within normal limits, they were markedly different from those in non-COVID control subjects, indicating potential underlying issues that warrant further exploration.

## Data Availability

The original data are presented in original full-texts of studies included in this review. Data-set are presented in forest plots/Supplementary Material, further inquiries can be directed to the corresponding author.

## Supplementary material

Supplementary S1 Document PRISMA 2020 Checklist for systematic reviews and meta-analyses.

Supplementary S2 Document Table of search strategy conducted in online databases.

Supplementary S3 Document Table of excluded studies during final screening.

Supplementary S4 Document Forest plots of meta-analysis.

Supplementary S5 Document Forest plots of sensitivity analysis.

Supplementary S6 Document Scatter plots of meta-regression.

Supplementary S7 Document Funnel plots of meta-analysis.
