# Supplementary material for: A deeper look at long-term effects of COVID-19 on myocardial function in survivors with no prior heart diseases: a GRADE approach systematic review and meta-analysis
Source: Front Cardiovasc Med. 2024 Nov 19;11:1458389. doi: 10.3389/fcvm.2024.1458389 (PMC11611865; doi:10.3389/fcvm.2024.1458389)
Supplement: Supplementary S2 Document — Table of search strategy conducted in online databases. [file Datasheet2.docx]

**Table of Search Strategy**

| **Databases** | **Search terms** |
| --- | --- |
| **Web of Science** | #1 TS=((("left ventric*" OR "right ventric*" OR "left cardiac*" OR "right heart" OR "right cardiac" OR "left heart" OR atri* OR myocardi* OR diastol* OR systol*) NEAR/1 (dysfunction OR function OR remodeling OR impair* OR hypertroph* OR active* OR volume OR mass* OR dimension* OR diameter OR thickness OR index* OR "ejection time" OR "ejection fraction")) OR Echocardiograph* OR Echo)  #3 TS=( "covid-19" OR "sars cov 2")  #4 (#1 OR #2) AND #3 |
| **PubMed** | #1 ((("left ventric*"[Title/Abstract] OR "right ventric*"[Title/Abstract] OR "left cardiac*"[Title/Abstract] OR "right heart"[Title/Abstract] OR "right cardiac"[Title/Abstract] OR "left heart"[Title/Abstract] OR atri*[Title/Abstract] OR myocardi*[Title/Abstract] OR diastol*[Title/Abstract] OR systol*[Title/Abstract]) AND (dysfunction[Title/Abstract] OR function[Title/Abstract] OR remodeling[Title/Abstract] OR impair*[Title/Abstract] OR hypertroph*[Title/Abstract] OR active*[Title/Abstract] OR volume[Title/Abstract] OR mass*[Title/Abstract] OR dimension*[Title/Abstract] OR diameter[Title/Abstract] OR thickness[Title/Abstract] OR index*[Title/Abstract] OR "ejection time"[Title/Abstract] OR "ejection fraction"[Title/Abstract])) OR Echocardiograph*[Title/Abstract] OR Echo[Title/Abstract])  #2 "COVID-19"[Mesh] OR "SARS-CoV-2"[Mesh]  #3 #1 AND #2 |
| **Scopus** | #1 TITLE-ABS-KEY ( ( ( "left ventric*" OR "right ventric*" OR "left cardiac*" OR "right heart" OR "right cardiac" OR "left heart" OR atri* OR myocardi* OR diastol* OR systol* ) PRE/1 ( dysfunction OR function OR remodeling OR impair* OR hypertroph* OR active* OR volume OR mass* OR dimension* OR diameter OR thickness OR index* OR "ejection time" OR "ejection fraction" ) ) OR echo OR echocardiograph* )  #2 TITLE-ABS-KEY ("covid-19" OR "sars cov 2")  #3 #1 AND #2 |
| **Cochrane** | #1 (((("left ventricular" OR "right ventricular" OR "left cardiac" OR "right heart" OR "right cardiac" OR "left heart" OR atrial OR myocard OR diastol OR systol) NEXT (dysfunction OR function OR remodeling OR impair* OR hypertrophy OR active OR volume OR mass OR dimension OR diameter OR thickness OR index OR "ejection time" OR "ejection fraction")) OR Echocardiography OR Echo)):ti,ab,kw  #2 MeSH descriptor: [COVID-19] explode all trees  #3 #1 AND #2 |
