# Supplementary material for: A deeper look at long-term effects of COVID-19 on myocardial function in survivors with no prior heart diseases: a GRADE approach systematic review and meta-analysis
Source: Front Cardiovasc Med. 2024 Nov 19;11:1458389. doi: 10.3389/fcvm.2024.1458389 (PMC11611865; doi:10.3389/fcvm.2024.1458389)
Supplement: Supplementary S3 Document — Table of excluded studies during final screening. [file Datasheet3.docx]

**S3. document. Exclusion Table**

| **Author’s Name, Year of publication** | **Name of the Article** | **Reason of Exclusion** |
| --- | --- | --- |
| Ahmed et al. (1) 2023 | COVID-19 and Cardiomyopathy in African Americans: An Early Single-Center Experience | Patients with pre-existing cardiac diseases were not excluded. |
| Back et al. (2) 2022 | Mild-to-moderate COVID-19 impact on the cardiorespiratory fitness in young and middle-aged populations | Cardiovascular function was evaluated by other imaging techniques. |
| Baruch et al. (3) 2011 | Evolution of right and left ventricle routine and speckle-tracking echocardiography in patients recovering from coronavirus disease 2019: a longitudinal study | Patients with pre-existing cardiac diseases were not excluded. |
| Baum et al. (4) 2022 | Cardiac function in relation to functional status and fatigue in patients with post‐COVID syndrome | Patients with pre-existing cardiac diseases were not excluded. |
| Begić et al. (5) 2022 | Pericardial Effusion in Postcoronavirus Disease Patients with Preserved Ejection Fraction of the Left Ventricle and Normal Values of N‐Terminal‐Pro B‐Type Natriuretic Peptide‐Link with C‐Reactive Protein and D‐Dimer | Echocardiography was performed during the acute phase of covid-19. |
| Widmann et al. (6) 2023 | COVID‐19 in Female and Male Athletes: Symptoms, Clinical Findings, Outcome, and Prolonged Exercise Intolerance—A Prospective, Observational, Multicenter Cohort Study (CoSmo‐S) | Echocardiography was performed during the acute phase of covid-19. |
| Cecchetto et al. (7) 2023 | Dyspnea in Post-Acute COVID-19: A Multi-Parametric Cardiopulmonary Evaluation | Patients with pre-existing cardiac diseases were not excluded. |
| Cecchetto et al. (8) 2023 | Subclinical Myocardial Injury in Patients Recovered from COVID-19 Pneumonia: Predictors and Longitudinal Assessment | Patients with pre-existing cardiac diseases were not excluded. |
| Gao et al. (9) 2021 | Normalized Cardiac Structure and Function in COVID-19 Survivors Late After Recovery | Patients with pre-existing cardiac diseases were not excluded. |
| Garcia‐Zamora et al. 2022 | Abnormal echocardiographic findings after COVID‐19 infection: a multicenter registry | Patients with pre-existing cardiac diseases were not excluded. |
| Hanneman et al. (10) 2023 | Multimodality Cardiac Imaging, Cardiac Symptoms, and Clinical Outcomes in Patients Who Recovered from Mild COVID-19 | Patients with pre-existing cardiac diseases were not excluded. |
| Honchar et al. (11) 2023 | A prognostic model and pre-discharge predictors of post-COVID-19 syndrome after hospitalization for SARS-CoV-2 infection | Echocardiography was performed during the acute phase of covid-19. |
| Ingul et al. (12) 2022 | Cardiac Dysfunction and Arrhythmias  3 Months After Hospitalization for COVID-19 | Patients with pre-existing cardiac diseases were not excluded. |
| Joy et al. (13) 2021 | Prospective Case-Control Study of Cardiovascular Abnormalities 6 Months Following Mild COVID-19 in Healthcare Workers | Cardiovascular function was evaluated by other imaging techniques. |
| Komici et al. (14) 2021 | Clinical Characteristics, Exercise Capacity and Pulmonary Function in Post-COVID-19 Competitive Athletes | Patients with pre-existing cardiac diseases were not excluded. |
| Lassen et al. (15) 2021 | Recovery of cardiac function following COVID-19 – ECHOVID-19: a prospective longitudinal cohort study | Patients with pre-existing cardiac diseases were not excluded. |
| Kelly et al. (16) 2021 | Reduced Cardiac Function by Echocardiography in a Minority of COVID-19 Patients 3 Months after Hospitalization | Patients with pre-existing cardiac diseases were not excluded. |
| Mahmoud et al. | Cardiovascular symptom phenotypes of post-acute sequelae of SARS-CoV-2 | Patients with pre-existing cardiac diseases were not excluded. |
| Sahanic et al. (17) 2023 | COVID-19 and its continuing burden after 12 months: a longitudinal observational prospective multicentre trial | Patients with pre-existing cardiac diseases were not excluded. |
| Sabatino et al. (18) 2022 | Mid- and Long-Term Atrio-Ventricular Functional Changes in Children after Recovery from COVID-19 | Patients with pre-existing cardiac diseases were not excluded. |
| Ribeiro Baptista et al. (19) 2022 | Identification of factors impairing exercise capacity after severe COVID-19 pulmonary infection: a 3-month follow-up of prospective COVulnerability cohort | Patients with pre-existing cardiac diseases were not excluded. |
| Nuzzi et al. (20) 2021 | Impaired Right Ventricular Longitudinal Strain Without Pulmonary Hypertension in Patients Who Have Recovered From COVID-19 | Patients with pre-existing cardiac diseases were not excluded. |
| Raafs et al. (21) 2022 | Cardiovascular outcome 6 months after severe coronavirus disease 2019 infection | Patients with pre-existing cardiac diseases were not excluded. |
| Pelà et al. (22) 2021 | Long-Term Cardiac Sequelae in Patients Referred into a Diagnostic Post-COVID-19 Pathway: The Different Impacts on the Right and Left Ventricles | Patients with pre-existing cardiac diseases were not excluded. |
| Øvrebotten et al. (23) 2023 | Minor Myocardial Scars in Association with Cardiopulmonary Function after COVID-19 | Patients with pre-existing cardiac diseases were not excluded. |
| Øvrebotten et al. (24) 2022 | Changes in cardiac structure and function from 3 to 12 months after hospitalization for COVID‐19 | Patients with pre-existing cardiac diseases were not excluded. |
| Sechi et al. (25) 2021 | Short‐term cardiac outcome in survivors of COVID‐19: a systematic study after hospital discharge | Patients with pre-existing cardiac diseases were not excluded. |
| Oikonomou et al. (26) 2023 | Impaired left ventricular deformation and ventricular‐arterial coupling in post‐COVID‐19: association with autonomic dysregulation | Patients with pre-existing cardiac diseases were not excluded. |
| Niebauer et al. (27) 2023 | Cardiopulmonary Long-Term Sequelae in Patients after Severe COVID-19 Disease | Patients with pre-existing cardiac diseases were not excluded. |
| Petersen et al. (28) 2022 | Multi-organ assessment in mainly non- hospitalized individuals after SARS-CoV-2 infection: The Hamburg City Health Study COVID programme | Patients with pre-existing cardiac diseases were not excluded. |
| Cannata et al. (29) 2023 | Long-term prognostic impact of subclinical myocardial dysfunction in patients recovered from COVID-19 | Patients with pre-existing cardiac diseases were not excluded. |
| Ergül et al. (30) 2022 | Effect of Coronavirus Disease-2019 Infection on Left Atrial Functions | Patients with pre-existing cardiac diseases were not excluded. |
| Aparisi et al. (31) 2022 | Cardio-Pulmonary Dysfunction Evaluation in Patients with Persistent Post-COVID-19 Headache | Patients with pre-existing cardiac diseases were not excluded. |
| Jimeno-Almazán et al. (32) 2021 | Chronotropic Incompetence in Non-Hospitalized Patients with Post-COVID-19 Syndrome | Patients with pre-existing cardiac diseases were not excluded. |
| Lassen et al. (15) 2021 | Recovery of cardiac function following COVID-19 – ECHOVID-19: a prospective longitudinal cohort study | Patients with pre-existing cardiac diseases were not excluded. |
| van den Heuvel et al.  (33) 2021 | Comparison between myocardial function assessed by echocardiography during hospitalization for COVID‐19 and at 4 months follow‐up | Patients with pre-existing cardiac diseases were not excluded. |
| Yaroslavskaya et al. (34) 2022 | Comparison of clinical and echocardiographic parameters of patients with COVID-19 pneumonia three months and one year after discharge | Patients with pre-existing cardiac diseases were not excluded. |
| Thornton et al. (35) 2021 | Myocardial Perfusion Imaging After Severe COVID-19 Infection Demonstrates Regional Ischemia Rather Than Global Blood Flow Reduction | Cardiovascular function was evaluated by other imaging techniques. |
| McIntosh et al. (36) 2023 | Echocardiographic findings in non-hospitalised children and adolescents following acute COVID-19 | Echocardiography was performed during the acute phase of covid-19. |
| Özdemir et al. (37) 2022 | Strain Echocardiographic Evaluation of Myocardial Involvement in Patients with Continuing Chest Pain after COVID-19 Infection | Echocardiography was performed during the acute phase of covid-19. |
| Sonnweber et al. (38) 2021 | Cardiopulmonary recovery after COVID-19: an observational prospective multicentre trial | Patients with pre-existing cardiac diseases were not excluded. |
| Rameshwar et al. (39) 2022 | Post-covid assessment of right and left ventricular global longitudinal strain | Study group of patients were not well-defined. |
| Catena et al. (40) 2020 | Echocardiographic Comparison of COVID-19 Patients with or without Prior Biochemical Evidence of Cardiac Injury after Recovery | Study group of patients were not well-defined |
| Augest et al.(41) 2021 | Complaints and clinical findings six months after COVID-19: outpatient follow-up at the University Medical Center Freiburg | Full text not found. |

1. Ahmed A, Assaf AD, Khamooshi N, Brannan GD, Saba S, Zughaib ME. COVID-19 and Cardiomyopathy in African Americans: An Early Single-Center Experience. Cureus. 2023;15(5):e38529.

2. Back GD, Oliveira MR, Camargo PF, Goulart CL, Oliveira CR, Wende KW, et al. Mild-to-moderate COVID-19 impact on the cardiorespiratory fitness in young and middle-aged populations. Braz J Med Biol Res. 2022;55:e12118.

3. Baruch G, Rothschild E, Sadon S, Szekely Y, Lichter Y, Kaplan A, et al. Evolution of right and left ventricle routine and speckle-tracking echocardiography in patients recovering from coronavirus disease 2019: a longitudinal study. Eur Heart J Cardiovasc Imaging. 2022;23(8):1055-65.

4. Baum P, Do L, Deterding L, Lier J, Kunis I, Saur D, et al. Cardiac function in relation to functional status and fatigue in patients with post-COVID syndrome. Sci Rep. 2022;12(1):19575.

5. Begić E, Iglica A, Gojak R, Baljić R, Begić Z, Durak-Nalbantić A, et al. Pericardial Effusion in Postcoronavirus Disease Patients with Preserved Ejection Fraction of the Left Ventricle and Normal Values of N-Terminal-Pro B-Type Natriuretic Peptide-Link with C-Reactive Protein and D-Dimer. Int J Appl Basic Med Res. 2022;12(3):157-60.

6. Widmann M, Gaidai R, Schubert I, Grummt M, Bensen L, Kerling A, et al. COVID-19 in Female and Male Athletes: Symptoms, Clinical Findings, Outcome, and Prolonged Exercise Intolerance-A Prospective, Observational, Multicenter Cohort Study (CoSmo-S). Sports Med. 2024.

7. Cecchetto A, Guarnieri G, Torreggiani G, Vianello A, Baroni G, Palermo C, et al. Dyspnea in Post-Acute COVID-19: A Multi-Parametric Cardiopulmonary Evaluation. J Clin Med. 2023;12(14).

8. Cecchetto A, Torreggiani G, Guarnieri G, Vianello A, Baroni G, Palermo C, et al. Subclinical Myocardial Injury in Patients Recovered from COVID-19 Pneumonia: Predictors and Longitudinal Assessment. J Cardiovasc Dev Dis. 2023;10(4).

9. Gao YP, Zhou W, Huang PN, Liu HY, Bi XJ, Zhu Y, et al. Normalized Cardiac Structure and Function in COVID-19 Survivors Late After Recovery. Front Cardiovasc Med. 2021;8:756790.

10. Hanneman K, Houbois C, Kei T, Gustafson D, Thampinathan B, Sooriyakanthan M, et al. Multimodality Cardiac Imaging, Cardiac Symptoms, and Clinical Outcomes in Patients Who Recovered from Mild COVID-19. Radiology. 2023;308(1):e230767.

11. Honchar O, Ashcheulova T, Chumachenko T, Chumachenko D, Bobeiko A, Blazhko V, et al. A prognostic model and pre-discharge predictors of post-COVID-19 syndrome after hospitalization for SARS-CoV-2 infection. Front Public Health. 2023;11:1276211.

12. Ingul CB, Grimsmo J, Mecinaj A, Trebinjac D, Berger Nossen M, Andrup S, et al. Cardiac Dysfunction and Arrhythmias 3 Months After Hospitalization for COVID-19. J Am Heart Assoc. 2022;11(3):e023473.

13. Joy G, Artico J, Kurdi H, Seraphim A, Lau C, Thornton GD, et al. Prospective Case-Control Study of Cardiovascular Abnormalities 6 Months Following Mild COVID-19 in Healthcare Workers. JACC Cardiovasc Imaging. 2021;14(11):2155-66.

14. Komici K, Bianco A, Perrotta F, Dello Iacono A, Bencivenga L, D'Agnano V, et al. Clinical Characteristics, Exercise Capacity and Pulmonary Function in Post-COVID-19 Competitive Athletes. J Clin Med. 2021;10(14).

15. Lassen MCH, Skaarup KG, Lind JN, Alhakak AS, Sengeløv M, Nielsen AB, et al. Recovery of cardiac function following COVID-19 - ECHOVID-19: a prospective longitudinal cohort study. Eur J Heart Fail. 2021;23(11):1903-12.

16. Tangen J, Aukrust P, Barratt-Due A, Skulstad H, Edvardsen T. Reduced Cardiac Function by Echocardiography in a Minority of COVID-19 Patients 3 Months after Hospitalization. J Am Soc Echocardiogr. 2022;35(2):243-4.

17. Sahanic S, Tymoszuk P, Luger AK, Hüfner K, Boehm A, Pizzini A, et al. COVID-19 and its continuing burden after 12 months: a longitudinal observational prospective multicentre trial. ERJ Open Res. 2023;9(2).

18. Sabatino J, Di Chiara C, Di Candia A, Sirico D, Donà D, Fumanelli J, et al. Mid- and Long-Term Atrio-Ventricular Functional Changes in Children after Recovery from COVID-19. J Clin Med. 2022;12(1).

19. Ribeiro Baptista B, d'Humières T, Schlemmer F, Bendib I, Justeau G, Al-Assaad L, et al. Identification of factors impairing exercise capacity after severe COVID-19 pulmonary infection: a 3-month follow-up of prospective COVulnerability cohort. Respir Res. 2022;23(1):68.

20. Nuzzi V, Castrichini M, Collini V, Roman-Pognuz E, Di Bella S, Luzzati R, et al. Impaired Right Ventricular Longitudinal Strain Without Pulmonary Hypertension in Patients Who Have Recovered From COVID-19. Circ Cardiovasc Imaging. 2021;14(4):e012166.

21. Raafs AG, Ghossein MA, Brandt Y, Henkens M, Kooi ME, Vernooy K, et al. Cardiovascular outcome 6 months after severe coronavirus disease 2019 infection. J Hypertens. 2022;40(7):1278-87.

22. Pelà G, Goldoni M, Cavalli C, Perrino F, Tagliaferri S, Frizzelli A, et al. Long-Term Cardiac Sequelae in Patients Referred into a Diagnostic Post-COVID-19 Pathway: The Different Impacts on the Right and Left Ventricles. Diagnostics (Basel). 2021;11(11).

23. Øvrebotten T, Heck S, Skjørten I, Einvik G, Stavem K, Ingul CB, et al. Minor Myocardial Scars in Association with Cardiopulmonary Function after COVID-19. Cardiology. 2023;148(4):300-6.

24. Øvrebotten T, Myhre P, Grimsmo J, Mecinaj A, Trebinjac D, Nossen MB, et al. Changes in cardiac structure and function from 3 to 12 months after hospitalization for COVID-19. Clin Cardiol. 2022;45(10):1044-52.

25. Sechi LA, Colussi G, Bulfone L, Brosolo G, Da Porto A, Peghin M, et al. Short-term cardiac outcome in survivors of COVID-19: a systematic study after hospital discharge. Clin Res Cardiol. 2021;110(7):1063-72.

26. Oikonomou E, Lampsas S, Theofilis P, Souvaliotis N, Papamikroulis GA, Katsarou O, et al. Impaired left ventricular deformation and ventricular-arterial coupling in post-COVID-19: association with autonomic dysregulation. Heart Vessels. 2023;38(3):381-93.

27. Niebauer JH, Binder-Rodriguez C, Iscel A, Schedl S, Capelle C, Kahr M, et al. Cardiopulmonary Long-Term Sequelae in Patients after Severe COVID-19 Disease. J Clin Med. 2023;12(4).

28. Petersen EL, Goßling A, Adam G, Aepfelbacher M, Behrendt CA, Cavus E, et al. Multi-organ assessment in mainly non-hospitalized individuals after SARS-CoV-2 infection: The Hamburg City Health Study COVID programme. Eur Heart J. 2022;43(11):1124-37.

29. Cannata F, Pinto G, Chiarito M, Maurina M, Condello F, Bombace S, et al. Long-term prognostic impact of subclinical myocardial dysfunction in patients recovered from COVID-19. Echocardiography. 2023;40(6):464-74.

30. Ergül E, Özyildiz AG, Emlek N, Özyildiz A, Duman H, Çetin M. Effect of Coronavirus Disease-2019 Infection on Left Atrial Functions. J Cardiovasc Echogr. 2022;32(2):89-94.

31. Aparisi Á, Ybarra-Falcón C, Iglesias-Echeverría C, García-Gómez M, Marcos-Mangas M, Valle-Peñacoba G, et al. Cardio-Pulmonary Dysfunction Evaluation in Patients with Persistent Post-COVID-19 Headache. Int J Environ Res Public Health. 2022;19(7).

32. Jimeno-Almazán A, Pallarés JG, Buendía-Romero Á, Martínez-Cava A, Courel-Ibáñez J. Chronotropic Incompetence in Non-Hospitalized Patients with Post-COVID-19 Syndrome. J Clin Med. 2021;10(22).

33. van den Heuvel FMA, Vos JL, van Bakel B, Duijnhouwer AL, van Dijk APJ, Dimitriu-Leen AC, et al. Comparison between myocardial function assessed by echocardiography during hospitalization for COVID-19 and at 4 months follow-up. Int J Cardiovasc Imaging. 2021;37(12):3459-67.

34. Yaroslavskaya EI, Krinochkin DV, Shirokov NE, Gorbatenko EA, Krinochkina IR, Gultyaeva EP, et al. Comparison of clinical and echocardiographic parameters of patients with COVID-19 pneumonia three months and one year after discharge. Kardiologiia. 2022;62(1):13-23.

35. Thornton GD, Shetye A, Knight DS, Knott K, Artico J, Kurdi H, et al. Myocardial Perfusion Imaging After Severe COVID-19 Infection Demonstrates Regional Ischemia Rather Than Global Blood Flow Reduction. Front Cardiovasc Med. 2021;8:764599.

36. McIntosh AM, Goyal A, Moser-Dungan C, Harvey B, Heching HJ, Aly DG, et al. Echocardiographic findings in non-hospitalised children and adolescents following acute COVID-19. Cardiol Young. 2023:1-7.

37. Özdemir E, Karagöz U, Emren SV, Altay S, Eren NK, Özdemir S, Tokaç M. Strain Echocardiographic Evaluation of Myocardial Involvement in Patients with Continuing Chest Pain after COVID-19 Infection. Arq Bras Cardiol. 2023;120(1):e20220287.

38. Sonnweber T, Sahanic S, Pizzini A, Luger A, Schwabl C, Sonnweber B, et al. Cardiopulmonary recovery after COVID-19: an observational prospective multicentre trial. Eur Respir J. 2021;57(4).

39. Rameshwar R, Meenakshi K, Hanumanram G, Kannan R, Magesh Kumar S, Damodaran J, Nandhini S. Post covid assesment of right and left ventricular global longitudinal strain. Indian Heart J. 2022;74(2):144-7.

40. Catena C, Colussi G, Bulfone L, Da Porto A, Tascini C, Sechi LA. Echocardiographic Comparison of COVID-19 Patients with or without Prior Biochemical Evidence of Cardiac Injury after Recovery. J Am Soc Echocardiogr. 2021;34(2):193-5.

41. August D, Stete K, Hilger H, Götz V, Biever P, Hosp J, et al. [Complaints and clinical findings six months after COVID-19: outpatient follow-up at the University Medical Center Freiburg]. Dtsch Med Wochenschr. 2021;146(17):e65-e73.
