# Supplementary material for: A deeper look at long-term effects of COVID-19 on myocardial function in survivors with no prior heart diseases: a GRADE approach systematic review and meta-analysis
Source: Front Cardiovasc Med. 2024 Nov 19;11:1458389. doi: 10.3389/fcvm.2024.1458389 (PMC11611865; doi:10.3389/fcvm.2024.1458389)
Supplement: Supplementary S4 Document — Forest plots of meta-analysis. [file Datasheet4.docx]

| **LV geometric indices** |
| --- |

| **LVEDD** |
| --- |
| A   |
| B   |
| C   |
| D   |
| Forest plot graphs for the comparison of LVEDD between patients recovered from COVID-19 infection and the control group. **Panel A** indicates over all analysis of LVEDD reported in studies. **Panel B** shows subgroup analysis grouped by duration between post-COVID-19 patients and control group. **Panel C** shows subgroup analysis grouped by the severity of the infection. **Panel D** shows subgroup analysis grouped by the presence of comorbid diseases. Both random-effect and fixed-effect models were used in this meta-analysis, and MD were used to measure the effect size. A positive effect indicates higher values in post COVID-19 patients compared to controls and vice versa. CI= confidence interval, SD= standard deviation, MD= mean differences, LVEDD= left ventricular end-diastolic diameter |

| **LVEDV** |
| --- |
| A   |
| B   |
| C   |
| D   |
| Forest plot graphs for the comparison of LVEDV between patients recovered from COVID-19 infection and the control group. **Panel A** indicates over all analysis of LVEDV reported in studies. **Panel B** shows subgroup analysis grouped by duration between post-COVID-19 patients and control group. **Panel C** shows subgroup analysis grouped by the severity of the infection. **Panel D** shows subgroup analysis grouped by the presence of comorbid diseases. Both random-effect and fixed-effect models were used in this meta-analysis, and MD were used to measure the effect size. A positive effect indicates higher values in post COVID-19 patients compared to controls and vice versa. CI= confidence interval, SD= standard deviation, MD= mean differences, LVEDV= left ventricular end-diastolic volume |

| **LVESD** |
| --- |
| A   |
| B   |
| C   |
| D   |
| Forest plot graphs for the comparison of LVESD between patients recovered from COVID-19 infection and the control group. **Panel A** indicates over all analysis of LVESD reported in studies. **Panel B** shows subgroup analysis grouped by duration between post-COVID-19 patients and control group. **Panel C** shows subgroup analysis grouped by the severity of the infection. **Panel D** shows subgroup analysis grouped by the presence of comorbid diseases. Both random-effect and fixed-effect models were used in this meta-analysis, and MD were used to measure the effect size. A positive effect indicates higher values in post COVID-19 patients compared to controls and vice versa. CI= confidence interval, SD= standard deviation, MD= mean differences, LVESD= left ventricular end-systolic diameter |

| **LVESV** |
| --- |
| A   |
| B   |
| C   |
| D   |
| Forest plot graphs for the comparison of LVESV between patients recovered from COVID-19 infection and the control group. **Panel A** indicates over all analysis of LVESV reported in studies. **Panel B** shows subgroup analysis grouped by duration between post-COVID-19 patients and control group. **Panel C** shows subgroup analysis grouped by the severity of the infection. **Panel D** shows subgroup analysis grouped by the presence of comorbid diseases. Both random-effect and fixed-effect models were used in this meta-analysis, and MD were used to measure the effect size. A positive effect indicates higher values in post COVID-19 patients compared to controls and vice versa. CI= confidence interval, SD= standard deviation, MD= mean differences, LVESV= left ventricular end-systolic volume |

| **IVSD** |
| --- |
| A   |
| B   |
| C   |
| D   |
| Forest plot graphs for the comparison of IVSD between patients recovered from COVID-19 infection and the control group. **Panel A** indicates over all analysis of IVSD reported in studies. **Panel B** shows subgroup analysis grouped by duration between post-COVID-19 patients and control group. **Panel C** shows subgroup analysis grouped by the severity of the infection. **Panel D** shows subgroup analysis grouped by the presence of comorbid diseases. Both random-effect and fixed-effect models were used in this meta-analysis, and MD were used to measure the effect size. A positive effect indicates higher values in post COVID-19 patients compared to controls and vice versa. CI= confidence interval, SD= standard deviation, MD= mean differences, IVSD= interventricular septum diameter |

| **PWD** |
| --- |
| A   |
| B   |
| C   |
| D   |
| Forest plot graphs for the comparison of PWS between patients recovered from COVID-19 infection and the control group. **Panel A** indicates over all analysis of PWD reported in studies. **Panel B** shows subgroup analysis grouped by duration between post-COVID-19 patients and control group. **Panel C** shows subgroup analysis grouped by the severity of the infection. **Panel D** shows subgroup analysis grouped by the presence of comorbid diseases. Both random-effect and fixed-effect models were used in this meta-analysis, and MD were used to measure the effect size. A positive effect indicates higher values in post COVID-19 patients compared to controls and vice versa. CI= confidence interval, SD= standard deviation, MD= mean differences, PWD= posterior wall diameter |

| **LVM** |
| --- |
| A   |
| B   |
| C   |
| D   |
| Forest plot graphs for the comparison of LVM between patients recovered from COVID-19 infection and the control group. **Panel A** indicates over all analysis of LVM reported in studies. **Panel B** shows subgroup analysis grouped by duration between post-COVID-19 patients and control group. **Panel C** shows subgroup analysis grouped by the severity of the infection. **Panel D** shows subgroup analysis grouped by the presence of comorbid diseases. Both random-effect and fixed-effect models were used in this meta-analysis, and MD were used to measure the effect size. A positive effect indicates higher values in post COVID-19 patients compared to controls and vice versa. CI= confidence interval, SD= standard deviation, MD= mean differences, LVM= left ventricular mass |

| **LVMI** |
| --- |
| A   |
| B   |
| C   |
| D   |
| Forest plot graphs for the comparison of LVMI between patients recovered from COVID-19 infection and the control group. **Panel A** indicates over all analysis of LVMI reported in studies. **Panel B** shows subgroup analysis grouped by duration between post-COVID-19 patients and control group. **Panel C** shows subgroup analysis grouped by the severity of the infection. **Panel D** shows subgroup analysis grouped by the presence of comorbid diseases. Both random-effect and fixed-effect models were used in this meta-analysis, and MD were used to measure the effect size. A positive effect indicates higher values in post COVID-19 patients compared to controls and vice versa. CI= confidence interval, SD= standard deviation, MD= mean differences, LVMI= left ventricular mass index |

| **LV Systolic function** |
| --- |

| **LVEF** |
| --- |
| A   |
| B   |
| C   |
| D   |
| Forest plot graphs for the comparison of LVEF between patients recovered from COVID-19 infection and the control group. **Panel A** indicates over all analysis of LVEF reported in studies. **Panel B** shows subgroup analysis grouped by duration between post-COVID-19 patients and control group. **Panel C** shows subgroup analysis grouped by the severity of the infection. **Panel D** shows subgroup analysis grouped by the presence of comorbid diseases. Both random-effect and fixed-effect models were used in this meta-analysis, and MD were used to measure the effect size. A positive effect indicates higher values in post COVID-19 patients compared to controls and vice versa. CI= confidence interval, SD= standard deviation, MD= mean differences, LVEF= left ventricular ejection fraction |

| **LV-GLS** |
| --- |
| A   |
| B   |
| C   |
| D   |
| Forest plot graphs for the comparison of LV-GLS between patients recovered from COVID-19 infection and the control group. **Panel A** indicates over all analysis of LV-GLS reported in studies. **Panel B** shows subgroup analysis grouped by duration between post-COVID-19 patients and control group. **Panel C** shows subgroup analysis grouped by the severity of the infection. **Panel D** shows subgroup analysis grouped by the presence of comorbid diseases. A random-effects model was used in this meta-analysis, and MD were used to measure the effect size. A positive effect indicates higher values in post COVID-19 patients compared to controls and vice versa. CI= confidence interval, SD= standard deviation, MD= mean differences, LV-GLS= left ventricular global longitudinal strain |

| **LV diastolic function** |
| --- |

| **E/A Ratio** |
| --- |
| A   |
| B   |
| C   |
| D   |
| Forest plot graphs for the comparison of E/A Ratio between patients recovered from COVID-19 infection and the control group. **Panel A** indicates over all analysis of E/A Ratio reported in studies. **Panel B** shows subgroup analysis grouped by duration between post-COVID-19 patients and control group. **Panel C** shows subgroup analysis grouped by the severity of the infection. **Panel D** shows subgroup analysis grouped by the presence of comorbid diseases. Both random-effect and fixed-effect models were used in this meta-analysis, and MD were used to measure the effect size. A positive effect indicates higher values in post COVID-19 patients compared to controls and vice versa. CI= confidence interval, SD= standard deviation, MD= mean differences, E/A Ratio = the ratio of peak velocity blood flow from left ventricular relaxation in early diastole (the E wave) to peak velocity flow in late diastole caused by atrial contraction (the A wave) |

| **E/e’ ration** |
| --- |
| A   |
| B   |
| C   |
| D   |
| Forest plot graphs for the comparison of E/e’ ration between patients recovered from COVID-19 infection and the control group. **Panel A** indicates over all analysis of E/e’ ration reported in studies. **Panel B** shows subgroup analysis grouped by duration between post-COVID-19 patients and control group. **Panel C** shows subgroup analysis grouped by the severity of the infection. **Panel D** shows subgroup analysis grouped by the presence of comorbid diseases. Both random-effect and fixed-effect models were used in this meta-analysis, and MD were used to measure the effect size. A positive effect indicates higher values in post COVID-19 patients compared to controls and vice versa. CI= confidence interval, SD= standard deviation, MD= mean differences, E/e’ ration = ratio of E wave to early diastolic mitral annular velocity (E’) |

| **Mitral E wave** |
| --- |
| A   |
| B   |
| C   |
| D   |
| Forest plot graphs for the comparison of Mitral E wave between patients recovered from COVID-19 infection and the control group. **Panel A** indicates over all analysis of Mitral E wave reported in studies. **Panel B** shows subgroup analysis grouped by duration between post-COVID-19 patients and control group. **Panel C** shows subgroup analysis grouped by the severity of the infection. **Panel D** shows subgroup analysis grouped by the presence of comorbid diseases. Both random-effect and fixed-effect models were used in this meta-analysis, and MD were used to measure the effect size. A positive effect indicates higher values in post COVID-19 patients compared to controls and vice versa. CI= confidence interval, SD= standard deviation, MD= mean differences |

| **Mitral A wave** |
| --- |
| A   |
| B   |
| C   |
| D   |
| Forest plot graphs for the comparison of Mitral A wave between patients recovered from COVID-19 infection and the control group. **Panel A** indicates over all analysis of Mitral A wave reported in studies. **Panel B** shows subgroup analysis grouped by duration between post-COVID-19 patients and control group. **Panel C** shows subgroup analysis grouped by the severity of the infection. **Panel D** shows subgroup analysis grouped by the presence of comorbid diseases. Both random-effect and fixed-effect models were used in this meta-analysis, and MD were used to measure the effect size. A positive effect indicates higher values in post COVID-19 patients compared to controls and vice versa. CI= confidence interval, SD= standard deviation, MD= mean differences |

| **Left Atrium** |
| --- |

| **LAD** |
| --- |
| A   |
| B   |
| C   |
| D   |
| Forest plot graphs for the comparison of LAD between patients recovered from COVID-19 infection and the control group. **Panel A** indicates over all analysis of LAD reported in studies. **Panel B** shows subgroup analysis grouped by duration between post-COVID-19 patients and control group. **Panel C** shows subgroup analysis grouped by the severity of the infection. **Panel D** shows subgroup analysis grouped by the presence of comorbid diseases. A random-effects model was used in this meta-analysis, and MD were used to measure the effect size. A positive effect indicates higher values in post COVID-19 patients compared to controls and vice versa. CI= confidence interval, SD= standard deviation, MD= mean differences, LAD= left atrium diameter |

| **LAVI** |
| --- |
| A   |
| B   |
| C   |
| D   |
| Forest plot graphs for the comparison of LAVI between patients recovered from COVID-19 infection and the control group. **Panel A** indicates over all analysis of LAVI reported in studies. **Panel B** shows subgroup analysis grouped by duration between post-COVID-19 patients and control group. **Panel C** shows subgroup analysis grouped by the severity of the infection. **Panel D** shows subgroup analysis grouped by the presence of comorbid diseases. Both random-effect and fixed-effect models were used in this meta-analysis, and MD were used to measure the effect size. A positive effect indicates higher values in post COVID-19 patients compared to controls and vice versa. CI= confidence interval, SD= standard deviation, MD= mean differences, LAVI= left atrium volume index |

| **Right Heart function** |
| --- |

| **RV-GLS** |
| --- |
| A   |
| B   |
| C   |
| D   |
| Forest plot graphs for the comparison of RV-GLS between patients recovered from COVID-19 infection and the control group. **Panel A** indicates over all analysis of RV-GLS reported in studies. **Panel B** shows subgroup analysis grouped by duration between Post-COVID-19 patients and control group. **Panel C** shows subgroup analysis grouped by the severity of the infection. **Panel D** shows subgroup analysis grouped by the presence of comorbid diseases. A random-effects model was used in this meta-analysis, and MD were used to measure the effect size. A positive effect indicates higher values in post COVID-19 patients compared to controls and vice versa. CI= confidence interval, SD= standard deviation, MD= mean differences, RV-GLS = right ventricular global longitudinal strain |

| **RV-MPI** |
| --- |
| A   |
| B   |
| C   |
| D   |
| Forest plot graphs for the comparison of RV-MPI between patients recovered from COVID-19 infection and the control group. **Panel A** indicates over all analysis of RV-MPI reported in studies. **Panel B** shows subgroup analysis grouped by duration between post-COVID-19 patients and control group. **Panel C** shows subgroup analysis grouped by the severity of the infection. **Panel D** shows subgroup analysis grouped by the presence of comorbid diseases. A random-effects model was used in this meta-analysis, and MD were used to measure the effect size. A positive effect indicates higher values in post COVID-19 patients compared to controls and vice versa. CI= confidence interval, SD= standard deviation, MD= mean differences, RV-MPI= right ventricular myocardial performance index |

| **RV diameter** |
| --- |
| A   |
| B   |
| C   |
| D   |
| Forest plot graphs for the comparison of RV diameter between patients recovered from COVID-19 infection and the control group. **Panel A** indicates over all analysis of RV diameter reported in studies. **Panel B** shows subgroup analysis grouped by duration between Post-COVID-19 patients and control group. **Panel C** shows subgroup analysis grouped by the severity of the infection. **Panel D** shows subgroup analysis grouped by the presence of comorbid diseases. Both random-effect and fixed-effect models were used in this meta-analysis, and MD were used to measure the effect size. A positive effect indicates higher values in post COVID-19 patients compared to controls and vice versa. CI= confidence interval, SD= standard deviation, MD= mean differences, RV diameter= right ventricular diameter |

| **RAD** |
| --- |
| A   |
| B   |
| C   |
| D   |
| Forest plot graphs for the comparison of RAD between patients recovered from COVID-19 infection and the control group. **Panel A** indicates over all analysis of RAD reported in studies. **Panel B** shows subgroup analysis grouped by duration between post-COVID-19 patients and control group. **Panel C** shows subgroup analysis grouped by the severity of the infection. **Panel D** shows subgroup analysis grouped by the presence of comorbid diseases. Both random-effect and fixed-effect models were used in this meta-analysis, and MD were used to measure the effect size. A positive effect indicates higher values in post COVID-19 patients compared to controls and vice versa. CI= confidence interval, SD= standard deviation, MD= mean differences, RAD= right atrium diameter |

| **TAPSE** |
| --- |
| A   |
| B   |
| C   |
| D   |
| Forest plot graphs for the comparison of TAPSE between patients recovered from COVID-19 infection and the control group. **Panel A** indicates over all analysis of TAPSE reported in studies. **Panel B** shows subgroup analysis grouped by duration between Post-COVID-19 patients and control group. **Panel C** shows subgroup analysis grouped by the severity of the infection. **Panel D** shows subgroup analysis grouped by the presence of comorbid diseases. A random-effects model was used in this meta-analysis, and MD were used to measure the effect size. A positive effect indicates higher values in post COVID-19 patients compared to controls and vice versa. CI= confidence interval, SD= standard deviation, MD= mean differences, TAPSE= tricuspid annular plane systolic excursion |

| **sPAP** |
| --- |
| A   |
| B   |
| C   |
| D   |
| Forest plot graphs for the comparison of sPAP between patients recovered from COVID-19 infection and the control group. **Panel A** indicates over all analysis of sPAP reported in studies. **Panel B** shows subgroup analysis grouped by duration between Post-COVID-19 patients and control group. **Panel C** shows subgroup analysis grouped by the severity of the infection. **Panel D** shows subgroup analysis grouped by the presence of comorbid diseases. A random-effects model was used in this meta-analysis, and MD were used to measure the effect size. A positive effect indicates higher values in post COVID-19 patients compared to controls and vice versa. CI= confidence interval, SD= standard deviation, MD= mean differences, sPAP = systolic pulmonary artery |
