# Supplementary material for: A deeper look at long-term effects of COVID-19 on myocardial function in survivors with no prior heart diseases: a GRADE approach systematic review and meta-analysis
Source: Front Cardiovasc Med. 2024 Nov 19;11:1458389. doi: 10.3389/fcvm.2024.1458389 (PMC11611865; doi:10.3389/fcvm.2024.1458389)
Supplement: Supplementary S5 Document — Forest plots of sensitivity analysis. [file Datasheet5.docx]

**LVEDV**

**Overall meta-analysis**

This figure represents forest plot of sensitivity analysis for LVEDV meta-analysis of its overall outcome. The study by Wood et al. (2022) showed a high risk of bias, and excluding this study revealed a significant result between post-COVID patients and control group. A positive result is in favor of higher amounts in post-COVID patients compared to controls. A random effect model was used. LVEDV: left ventricular end-diastolic volume; MD: mean difference.

**Grouped by Duration from Acute COVID to Echo Examination in Recovery Phase**

This figure represents forest plot of sensitivity analysis for LVEDV meta-analysis grouped by duration. The study by Wood et al. (2022) showed a high risk of bias, and excluding this study revealed no significant difference between post-COVID patients and control group. A random effect model was used. LVEDV: left ventricular end-diastolic volume; MD: mean difference.

**Grouped by statuts of Comorbid dieases**

This figure represents forest plot of sensitivity analysis for LVEDV meta-analysis grouped by status of comorbid disease. The study by Wood et al. (2022) showed a high risk of bias, and excluding this study revealed no significant difference between post-COVID patients and control group. A random effect model was used. LVEDV: left ventricular end-diastolic volume; MD: mean difference.

**IVSD**

**Overall meta-analysis**

This figure represents forest plot of sensitivity analysis for IVSD meta-analysis of its overall outcome. The studies by Ardahanli et al. (2022) and Akbulut et al. (2022) showed a high risk of bias, and excluding these studies did not change the direction, or statistical significance of the summary estimate. A random effect model was used. IVSD: interventricular septal diameter; MD: mean difference.

**Grouped by Duration from Acute COVID to Echo Examination in Recovery Phase**

This figure represents forest plot of sensitivity analysis for IVSD meta-analysis of its overall outcome. The studies by Ardahanli et al. (2022) and Akbulut et al. (2022) showed a high risk of bias, and excluding these studies did not change the direction, or statistical significance of the summary estimate. A random effect model was used. IVSD: interventricular septal diameter; MD: mean difference.

**Grouped by Severity of COVID-19 infection**

This figure represents forest plot of sensitivity analysis for IVSD meta-analysis grouped by severity of COVID-19 infection. The studies by Ardahanli et al. (2022) and Akbulut et al. (2022) showed a high risk of bias, and excluding these studies changed the direction, and statistical significance of the summary estimate. A positive result is in favor of higher amounts in post-COVID patients compared to controls. A random effect model was used. IVSD: interventricular septal diameter; MD: mean difference.

**Grouped by status of Comorbid diseases**

This figure represents forest plot of sensitivity analysis for IVSD meta-analysis grouped by status of comorbid disease. The studies by Ardahanli et al. (2022) and Akbulut et al. (2022) showed a high risk of bias, and excluding these studies changed the direction, and statistical significance of the summary estimate. A positive result is in favor of higher amounts in post-COVID patients compared to controls. A random effect model was used. IVSD: interventricular septal diameter; MD: mean difference.

**LVM**

**Overall Meta-analysis**

This figure represents forest plot of sensitivity analysis for LVM meta-analysis of its overall outcome. Turpin et al. 2023 and Schellenberg et al. 2023 were at high risk of bias for LVM due to involving athletes as their cases and excluding these studies did not change the direction, or statistical significance of the summary estimate. A random effect model was used. LVM: left ventricular mass; MD: mean difference.

**LVMI**

**Overall Meta-analysis**

This figure represents forest plot of sensitivity analysis for LVMI meta-analysis of its overall outcome. Turpin et al. 2023 was at high risk of bias for LVMI due to involving athletes as their cases and excluding this study did not change the direction, or statistical significance of the summary estimate. A random effect model was used. LVMI: left ventricular mass index; MD: mean difference.

**Grouped by Severity of COVID-19 infection**

This figure represents forest plot of sensitivity analysis for LVMI meta-analysis grouped by severity of infection. Turpin et al. 2023 was at high risk of bias for LVMI due to involving athletes as their cases and excluding this study did not change the direction, or statistical significance of the summary estimate. A random effect model was used. LVMI: left ventricular mass index; MD: mean difference.

**Grouped by status of Comorbid diseases**

This figure represents forest plot of sensitivity analysis for LVMI meta-analysis grouped by comorbid disease. Turpin et al. 2023 was at high risk of bias for LVMI due to involving athletes as their cases and excluding this study did not change the direction, or statistical significance of the summary estimate. A random effect model was used. LVMI: left ventricular mass index; MD: mean difference.

**LVEF**

**Overall Meta-analysis**

This figure represents forest plot of sensitivity analysis for LVEF meta-analysis for its overall outcome. Three studies, conducted by Turpin et al. (2023), Tudoran et al. (2023) and Akbulut et al. (2022), were deemed to have a high risk of bias in relation to LVEF and excluding them changed the significancy of the result to insignificant estimate. A random model was used. LVEF: left ventricular ejection fraction.

**Grouped by Duration from Acute COVID to Echo Examination in Recovery Phase**

This figure represents forest plot of sensitivity analysis for LVEF meta-analysis grouped by duration. Three studies, conducted by Turpin et al. (2023), Tudoran et al. (2023) and Akbulut et al. (2022), were deemed to have a high risk of bias in relation to LVEF and excluding them did not change the direction and significancy of results. A random model was used. LVEF: left ventricular ejection fraction.

**Grouped by Severity of COVID-19 infection**

This figure represents forest plot of sensitivity analysis for LVEF meta-analysis grouped by infection severity. Three studies, conducted by Turpin et al. (2023), Tudoran et al. (2023) and Akbulut et al. (2022), were deemed to have a high risk of bias in relation to LVEF and excluding them changed the significancy of the result to insignificant estimate. A random model was used. LVEF: left ventricular ejection fraction.

**Grouped by status of Comorbid diseases**

This figure represents forest plot of sensitivity analysis for LVEF meta-analysis grouped by comorbid diease. Three studies, conducted by Turpin et al. (2023), Tudoran et al. (2023) and Akbulut et al. (2022), were deemed to have a high risk of bias in relation to LVEF and excluding them changed the significancy of the result to insignificant estimate. A random model was used. LVEF: left ventricular ejection fraction.

**LV-GLS**

**Overall**

This figure represents forest plot of sensitivity analysis for LV-GLS meta-analysis of its overall outcome. Akbulut et al. (2022) was at high risk of bias for this outcome and excluding this study did not change the direction, or statistical significance of the summary estimate. A positive result is in favor of higher amounts in post-COVID patients compared to controls. A random effect model was used. LV-GLS: left ventricular global longitudinal strain; MD: mean difference.

**Grouped by** **Duration from Acute COVID to Echo Examination in Recovery Phase**

This figure represents forest plot of sensitivity analysis for LV-GLS meta-analysis grouped by duration. Akbulut et al. (2022) was at high risk of bias for this outcome and excluding this study did not change the direction, or statistical significance of the summary estimate. A positive result is in favor of higher amounts in post-COVID patients compared to controls. A random effect model was used. LV-GLS: left ventricular global longitudinal strain; MD: mean difference.

**Grouped by Severity of COVID-19 infection**

This figure represents forest plot of sensitivity analysis for LV-GLS meta-analysis grouped by severity of infection. Akbulut et al. (2022) was at high risk of bias for this outcome and excluding this study did not change the direction, or statistical significance of the summary estimate. A positive result is in favor of higher amounts in post-COVID patients compared to controls. A random effect model was used. LV-GLS: left ventricular global longitudinal strain; MD: mean difference.

**E/A ratio**

**Overall Outcome**

This figure represents forest plot of sensitivity analysis for E/A ratio meta-analysis of its overall outcome. Hamdy et al. (2023) was at high risk of bias for this outcome and excluding this study changed the statistical significance of the summary estimate. A negative result is in favor of higher amounts in controls compared to post-COVID patients. A random effect model was used. E/A: ratio of mitral E wave to mitral A wave; MD: mean difference.

**Grouped by** **Duration from Acute COVID to Echo Examination in Recovery Phase**

This figure represents forest plot of sensitivity analysis for E/A ratio meta-analysis of grouped by duration. Hamdy et al. (2023) was at high risk of bias for this outcome and excluding this study changed the statistical significance of the summary estimate. A negative result is in favor of higher amounts in controls compared to post-COVID patients. A random effect model was used. E/A: ratio of mitral E wave to mitral A wave; MD: mean difference.

**Grouped by status of Comorbid diseases**

This figure represents forest plot of sensitivity analysis for E/A ratio meta-analysis of grouped by comorbid disease. Hamdy et al. (2023) was at high risk of bias for this outcome and excluding this study did not change the statistical insignificance of the summary estimate. A negative result is in favor of higher amounts in controls compared to post-COVID patients. A random effect model was used. E/A: ratio of mitral E wave to mitral A wave; MD: mean difference.

**E/e’ ratio**

**Overall**

This figure represents forest plot of sensitivity analysis for E/e’ ratio meta-analysis of its overall outcome. Hamdy et al. (2023) and Wood et al. (2022) were at high risk of bias for this outcome and excluding this study did not change the direction, or statistical significance of the summary estimate. A random effect model was used. E/e’ ratio: ratio of early diastolic mitral annular velocity (E’) to E wave; MD: mean difference.

**Grouped by** **Duration from Acute COVID to Echo Examination in Recovery Phase**

This figure represents forest plot of sensitivity analysis for E/e’ ratio meta-analysis grouped by duration. Hamdy et al. (2023) and Wood et al. (2022) were at high risk of bias for this outcome and excluding this study did not change the direction, or statistical significance of the summary estimate. A random effect model was used. E/e’ ratio: ratio of early diastolic mitral annular velocity (E’) to E wave; MD: mean difference.

**LAVI**

**Overall**

This figure represents forest plot of sensitivity analysis for LAVI meta-analysis of its overall outcome. Hamdy et al. (2023) was at high risk of bias for this outcome and excluding this study did not change the direction, or statistical significance of the summary estimate. A random effect model was used.

**Grouped by Duration from Acute COVID to Echo Examination in Recovery Phase**

This figure represents forest plot of sensitivity analysis for LAVI meta-analysis grouped by duration. Hamdy et al. (2023) was at high risk of bias for this outcome and excluding this study did not change the direction, or statistical significance of the summary estimate. A fixed effect model was used.

**RV-MPI**

This figure represents forest plot of sensitivity analysis for RV-MPI meta-analysis of its overall outcome. Gunay et al. (2021) was at high risk of bias for this outcome and excluding this study did not change statistical significance of the summary estimate. A random effect model was used. RV-MPI: right ventricular myocardial performance index; MD: mean difference.

RVD

Overall

This figure represents forest plot of sensitivity analysis for RVD meta-analysis of its overall outcome. Gunay et al. (2021) was at high risk of bias for this outcome and excluding this study did not change statistical significance of the summary estimate. A positive result is in favor of higher amounts in post-COVID patients compared to controls. A fixed effect model was used. RVD: right ventricular diameter; MD: mean difference.

**Grouped by Duration from Acute COVID to Echo Examination in Recovery Phase**

This figure represents forest plot of sensitivity analysis for RVD meta-analysis grouped by duration. Gunay et al. (2021) was at high risk of bias for this outcome and excluding this study changed statistical significance of the summary estimate. A positive result is in favor of higher amounts in post-COVID patients compared to controls. A fixed effect model was used. RVD: right ventricular diameter; MD: mean difference.

**Grouped by Severity of COVID-19 infection**

This figure represents forest plot of sensitivity analysis for RVD meta-analysis grouped by COVID-19 severity. Gunay et al. (2021) was at high risk of bias for this outcome and excluding this study did not change the statistical significance of the summary estimate. A random effect model was used. RVD: right ventricular diameter; MD: mean difference.

**Grouped by status of Comorbid diseases**

This figure represents forest plot of sensitivity analysis for RVD meta-analysis grouped by comorbid disease. Gunay et al. (2021) was at high risk of bias for this outcome and excluding this study changed the statistical significance of the summary estimate. A positive result is in favor of higher amounts in post-COVID patients compared to controls. A fixed effect model was used. RVD: right ventricular diameter; MD: mean difference.

**sPAP**

This figure represents forest plot of sensitivity analysis for sPAP meta-analysis grouped by COVID-19 severity. Küçük et al. (2022) was at high risk of bias for this outcome and excluding this study did not change the statistical significance of the summary estimate. A fixed effect model was used. sPAP: systolic pulmonary artery pressure; MD: mean difference.

**Grouped by status of Comorbid disease**

This figure represents forest plot of sensitivity analysis for sPAP meta-analysis grouped by comorbid disease. De et al. (2023) was at high risk of bias for this outcome and excluding this study did not change the statistical significance of the summary estimate. A fixed effect model was used. sPAP: systolic pulmonary artery pressure; MD: mean difference.
