# Supplementary material for: A deeper look at long-term effects of COVID-19 on myocardial function in survivors with no prior heart diseases: a GRADE approach systematic review and meta-analysis
Source: Front Cardiovasc Med. 2024 Nov 19;11:1458389. doi: 10.3389/fcvm.2024.1458389 (PMC11611865; doi:10.3389/fcvm.2024.1458389)
Supplement: Supplementary S6 Document — Scatter plots of meta-regression. [file Datasheet6.docx]

**E/A ratio**

**A**

**B**

Meta-regression analysis of E/A ratio with age and BMI as moderators. Panel **A** represents scatter plot showing the correlation between post-COVID patients’ age and MDs of E/A ratio. Panel **B** represents scatter plot showing the correlation between post-COVID patients’ BMI and MDs of E/A ratio.

**LV-GLS**

**A**

**B**

Meta-regression analysis of LV-GLS with age and BMI as moderators. Panel **A** represents scatter plot showing the correlation between post-COVID patients’ age and MDs of LV-GLS. Panel **B** represents scatter plot showing the correlation between post-COVID patients’ BMI and MDs of LV-GLS.

**LAVI**

**A**

**B**

Meta-regression analysis of LAVI with age and BMI as moderators. Panel **A** represents scatter plot showing the correlation between post-COVID patients’ age and MDs of LAVI. Panel **B** represents scatter plot showing the correlation between post-COVID patients’ BMI and MDs of LAVI.

**LAD**

**A**

**B**

Meta-regression analysis of LAD with age and BMI as moderators. Panel **A** represents scatter plot showing the correlation between post-COVID patients’ age and MDs of LAD. Panel **B** represents scatter plot showing the correlation between post-COVID patients’ BMI and MDs of LAD.

**RV-GLS**

**A**

**B**

Meta-regression analysis of RV-GLS with age and BMI as moderators. Panel **A** represents scatter plot showing the correlation between post-COVID patients’ age and MDs of RV-GLS. Panel **B** represents scatter plot showing the correlation between post-COVID patients’ BMI and MDs of RV-GLS.

**A**

**TAPSE**

**B**

Meta-regression analysis of TAPSE with age and BMI as moderators. Panel **A** represents scatter plot showing the correlation between post-COVID patients’ age and MDs of TAPSE. Panel **B** represents scatter plot showing the correlation between post-COVID patients’ BMI and MDs of TAPSE.

**sPAP**

**A**

**B**

Meta-regression analysis of sPAP with age and BMI as moderators. Panel **A** represents scatter plot showing the correlation between post-COVID patients’ age and MDs of sPAP. Panel **B** represents scatter plot showing the correlation between post-COVID patients’ BMI and MDs of sPAP.
