# Supplementary material for: A deeper look at long-term effects of COVID-19 on myocardial function in survivors with no prior heart diseases: a GRADE approach systematic review and meta-analysis
Source: Front Cardiovasc Med. 2024 Nov 19;11:1458389. doi: 10.3389/fcvm.2024.1458389 (PMC11611865; doi:10.3389/fcvm.2024.1458389)
Supplement: Supplementary S7 Document — Funnel plots of meta-analysis. [file Datasheet7.docx]

**Begg’s test and Egger’s test for publication bias**

| **Analysis value** | **Study (n)** | **Egger’ test** | | **Beggs’ test** | | | |
| --- | --- | --- | --- | --- | --- | --- | --- |
|  |  | ***i*** | ***p*** | ***k*** | ***t*** | ***z*** | ***p*** |
| **LVEDD** | 27 | *1.520* | *0.08* | *29.00* | *0.079* | *0.58* | *0.559* |
| **LVEDV** | 11 | *3.09* | *0.144* | *5.00* | *0.072* | *0.311* | *0.755* |
| **LVESD** | 16 | *-0.76* | *0.48* | *-24.00* | *-0.191* | *1.035* | *0.300* |
| **LVESV** | 6 | *2.22* | *0.167* | *5.00* | *0.266* | *0.751* | *0.452* |
| **IVSD** | 19 | *-0.700* | *0.696* | *-17.00* | *-0.09* | *0.559* | *0.575* |
| **PWD** | 19 | *0.615* | *0.512* | *11.00* | *0.05* | *0.349* | *0.726* |
| **LVEF** | 32 | *-1.60* | ***0.003*** | *-176* | *-0.324* | *2.61* | ***0.009*** |
| **LV-GLS** | 26 | *-2.27* | *0.161* | *-78.00* | *-0.236* | *1.69* | *0.08* |
| **E/A** | 21 | *-1.03* | *0.337* | *-30.00* | *-0.138* | *0.875* | *0.381* |
| **E/e’** | 27 | *1.56* | *0.176* | *-3.00* | *-0.005* | *0.04* | *0.966* |
| **E wave** | 13 | *0.781* | *0.494* | *6.00* | *0.06* | *0.305* | *0.760* |
| **A wave** | 10 | *0.395* | *0.615* | *-9.00* | *-0.177* | *0.715* | *0.472* |
| **LAD** | 12 | *3.42* | ***0.02*** | *30.00* | *0.439* | *1.40* | ***0.04*** |
| **LAVI** | 15 | *-3.70* | ***0.02*** | *-21.00* | *-0.190* | *0.989* | *0.322* |
| **RV-GLS** | 16 | *-1.23* | *0.438* | *-7.00* | *-0.05* | *0.270* | *0.787* |
| **RV-diameter** | 16 | *0.438* | *0.853* | *-14.00* | *-0.108* | *0.585* | *0.558* |
| **TAPSE** | 27 | *0.712* | *0.579* | *60.00* | *0.168* | *1.22* | *0.218* |
| **sPAP** | 12 | *9.74* | ***0.002*** | *38.00* | *0.560* | *2.53* | ***0.011*** |

**LVEDV**

Publication bias analysis for LVEDV. The funnel plot graph showed an asymmetrical inverted funnel-plot. LVEDV: left ventricular end-diastolic volume.

**LVEDD**

Publication bias analysis for LVEDD. The funnel plot graph showed an asymmetrical inverted funnel-plot. LVEDD: left ventricular end-diastolic diameter.

**LVESD**

Publication bias analysis for LVESD. The funnel plot graph showed an asymmetrical inverted funnel-plot. LVESD: left ventricular end-systolic diameter.

**LVESV**

Publication bias analysis for LVESV. The funnel plot graph showed an asymmetrical inverted funnel-plot. LVESV: left ventricular end-systolic volume.

**IVSD**

Publication bias analysis for IVSD. The funnel plot graph showed an asymmetrical inverted funnel-plot. IVSD: interventricular septal diameter.

**PWD**

Publication bias analysis for PWD. The funnel plot graph showed an asymmetrical inverted funnel-plot. PWD: posterior wall diameter.

**LVM**

Publication bias analysis for LVM. The funnel plot graph showed an asymmetrical inverted funnel-plot. LVM: left ventricular mass.

**LVMI**

Publication bias analysis for LVMI. The funnel plot graph showed an asymmetrical inverted funnel-plot. LVMI: left ventricular mass index.

**LAVI**

Publication bias analysis for LAVI. The funnel plot graph showed an asymmetrical inverted funnel-plot. LAVI: left atrium volume index.

**LAVI-Trim and Fill**

Duval and Tweedie's trim and Fill Funnel plot for LAVI. It was determined that 5 studies needed to be imputed on the right side of the scatter plot, resulting in a summary effect size of 1.92, with a 95%CI of (0.689, 3.168).

**LAD**

Publication bias analysis for LAD. The funnel plot graph showed an asymmetrical inverted funnel-plot. LAD: left atrium diameter.

**LAD-trim and Fill**

Duval and Tweedie's trim and Fill Funnel plot for LAD. It was determined that 4 studies needed to be added on the left side of the scatter plot. The adjusted effect size was calculated 0.800 with a 95%CI of (-0.115, 1.716).

**LVEF**

Publication bias analysis for LVEF. The funnel plot graph showed an asymmetrical inverted funnel-plot. LVEF: left ventricular ejection fraction.

**LVEF-Trim and Fill**

Duval and Tweedie's trim and Fill Funnel plot for LVEF. It was determined that 9 studies needed to be added on the right side of the scatter plot for LVEF analysis. Following this adjustment, the effect size was calculated to be -0.120, with a 95%CI of (-0.711, 0.471).

**LV-GLS**

Publication bias analysis for LV-GLS. The funnel plot graph showed an asymmetrical inverted funnel-plot. LV-GLS: left ventricular global longitudinal strain.

**E/A**

Publication bias analysis for E/A ratio. The funnel plot graph showed an asymmetrical inverted funnel-plot. E/A: the ratio of peak velocity blood flow from left ventricular relaxation in early diastole (the E wave) to peak velocity flow in late diastole caused by atrial contraction (the A wave).

**E/e’**

Publication bias analysis for E/e’ ratio. The funnel plot graph showed an asymmetrical inverted funnel-plot. E/e’: Ratio of ratio of E wave to early diastolic mitral annular velocity (e’).

**E wave**

Publication bias analysis for E wave. The funnel plot graph showed an asymmetrical inverted funnel-plot. E wave: peak velocity blood flow from left ventricular relaxation in early diastole.

**A wave**

Publication bias analysis for A wave. The funnel plot graph showed an asymmetrical inverted funnel-plot. A wave: peak velocity flow in late diastole caused by atrial contraction.

**RV-GLS**

Publication bias analysis for RV-GLS. The funnel plot graph showed an asymmetrical inverted funnel-plot. RV-GLS: right ventricular global longitudinal strain.

**RV-MPI**

Publication bias analysis for RV-MPI. The funnel plot graph showed an asymmetrical inverted funnel-plot. RV-MPI: right ventricular myocardial performance index.

**RVD**

Publication bias analysis for RVD. The funnel plot graph showed an asymmetrical inverted funnel-plot. RVD: right ventricular diameter.

**RAD**

Publication bias analysis for RAD. The funnel plot graph showed an asymmetrical inverted funnel-plot. RAD: right atrium diameter.

**TAPSE**

Publication bias analysis for TAPSE. The funnel plot graph showed an asymmetrical inverted funnel-plot. TAPSE: tricuspid annular plane systolic excursion.

**SPAP**

Publication bias analysis for sPAP. The funnel plot graph showed an asymmetrical inverted funnel-plot. sPAP: systolic pulmonary artery pressure.

**SPAP-Trim and Fill**

Duval and Tweedie's trim and Fill Funnel plot for sPAP. It was determined that 5 studies required imputation on the left side of the scatter plot, leading to a summary effect size of 1.29, with a 95%CI of (0.882, 1.717).
